# Anticancer, Antimicrobial, and Antioxidant Activities of Organodiselenide-Tethered Methyl Anthranilates

**DOI:** 10.3390/biom12121765

**Published:** 2022-11-27

**Authors:** Batool Al-Abdallah, Yasair S. Al-Faiyz, Saad Shaaban

**Affiliations:** 1 Chemistry Department, College of Science, King Faisal University, Al-Ahsa 31982, Saudi Arabia; 2 Chemistry Department, Faculty of Science, Mansoura University, Mansoura 35516, Egypt

**Keywords:** anticancer, anthranilic acid, antimicrobial, organoselenium, antioxidant, organodiselenide

## Abstract

Novel methyl anthranilate-based organodiselenide hybrids were synthesized, and their chemical structures were confirmed by state-of-the-art spectroscopic techniques. Their antimicrobial properties were assessed against *Staphylococcus aureus*, *Escherichia coli*, and *Candida albicans* microbial strains. Moreover, the antitumor potential was estimated against liver and breast carcinomas, as well as primary fibroblast cell lines. The *Staphylococcus aureus* and *Candida albicans* strains were more sensitive than *Escherichia coli* toward the OSe compounds. Interestingly, methyl 2-amino-5-(methylselanyl) benzoate (**14**) showed similar antifungal activity to the standard drug clotrimazole (IA% = 100%) and manifested promising antibacterial activity against *E. coli* (IA% = 91.3%) and *S. aureus* (IA% = 90.5%). Furthermore, the minimum inhibitory concentration experiments confirmed the antimicrobial activity of the OSe **14**, which in turn was comparable to clotrimazole and ampicillin drugs. Interestingly, the anticancer properties were more pronounced in the HepG2 cells. The OSe **14** was the most cytotoxic (IC_50_ = 3.57 ± 0.1 µM), even more than the Adriamycin drug (IC_50_ = 4.50 ± 0.2 µM), and with therapeutic index (TI) 17 proposing its potential selectivity and safety. Additionally, OSe compounds **14** and dimethyl 5,5′-diselanediylbis(2-aminobenzoate) (**5**) exhibited promising antioxidants in the 2,2′-azino-bis (3-ethylbenzothiazoline-6-sulfonic acid (ABTS) and 2,2-diphenyl-1-picrylhydrazyl (DPPH) in vitro assays with 96%, 92%, 91%, and 86% radical scavenging activities compared to 95% by vitamin C in the DPPH and ABTS assays, respectively. These results point to promising antimicrobial, anticancer, and antioxidant activities of OSe **14** and **5** and warrant further studies.

## 1. Introduction

Organoselenium (OSe) compounds have raised much more concern than their inorganic selenium (Se) peers due to their numerous pharmaceutical applications as well as improved pharmacokinetics and bioavailability [1,2]. The non-metal Se element belongs to the oxygen (a.k.a. chalcogen) group. Se has a leading function in immune systems protection and cancer cell proliferation inhibition [3,4]. Various infectious and autoimmune illnesses are somehow linked with the deficiency of Se [4]. On the other hand, Se dietary supplementation was associated with improving different inflammatory disorders and chemoprevention of various types of cancer [5]. Within this context, OSe compounds have shown antiviral, antimicrobial, and antioxidant activities [6]. Due to their diverse pharmacological activities, organodiselenide (OSe_2_) compounds are among the most evaluated OSe derivatives [7,8,9]. Therefore, researchers have recently been directed to disclose new OSe_2_ for biological testing. For instance, diphenyl diselenide (**I**) is an antidepressant and an antioxidant agent [10]. Moreover, compounds 1,2-bis(chloropyridazinyl) diselenide (**II**) and bis(4-amino-3-carboxyphenyl) diselenide (**III**) were able to inhibit the growth of breast cancer cells and manifest efficient anti-leishmanial properties, respectively [5].

**Figure 1:**
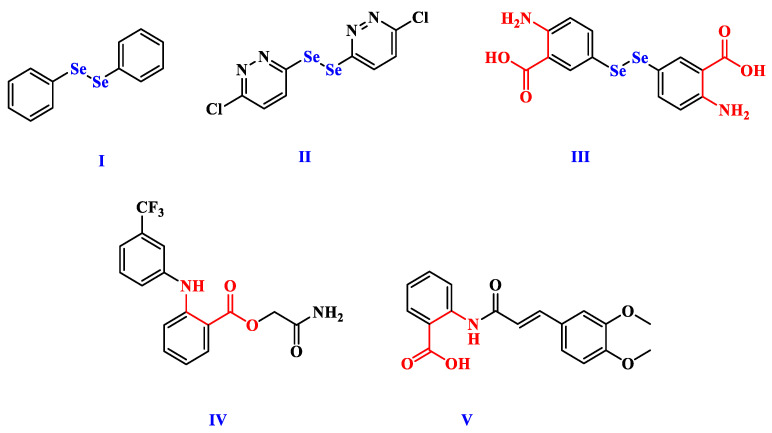
The structure of biologically relevant organoselenium and anthranilic acid analogs.

Furthermore, the anthranilic acid-based derivatives captured our interest as they were found in the backbone of several drugs (e.g., colfenamate (**IV**) and tranilast (**V**)) and natural products (e.g., essential oils) (Figure 1) and manifested different biological applications (e.g., anti-inflammatory, anticancer, antibacterial, and antioxidant) [11]. Therefore, the anthranilate scaffold offers a promising pharmacophore for developing novel drugs designed to target different diseases [12].

Accordingly, combining the pharmacologically active anthranilate scaffolds and OSe_2_ would promote overall biological activities. Therefore, we aim to design and synthesize new OSe_2_-tethered anthranilate hybrids and evaluate their respective antimicrobial, anticancer, and antioxidant properties.

## 2. Materials and Methods

### 2.1. Material and Methods

All reagents and solvents used in this study were purchased from Sigma and used without purification. Melting points (MP) were measured on the Gallenkamp apparatus in degrees centigrade. The IR spectra (KBr, λ_max_.cm^−1^) were recorded on a Mattson spectrophotometer (5000 FTIR) at King Faisal University. The ^1^HNMR and ^13^CNMR spectra were measured using Varian Spectrophotometer (400 MHz), employing DMSO-d6 as the solvent and TMS internal standard at Mansoura University. The chemical shifts (δ, ppm) were recorded regarding the solvent residual peaks. GC-MS-QP-100 EX Shimadzu apparatus was used for mass measurements at Cairo University. All biological tests were carried out at the Faculty of Pharmacy, Mansoura University. All cell lines and microorganisms were purchased from the VACSERA Company (ATCC), Cairo, Egypt. DPPH and ABTS probes were obtained from Sigma. Compound **2** was synthesized following our literature method [13]. Copies of ^1^H & ^13^CNMR spectra IR and MS can be found in the Supplementary Materials Section S2.

### 2.2. Chemistry

Procedure I: The preparation of selenocyanate **4** and diselenide **5**.

Methyl 2-amino-5-selenocyanatobenzoate (**4**) was synthesized from the reaction of methyl 2-aminobenzoate with triselenium dicyanide prepared in situ from malononitrile and selenium dioxide in 96% yields. Briefly, selenium dioxide (30 mmol, 3300 mg) was added to malononitrile (15 mmol, 1000 mg) in 10 mL DMSO, and the mixture was stirred for 20 min at room temperature. Next, the mixture was filtered off to get rid of any formed black selenium, and methyl 2-aminobenzoate (12.5 mmol, 1800 mg) was then added, and the reaction mixture was stirred for a further 2 hr at room temperature. Finally, adding 10 g of ice terminated the reaction, and the formed precipitate was filtered, washed several times with H_2_O and sodium carbonate solution, dried, and recrystallized from petroleum ether.

Compound dimethyl 5,5-diselanediylbis(2-aminobenzoate) (**5**) was synthesized from the reaction of **4** and sodium hydroxide in 92% yields. Briefly, compound **4** (4 mmol, 1000 mg) was dissolved in EtOH (20 mL), and then sodium hydroxide (4 mmol, 160 mg) was added. The reaction mixture was stirred for 2 h at room temperature, and the resulting precipitate was filtered, washed several times with H_2_O, and recrystallized from chloroform.

Procedure II: The preparation of the OSe azo dyes **6**.

Dimethyl 5,5′-diselanediylbis(2-aminobenzoate) (**5**) (2 mmol,916.4 mg) was dissolved in aqueous HCl (4 mL) and cooled to 0–5 °C. Sodium nitrite (4.4 mmol, 331 mg in 10 mL H_2_O) was then added to the previously prepared solution while maintaining the temperature at 0–5 °C. The freshly prepared diazonium salt solution was then added dropwise to a cooled and stirred solution of methylene compounds, e.g., ethyl cyanoacetate (4.4 mmol) and sodium acetate (2000 mg) dissolved in (10 mL) of H_2_O. After stirring the reaction mixture for 3 h at 0–5 °C, the resulting precipitate was filtered, washed with H_2_O, and recrystallized from EtOH.

Procedure III: The preparation of OSe amide-acids **11** and **13**.

Dimethyl 5,5′-diselanediylbis(2-aminobenzoate) (**5**) (2 mmol) was added to a stirred solution of maleic or succinic anhydride (4.4 mmol) in dry toluene (15 mL). The mixture was vigorously stirred, and the formed precipitate was separated by filtration. The residue was washed with hot toluene, dried under reduced pressure, and recrystallized from EtOH.

Procedure IV: The preparation of cyclic imide **12**.

A mixture containing amide acids (**11**) (0.5 mmol) and sodium acetate (250 mg) in acetic anhydride (5 mL) was heated at 60–65 °C for three h. The reaction mixture was poured into ice H_2_O and neutralized with sodium carbonate. The precipitate formed was collected by filtration, washed 3x with sodium carbonate solution, and recrystallized from EtOH.

Procedure V: The preparation of organic selenides via reduction **14**, **15**, and **16**.

Dimethyl 5,5′-diselanediylbis (2-aminobenzoate) (**5**) (2 mmol) and appropriate halo derivatives (4.4 mmol) were dissolved in EtOH (25 mL). Next, NaBH_4_ (6 mmol, 226.98 mg) was added portion-wise over one h. Then the reaction was stirred for an additional 2 h. Finally, the organic layer was dried and evaporated under a vacuum.

Synthesis of 2-amino-5-selenocyanatobenzoic acid (**3**) [9,13].

Malononitrile (15 mmol) was dissolved in (10 mL) of DMF, then SeO_2_ (30 mmol) was added. After 15 min, the reaction was filtered, and anthranilic acid (12.5 mmol) was added. The reaction was further stirred for another 15 min and added ice to the mixture. It was kept refrigerated overnight. The substance that formed was filtered off, dried, and recrystallized from DMF: H_2_O (1:1). Product was isolated as a brown solid; Yield: 76%; MP = 180 (decomposition)°C. ^1^HNMR (400 MHz, DMSO-d6) δ = 7.86(d, J = 14.9 Hz, 1H, Ar-H), 7.42(d, J = 2.7 Hz, 1H), 6.71(dd, J1 = Hz, J2 = 14.9 Hz, H1H, Ar-H); MS (ESI): *m*/*z* = found 241.29 [M+]; calcd 241.19 [M+]; Elemental Analysis C_8_H_6_N_2_O_2_Se: C, 39.81; H, 2.50; N, 11.60.

Synthesis of methyl 2-amino-5-selenocyanatobnzoate (**4**).

Methyl 2-amino-5-selenocyanatobenzoate (**4**) was synthesized following procedure I from methyl 2-aminobenzoate (12.5 mmol, 1800 mg) with triselenium dicyanide prepared in situ from malononitrile (15 mmol, 1000 mg) and selenium dioxide (30 mmol, 3300 mg). It was isolated as reddish solid; yield: 3072 mg (96%); MP = 118–119 °C; Rf = 0.4(petroleum ether/ethyl acetate 4:2). IR(KBr): λ_max_.cm^−1^: 3475, 3366, 2946, 2147, 1691. ^1^HNMR (400 MHz, DMSO-d6) δ8.02(s, 1H, Ar-H), 7.57(d, J = 8.8 Hz, 1H, Ar-H), 7.08(d, 1H, Ar-H), 6.84(s, 2H, NH_2_), 3.82(s, 3H, OCH_3_). ^13^C NMR (101 MHz, DMSO-d6) δ166, 152, 140, 137, 118, 109, 105, 105, 51. MS (EI, 70 ev) *m*/*z* (%) = 259.35(M+3H, 2.39), 117(29.02), 87(26.6), 75(2.70), 59(100.0, base peak).

Synthesis of dimethyl 5,5′-diselanediylbis(2-aminobenzoate) (**5**)

Compound dimethyl 5,5-diselanediylbis(2-aminobenzoate) (**5**) was synthesized following the procedure I from methyl 2-amino-5-selenocyanatobnzoate (4 mmol, 1000 mg) and sodium hydroxide (4 mmol, 160 mg) in EtOH (20 mL). Dimethyl 5,5′-diselanediylbis(2-aminobenzoate) (**5**) appeared as a single compound on TLC and was isolated as a yellow solid; yield: 1692.43 mg (92%); MP = 138–139 °C; Rf = 0.5(petroleum ether/ethyl acetate 4:3). IR(KBr): λ_max_.cm^−1^: 3455, 3344, 2931, 1684. ^1^HNMR (400 MHz, DMSO) δ7.70(s,2H, Ar-H), 7.44(d, *J* = 8.6 Hz, 2H, Ar-H), 7.00(s, 4H,2NH_2_), 6.77(d, *J* = 8.7 Hz, 2H, Ar-H), 3.74(s, 6H,2OCH_3_). ^13^CNMR (101 MHz, DMSO-d_6_) δ167, 151, 140, 138, 117, 113, 108, 51. MS (EI, 70 ev) *m*/*z* (%) = 460.15(M+H, 20.76), 459.15(M, 5.20) or 230(24.42), 119(9.45), 91(100.0, base peak), 65(8.88).

Synthesis of dimethyl 5,5′-diselanediylbis(2-(2-(1-cyano-2-ethoxy-2-oxoethylidene) hydrazinyl) benzoate) (**6**).

Compound **6** was synthesized following procedure II from dimethyl 5,5′-diselanediylbis(2-aminobenzoate) (**5**) (2 mmol, 916.4 mg) and ethyl cyanoacetate (4.4 mmol, 0.46 mL). It was isolated as yellow crystal; yield: 1147 mg (81%); MP = 105–106 °C; Rf = 0.4(petroleum ether/ethyl acetate 4:4). IR(KBr): λ_max_.cm^−1^: 3146, 2984, 2138, 1693. ^1^HNMR (400 MHz, DMSO-d_6_) δ12.56(s, 2H,2NH), 8.13(s, 2H, Ar-H), 7.83(d, *J* = 8.8 Hz, 2H, Ar-H), 7.70(d, *J* = 8.6 Hz, 2H, Ar-H), 4.4–4.29(q, 4H, 2OCH_2_), 3.96–3.88(s, 6H,2OCH_3_), 1.32(t, *J* = 7.2 Hz, 6H, 2CH_3_). MS (EI, 70ev) *m*/*z* (%) = 708.25(M, 18.03), 354(31.30), 111.20(2.62), 90.10(12.21), 59.10(100.0, base peak).

Synthesis of dimethyl 5,5′-diselanediylbis(2-formamidobenzoate) (**7**).

Acetic formic anhydride (freshly prepared, 36.3 mmol, 3.2 mL) was added dropwise at room temperature to a solution of dimethyl 5,5′-diselanediylbis(2-aminobenzoate) (**5**) (1 mmol, 458.2 mg) in THF solution (10 mL). The reaction progress was monitored with TLC. It was isolated as an orange solid and recrystallized from EtOH, yielding: 201.2 mg (39%); MP = 195–196 °C; Rf = 0.35(petroleum ether/ethyl acetate 4:2). IR(KBr): λ_max_.cm^−1^_:_ 3255, 2951, 1681, 1573. ^1^HNMR (400 MHz, DMSO-d_6_) δ10.42(s, 2H,2NH), 8.39(s, 2H,2CHO), 8.36–8.12(s, 2H, Ar-H), 7.92(d, 2H, Ar-H), 7.60(d, *J* = 6.2 Hz, 2H, Ar-H), 3.75(s, 6H, 2OCH_3_). ^13^CNMR (101 MHz, DMSO-d_6_) δ166, 161, 139, 138, 135, 124, 122, 118, 53. MS (EI, 70 ev) *m*/*z* (%) = 516.10(M, 25.78), 515.15(M-1H, 8.92), 258.10(6.08), 229(13.95), 198(100.0basepeak), 134.20(7.40).

Synthesis of dimethyl 5,5′-diselanediylbis(2-acetamidobenzoate) (**8**).

A mixture of compound **5** (2 mmol,916.4 mg) and acetic anhydride (8 mL) in a 50 mL round-bottom flask fitted with a condenser was heated in an oil bath at 65–70 °C for 7 h. The reaction mixture was allowed to cool at room temperature, poured on ice-cold H_2_O, and neutralized with a sodium carbonate solution. The formed solid was collected by filtration, washed with H_2_O, and then recrystallized from EtOH. Compound **8** was isolated as a yellow solid; yielding: 462.4 mg (85%); MP = 186–187 °C; Rf = 0.6(petroleum ether/ethyl acetate 4:4). IR(KBr): λ_max_.cm^−1^: 3281, 2934, 1700, 1682. ^1^HNMR (400 MHz, DMSO-d_6_) δ10.57(s, 2H, 2NH), 8.18(s, 2H, Ar-H), 8.00(d, *J* = 23.7 Hz, 2H, Ar-H), 7.84(d, *J* = 7.7 Hz, 2H, Ar-H), 3.84(s, 6H, 2OCH_3_), 2.13(s, 6H,2CH_3_). MS (EI, 70 ev) *m*/*z* (%) = 544.30(M, 40.15), 272.15(17.66), 230(100.0 base peak), 192.1(1.81), 90.15(14.52).

Synthesis of dimethyl 5,5′-diselanediylbis(2-(2-chloroacetamido) benzoate) (**9**).

To a cold solution of compound (**5**) (2 mmol, 916.4 mg) in dry acetone (20 mL) and K_2_CO_3_ (2000 mg), chloroacetyl chloride (4.4 mmol, 0.34 mL) was added dropwise with stirring at 0–5 °C. Stirring was continued for 5 h, and the reaction mixture was poured onto ice-cold H_2_O. The resulting precipitate was collected, dried, and recrystallized from MeOH to afford the corresponding chloroacetamide. Compound **9** was isolated as a light-yellow solid; yielding: 569 mg (93%); MP = 150–152 °C; Rf = 0.6(petroleum ether/ethyl acetate 4:3). IR(KBr): λ_max_.cm^−1^: 3231, 2952, 1698, 1575, 777. ^1^HNMR (400 MHz, DMSO-d_6_) δ11.39(s, 2H,2NH), 8.40–8.35(s, 2H, Ar-H), 8.12(d, *J* = 1.9 Hz, 2H, Ar-H), 7.92(dd, *J* = 8.6, 1.9 Hz, 2H, Ar-H), 4.32(s, 4H,2CH_2_), 3.87(s, 6H,2OCH_3_). ^13^CNMR (101 MHz, DMSO) δ167, 165, 139, 138, 135, 125, 122, 118, 53, 43. MS (EI, 70 ev) *m*/*z* (%) = 611.15(M, 33.33), 612.10(100.0 base peak), 105(0.43), 92(0.42), 77.05(6.51).

Synthesis of dimethyl 5,5′-diselanediylbis(2-(2-phenoxyacetamido) benzoate) (**10**).

To compound **5** in dry ether (15 mL), phenoxy acetyl chloride (2.2 mmol, 0.30 mL) was added dropwise. The reaction was stirred at room temperature for 5 h. The solvent was then evaporated; the formed residue was dissolved in dichloromethane, and the organic layer was extracted using 10% HCl and 10% NaOH. The organic layer was filtered over anhydrous sodium sulfate and evaporated. Compound **10** was isolated as a yellow solid and recrystallized from EtOH, yielding: 400.4 mg (55%); MP = 152–154 °C; Rf = 0.4(petroleum ether/ethyl acetate 4:3). IR(KBr): λ_max_.cm^−1^: 3249, 2921, 2852, 1686, 1571. ^1^HNMR (400 MHz, DMSO-d_6_) δ12.51(s, 2H, 2NH), 8.65(s, 3H, Ar-H), 8.13(dd, *J* = 9.9, 1.9 Hz, 3H, Ar-H), 7.94 (dd, *J* = 12.8, 5.9 Hz, 3H, Ar-H), 7.50–7.33(q, 4H, Ar-H), 7.05(t, 3H, Ar-H), 4.79(s, 4H, 2CH_2_), 3.90(s, 6H, 2OCH_3_). ^13^CNMR (101 MHz, DMSO-d_6_) δ167, 167, 157, 140, 139, 135, 130, 124, 122, 121, 117, 115, 67, 53. MS (EI, 70 ev) *m*/*z* (%) = 728.40(M, 0.02), 107.15(2.50), 90.15(0.28), 77.10(5.51), 59.10(100.0 base peak).

Synthesis of 4,4′-((diselanediylbis(2-(methoxycarbonyl)-4,1-phenylene)) bis(azanediyl)) bis (4-oxobutanoic acid (**11**).

Compound **11** was synthesized following procedure III from the reaction of dimethyl 5,5-diselanediylbis(2-aminobenzoate) (**5**) (2 mmol, 916.4 mg) and succinic anhydride (4.4 mmol, 440.3 mg). It was isolated as a yellow solid; yielding: 910.8 mg (69%); MP = 150–152 °C; Rf = 0.1(petroleum ether/ethyl acetate 4:4). IR(KBr): λ_max_.cm^−1^: 3246, 2949, 1735, 1687. ^1^HNMR (400 MHz, DMSO-d_6_) δ12.22(s, 2H-OH), 10.67(s,2H, 2NH), 8.24(s, 2H, Ar-H), 8.01 (d, 2H, Ar-H), 7.82(d, *J* = 8.7 Hz, 2H, Ar-H), 3.78(s, 6H, OCH_3_), 2.64(t, 4H, 2CH_2_), 2.55(t, 4H, 2CH_2_).^13^CNMR (101 MHz, DMSO-d_6_) δ173, 170, 166, 139, 138, 134, 123, 121, 118, 52, 31, 28. MS (EI, 70 ev) *m*/*z* (%) = 462(M-198, 6.73), 460(17.67), 199(11.05), 101(21.56), 91.15(100.0, base peak).

Synthesis of dimethyl 5,5′-diselanediylbis(2-(2,5-dioxopyrrolidin-1-yl) benzoate (**12**).

Compound **12** was synthesized following procedure VI from the reaction of 4,4′-((diselanediylbis(2-(methoxycarbonyl)-4,1-phenylene)) bis(azanediyl)) bis (4-oxobutanoic acid (**11**) (0.5 mmol, 330 mg) and (5 mL) acetic anhydride. It was isolated as light-yellow solid; yielding: 121.68 mg (39%); MP = 100 °C; Rf = 0.4(DCM/MeOH 5%). IR(KBr): λ_max_.cm^−1^: 3261, 2950, 1684, 1572. ^1^HNMR (400 MHz, DMSO-d_6_) δ8.17(s, 2H, Ar-H), 8.01(d, *J* = 25.0 Hz, 2H, Ar-H), 7.84(d, *J* = 8.6 Hz, 2H, Ar-H), 3.81(s, 6H, 2OCH_3_), 2.14(t, 8H, 4CH_2_). ^13^CNMR (101 MHz, DMSO-d_6_) δ169, 167, 140, 138, 135, 124, 122, 119, 53, 25. MS (EI, 70 ev) *m*/*z* (%) = 624.20(M, 19.34), 312.15(6.43), 63.10(3.53), 59.05(3.07), 55.05 (100.0, base peak).

Synthesis of (2Z,2’Z)-4,4′-((diselanediylbis(2-(methoxycarbonyl)-4,1-phenylene)) bis(azanediyl)) bis (4-oxobut-2-enoic acid) (**13**).

Compound **13** was synthesized following procedure III from the reaction of dimethyl 5,5-diselanediylbis(2-aminobenzoate) (**5**) (2 mmol, 916.4 mg) and maleic anhydride (4.4 mmol, 433.84 mg). It was isolated as yellow solid; yielding: 983.85 mg (75 %); MP = 149.5–151 °C; Rf = 0.1(petroleum ether/ethyl acetate 4:4). IR(KBr): λ_max_.cm^−1^: 3346, 2952, 1717, 1696. ^1^HNMR (400 MHz, DMSO-d_6_) δ13.01(s, 2H, OH), 10.85(s, 2H, NH), 8.25(s, 2H, Ar-H), 8.09–7.97(d, 2H, Ar-H), 7.85(dd, *J* = 23.2, 12.0 Hz, 2H, Ar-H), 6.59(d, *J* = 11.9, 6.2 Hz, 2H, = CH), 6.36(d, *J* = 11.9 Hz, 2H, CH = ), 3.83(s, 6H, OCH_3_). ^13^CNMR (101 MHz, DMSO-d_6_) δ166, 166, 163, 139, 137, 132, 130, 128, 124, 122, 117, 51. MS (EI, 70 ev) *m*/*z* (%) = 662(M-9H, 2.03), 71.05(6.51), 67(3.36), 75(1.31), 54.05(100.0, base peak).

Synthesis of methyl 2-amino-5-(methylselanyl) benzoate (**14**).

Compound **14** was synthesized following procedure V from dimethyl 5,5′-diselanediylbis (2-aminobenzoate) (**5**) (2 mmol, 916.4 mg) and methyl iodide (4.4 mmol, 0.27 mL). It was isolated as a brown solid; yielding: 401.8 mg (82%); Rf = 0.6(petroleum ether/ethyl acetate 4:2). IR(KBr): λ_max_.cm^−1^: 3474, 2948, 1686, 1606. ^1^HNMR (400 MHz, DMSO-d_6_) δ8.25–7.65(s,1H, Ar-H), 7.65–7.20(d, 1H, Ar-H), 6.77(d, 1H, Ar-H), 6.75(s, 2H, NH_2_), 3.62(s, 3H, OCH_3_), 2.2 (s, 3H, CH_3_). ^13^CNMR (101 MHz, DMSO-d_6_) δ167, 151, 138, 135, 118, 113, 109, 51, 9. MS (EI, 70 ev) *m*/*z* (%) = 245.10(M, 100.0, base peak), 230(38.06), 186(10.46), 170(35.08), 91(91.10).

Synthesis of methyl 2-amino-5-(benzylselanyl) benzoate (**15**).

Compound **15** was synthesized following procedure V from dimethyl 5,5′-diselanediylbis(2-aminobenzoate) (**5**) (2 mmol, 916.4 mg) and benzyl chloride (4.4 mmol, 0.5 mL). It was isolated as a light brown solid; yielding: 597.06 mg (93%); MP = 78 °C; Rf = 0.6(petroleum ether/ethyl acetate 4:3). IR(KBr): λ_max_.cm^−1^: 3461, 3353, 2977, 1671, 1620. ^1^HNMR (400 MHz, DMSO-d_6_) δ7.81–7.65(m,1H, Ar-H), 7.30(d, *J* = 8.6 Hz, 1H, Ar-H), 7.23(t,2H, Ar-H), 7.19(q, 2H, Ar-H), 7.14(t, 3H, Ar-H), 6.82(s, 2H, NH_2_), 6.70(d, 1H, Ar-H), 3.77(s, 3H, OCH_3_), 1.29(s, 2H, SeCH_2_). ^13^CNMR (101 MHz, DMSO-d_6_) δ167, 151, 141, 139, 137, 129, 128, 126, 117, 112, 109, 51, 32. MS (EI, 70 ev) *m*/*z* (%) = 321.20(M, 46.87), 244.10(2.77), 230(34.88), 150.15(1.02), 91.05(100.0, base peak).

Synthesis of methyl 2-amino-5-((2-oxo-2-(phenylamino) ethyl) selanyl) benzoate (**16**).

Compound **16** was synthesized following procedure V from dimethyl 5,5′-diselanediylbis (2-aminobenzoate) (**5**) (2 mmol, 916 mg) and 2-chloro-N-phenylacetamide (4.4 mmol, 743.6 mg). It was isolated as a violet solid; yielding: 698.8 mg (96%); MP = 126–128.5 °C; Rf = 0.5(petroleum ether/ethyl acetate 4:2). IR(KBr): λ_max_.cm^−1^: 3466, 3246, 2945, 1678. ^1^H NMR (400 MHz, DMSO-d_6_) δ9.99(s, 1H, NH), 7.96(s, 1H, Ar-H), 7.58–7.44(t, 3H, Ar-H), 7.30(q, 2H, Ar-H), 7.04(t, 1H, Ar-H), 6.86(d, 2H, NH_2_), 6.75(s, 1H, Ar-H), 3.74 (s, 3H, OCH_3_), 3.4(s, 2H, SeCH_2_). ^13^CNMR (101 MHz, DMSO-d_6_) δ168, 167, 151, 141, 139, 138, 129, 123, 119, 117, 111, 109, 51, 32. MS (EI, 70 ev) *m*/*z* (%) = 364.20(M, 100.0, base peak), 230(40.48), 91.10(31), 77.05(24.13), 59(14.56).

### 2.3. The Biological Assays

The OSe candidates were prepared in DMSO stock solution (10 mM) and kept at −20 °C for further use.

#### 2.3.1. The Anticancer Activity

The anticancer activity of the OSe was performed using the MTT assay against liver (HepG2) and breast (MCF-7) carcinoma cells, as well as normal WI-38 cells following the reported method [14,15,16]. Experimental details can be found in the Supplementary Materials Section S1.

#### 2.3.2. The Antimicrobial Activity

According to the reported method, the OSe agents’ antimicrobial properties were estimated against *Staphylococcus aureus* (*S. aureus*), *Escherichia coli* (*E. coli*), and *Candida albicans* (*C. albicans*) microbial strains using the agar well diffusion assay [16,17]. In addition, the MICs (in M) were also recorded via the microdilution method following the reported procedure. Experimental details can be found in the Supplementary Materials Section S1 [16,18].

#### 2.3.3. The Antioxidant Activity

The DPPH and ABTS in vitro bioassays were used to assess the OSe antioxidant activities following the reported method [16,19,20,21]. Experimental details can be found in the Supplementary Materials Section S1.

## 3. Results and Discussion

### 3.1. Synthesis

Recently, OSe_2_ compounds have drawn much attention in pharmaceutical chemistry owing to their broad medicinal applications (e.g., antioxidant, chemopreventive, and anticancer activities) [22,23,24,25]. Unluckily, the synthesis of OSe_2_ compounds is limited by using toxic, expensive, and air-sensitive reagents (e.g., potassium selenocyanate) and commercially available selenium precursors. Furthermore, poor functional-group tolerance and sophisticated reaction conditions (e.g., under inert gas) are significant drawbacks of synthesizing the OSe_2_ compounds [15,26]. Accordingly, synthesizing new and stable OSe_2_ synthons is required to develop potential libraries for biological testing. Moreover, anthranilic acid derivatives manifested diverse pharmacological applications and were used to prepare different marketed drugs [11,12,27]. Therefore, combining the diselenide functionality into the backbone of anthranilic acid will enable access to unprecedented organic candidates designed to interfere with biotargets. The compound 2-amino-5-selenocyanatobenzoic acid (**3**) [13] drew our attention and was employed as a start to our synthetic approach. Compound **3** was synthesized by the selenocyanation of 2-aminobenzoic acid (**1**) [13]. However, our attempts to synthesize the diselenide functionality via hydrolysis or reduction of the selenocyanate group were unsuccessful, and we could not isolate any products. This is attributed to the zwitterionic nature of compound **3,** which arises from the presence of the carboxylic group in the ortho position to the amino group, which in turn causes difficulties in product isolation.

**Scheme 1:**
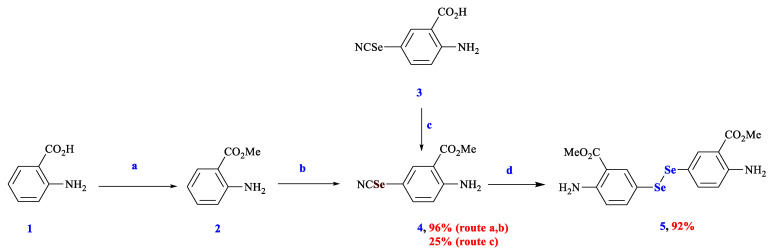
Preparation of compounds **2**, **4**, and **5**. Reagents and conditions: (a) anthranilic acid (**1**) (29.1 mmol), MeOH (60 mL), conc.H_2_SO_4_ (4 mL), reflux for 48 h; (b) SeO_2_ (30 mmol), malononitrile (15 mmol), DMSO (10 mL), methyl 2-aminobenzoate (12.5 mmol) (**2**); (c) 2-amino-5-selenocyanatobenzoic acid (**3**) (29.1 mmol), MeOH (60 mL), conc.H_2_SO_4_ (4 mL), reflux for 48 h; (d) methyl 2-amino-5-selenocyanatobenzoate (**4**) (4 mmol), NaOH (4 mmol), EtOH, room temperature (15 mL).

Furthermore, compound **3** is characterized by its low solubility in most organic solvents and high polarity limited its synthetic applications. Therefore, our synthetic strategy was oriented to mask the carboxylic or the amino group to overcome these difficulties. Accordingly, the alcoholic esterification of compound **3** furnished the respective methyl 2-amino-5-selenocyanatobenzoate (**4**); however, in low yield (25%). Therefore, we used an alternative synthetic approach to increase the yield starting from methyl 2-aminobenzoate (**2**) instead of 2-aminobenzoic acid (**1**). In this case, selenocyanation proceeded smoothly, and the yield was improved to 96% (Scheme 1). As a result, the methyl 2-amino-5-selenocyanatobenzoate (**4**) is new and features good solubility in most organic solvents and lower polarity compared to the corresponding 2-amino-5-selenocyanatobenzoic acid (**3**).

**Scheme 2:**
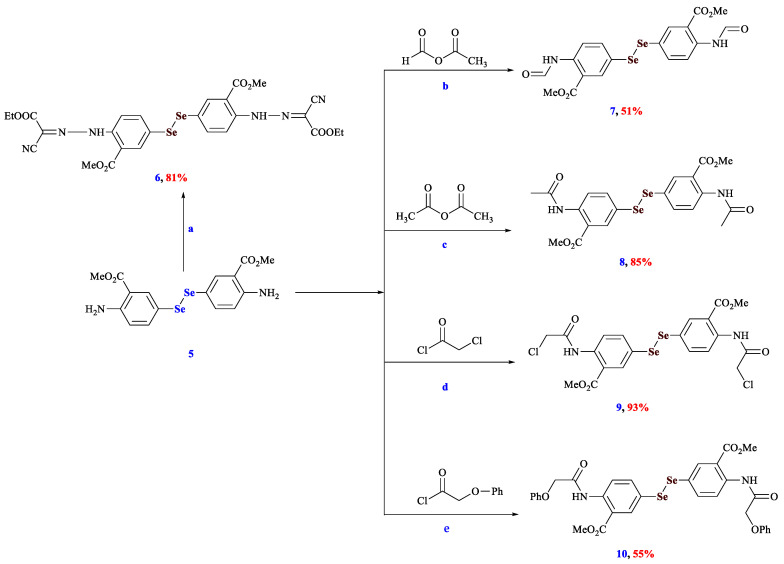
Synthesis of diselenides **6**, **7**, **8**, **9**, and **10**. Reagents and conditions: (a) compound **5** (2 mmol), HCl (3 mL), sodium nitrite (4.4 mmol), sodium acetate (2 g), H_2_O: EtOH (1:1), ethyl cyanoacetate (4.4 mmol); (b) THF (10 mL), acetic formic anhydride (36.3 mmol); (c) acetic anhydride (8 mL); (d) chloroacetyl chloride (4.4 mmol), K_2_CO_3_ (2 g), dry acetone (20 mL); (e) phenoxy acetyl chloride (2.2 mmol), dry ether (15 mL).

In addition, the hydrolysis of **4** using NaOH in EtOH afforded the corresponding diselenide **5** in 92% yield, thus, giving access to the symmetrical diselenide scaffolds known for their hepatoprotective activities (Scheme 1). The spectral data were used to identify the chemical structure of the diselenide **5**. Compound **5** exhibited a characteristic absorption band at 3455 cm^−1^ and 3344 cm^−1^ for NH_2_. The ^1^HNMR spectrum of compound **5** showed two singlet signals at δ7.00 ppm and δ3.74 ppm related to the proton of NH_2_ and OCH_3_, respectively. The MS spectrum of compound **5** showed molecular ion peaks at 460.15(M+H, 20.76) and the base peak at *m*/*z* 91. Similarly, bisdiazotization of the diselenide **5** and subsequent coupling with two equivalents of ethyl cyanoacetate afforded the corresponding disdiazo-based diselenide **6** in 81% yield (Scheme 2). Furthermore, the reaction of diselenide **5** with acetic formic anhydride, acetic anhydride, chloroacetyl chloride, and phenoxy acetyl chloride afforded the corresponding diselenide-based formamide **7**, acetanilide **8**, chloroacetamide **9**, and phenoxy acetamide **10** were obtained in 51%, 85%, 93%, and 55% yields, respectively (Scheme 2). Based on its spectrum data, the structure of compound **7** was established. The IR showed distinctive peaks at 3255 cm^−1^ for the NH and 1681 cm^−1^, and 1573 cm^−1^ for carbonyl groups. The ^1^HNMR spectrum of compound **8** showed three singlet signals at δ10.42 ppm for NH, at δ8.39 ppm related to the proton of the formyl group, and at δ3.75 ppm for OCH_3_. Its MS showed a molecular ion peak at 516.10(M, 25.78) and a base peak at *m*/*z* 198.

**Scheme 3:**
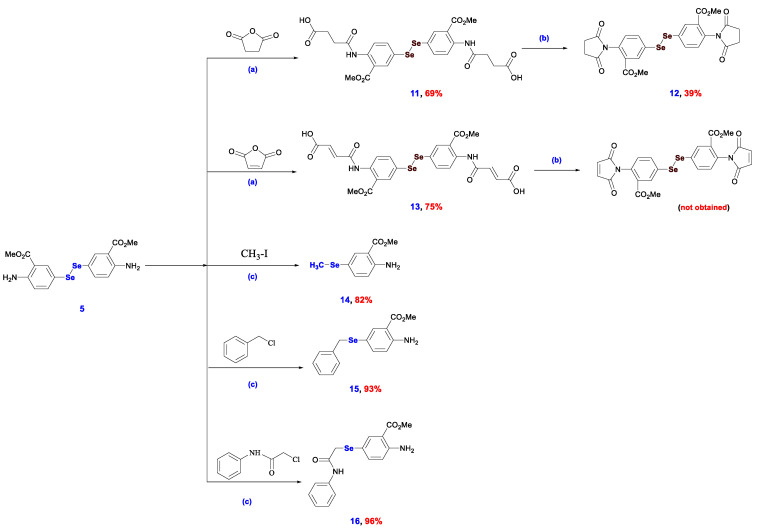
Synthesis of diselenides **11**, **12**, **13**, **14**, **15**, and **16**. Reagents and conditions: (a) Diselenide **5** (2 mmol), dry toluene (15 mL), maleic or succinic anhydride (4.4 mmol), room temperature; (b) respective amide-acids (0.5 mmol), acetic anhydride (5 mL), sodium acetate (250 mg); 50–60 °C; (c) diselenide **5** (2 mmol), halo derivatives (4.4 mmol), NaBH_4_ (6 mmol), EtOH (25 mL), room temperature.

Moreover, the reaction of the diselenide **5** with different succinic and maleic anhydrides afforded the corresponding diselenide-based succinanilic **11** and mealanilic acids **13** in 69% and 75% yields, respectively (Scheme 3). Additionally, warming the diselenide-based succinanilic **11** with acetic anhydride afforded the corresponding cyclic succinimide **12** via dehydration and subsequent cyclization, however, in low yield (39%) (Scheme 3). Unfortunately, our attempts to perform the same reaction with the diselenide-based mealanilic acid **13** were unsuccessful, and the starting material decomposition was often observed (Scheme 3).

Eventually, the one-pot alkaline reduction of the diselenide **5** began employing a mixture of NaBH_4_ and NaOH (1:1) in MeOH and subsequent reaction with alkyl halides, namely methyl iodide, benzyl chloride, and 2-chloro-N-phenylacetamide afforded the corresponding organoselenides **14**, **15**, and **16** in 82%, 93%, and 96% yields, respectively (Scheme 3). The IR spectra of compound **14** demonstrated characteristic absorption bands of NH_2_ at 3474 cm^−1^, 3363 cm^−1^, and 1686 cm^−1^ for the C=O. The ^1^HNMR spectrum of compound **14** exhibited singlet signals at δ6.75 ppm for NH_2_, singlet signal at δ3.62 ppm for OCH_3,_ and singlet signal at δ2.2 ppm for the SeCH_3_. The mass spectrum coincided with the predicted molecular mass for the proposed structure at *m*/*z* 245.10.

### 3.2. Biology

#### 3.2.1. Cytotoxicity of OSe_2_-tethered Anthranilic Acid Hybrids

OSe hybrids have recently gained much interest owing to their unprecedented cytoprotective and antioxidant activities [1,28,29,30]. We recently reported different OSe compounds with interesting antimicrobial, anticancer, and antioxidant activities [14,23,24,31,32]. We reported different tetrazole-based diselenides within this context with AChE inhibition and glutathione peroxidase-like activities [23]. Furthermore, we also developed diselenide-based urea with promising anti-HepG2 and apoptosis-induction properties [14]. On the other hand, we identified novel organoselenium-based pseudopeptides as promising highly effective chemo-sensitizers in treating HCC with cisplatin [26].

Accordingly, the anticancer properties of the OSe hybrids were assessed against two cancer cell lines, i.e., HepG2 and MCF-7 cells. Furthermore, their corresponding cytotoxicity was also estimated against the immortalized lung WI-38 fibroblasts employing the MTT assay. The Adriamycin cancer drug was used as the positive control. The concentration inhibition, 50%, needed to kill half of the cells (IC_50_) was estimated from (concentration-response plots) and tabulated in Table 1. Furthermore, the safety and selectivity of drugs are assessed from their respective therapeutic indices (TI), defined as the ratio between the IC_50_ exhibited by the compound against WI38 cells to the compound’s respective IC_50_ against cancer cells, i.e., HepG2 and MCF-7 cells (Table 1).

Interestingly, the cytotoxicity was more notable in the HepG2 cells compared to the MCF-7 cells. For instance, the OSe **14** was more cytotoxic (IC_50_ = 3.57 ± 0.1 µM) than the standard anticancer drug Adriamycin (IC_50_ = 4.50 ± 0.2 µM). Furthermore, OSe **5** and **15** showed interesting anti-HepG2 cytotoxic patterns with IC_50_ = 7.08 ± 0.6 µM and 6.48 ± 0.4 µM, respectively (Table 1). Moreover, moderate cytotoxicity was observed in the case of OSe **4** and **6** with IC_50_ = 11.21 ± 1.0 µM and 16.22 ± 1.2 µM, respectively.

In the case of MCF-7 cells, the OSe **14** and **5** showed comparable cytotoxicity (IC_50_ = 5.64 ± 0.3 µM and 9.89 ± 0.7 µM) to Adriamycin (IC_50_ = 4.17 ± 0.2 µM), whereas moderate cytotoxicity was noted by the OSe **15** and **6** with IC_50_ = 12.76 ± 0.9 µM and 15.92 ± 1.2 µM (Table 1).

Drugs with high TI values kill cancer cells, leaving normal cells unaffected. Therefore, these drugs are highly preferable in chemotherapy, and the high TI values point to the potential selectivity and safety of a specific drug candidate [18,33,34,35,36]. Within this context, better TI values were noticed in the case of HepG2 cells compared to the MCF-7 cells. The best selective cytotoxicity patterns for HepG2 cells were observed in the case of OSe compounds **14** and **5** with TI values of 17 and 5. On the other hand, OSe compounds **14**, **5**, and **6** showed modest selective cytotoxicity patterns for MCF-7 cells with TI values of 11, 3.6, and 2.7 (Table 1). Eventually, such interesting selective anticancer activity merits more investigations using a broader panel of normal and cancerous cells and in vivo experiments.

#### 3.2.2. Evaluation of the Antimicrobial Activities of the OSe Compounds

The promising anticancer activities manifested by the OSe compounds encourage us to further evaluate their respective antimicrobial activity against a panel of gram-negative bacteria (e.g., *E. coli*) and the gram-positive bacteria (e.g., *S. aureus*) as well as a fungal strain (*C. albicans*) using the method of agar diffusion. The clotrimazole antifungal and the ampicillin antibacterial drugs were used as the positive controls. The zones of inhibition diameters (ZID) (in mm) and the activity index percentage (IA%) are shown in Table 2.

In general, *C. Albicans* fungus and *S. aureus* gram-positive bacteria were more sensitive than gram-negative bacteria toward the OSe compounds. Interestingly, OSe **14** showed similar antifungal activity against *C. albicans* to the standard drug clotrimazole (IA% = 100%) and exciting antibacterial activity against *E. coli* (IA% = 91.3%) and *S. aureus* (IA% = 90.5%). Accordingly, the minimum inhibitory concentration (MIC) of **14** was also estimated (Table 3) to confirm its antimicrobial activity. In this regard, **14** manifested similar antimicrobial activity to the standard drugs clotrimazole and ampicillin drugs with MIC = of 2, 2, and 0.5 μM against *C. albicans*, *S. aureus*, and *E. coli* strains, respectively.

Similarly, OSe compounds **15** and **5** have shown excellent antimicrobial activities with A% of 91.7% and 87.5% against *C. albicans,* 85.7% and 85.7% against *S. aureus*, and 73.9% and 82.6% against *E. coli.* Additionally, good antimicrobial activities were also observed in the case of OSe compounds **6** and **4,** with IA% of 70.8% and 75% against *C. albicans,* 76.2% and 76.2% against *S. aureus*, and 69.6% and 60.9% against *E. coli* (Table 2). Ultimately, such interesting antimicrobial patterns are worth further research and screening against an extensive panel of bacterial and fungal strains.

#### 3.2.3. The Antioxidant Properties of the OSe Compounds

**Figure 2:**
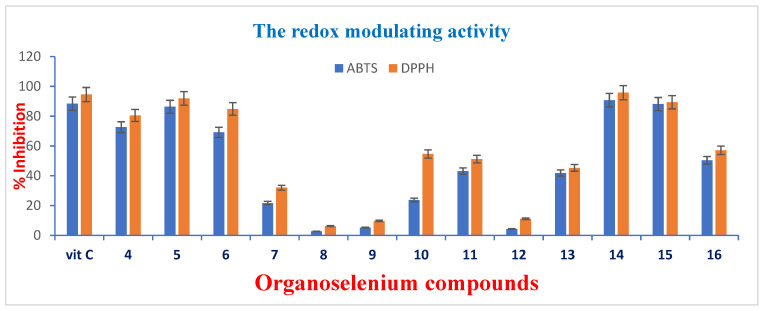
The OSe compounds redox activities assessment using the ABTS and DPPH experiments. All tests are mean ± SD. ABTS experiment: absorbance was followed at 734 nm for OSe compounds (50 µL, 1 mM in MeOH) and ABTS solution (60 µL). DPPH experiment: Absorbance was monitored after 30 min at 517 nm for OSe compounds (200 µL, 1 mM in MeOH) and DPPH (400 µL).

The redox modulation activities of the OSe compounds were extensively explored over the last decade owing to their chemopreventive and antioxidant potency [7,37]. The latter is usually investigated by the ABTS and DPPH in vitro tests employing vitamin C as the positive control [21,38]. The OSe compound’s magnitude estimated the antioxidant potential to decolorize the distinctive colors of the DPPH**^.^** and ABTS**^.^** radicals at 517 nm and 734 nm, respectively (Figure 2).

As displayed in Figure 2, OSe compounds **14**, **5**, **15**, **6**, and **4** exhibited 96%, 92%, 89%, 85%, and 81% scavenging activities compared to 95% by vitamin C in the DPPH and assay, respectively. Similarly, OSe compounds **14**, **15**, **5**, **4**, and **6** exhibited 91%, 88%, 86%, 73%, and 69% scavenging activities compared to 88% by vitamin C in the DPPH and assay, respectively. These results encourage us to further determine the lowest dose needed to decrease the absorbance by 50% in the ABTS and DPPH assays for the most exciting and active OSe compound **14** (Table 4). To conclude, OSe compounds **4**, **6**, **5**, **14**, and **15** exhibited promising antioxidant activity, and these results are in good accordance with the antimicrobial and anticancer effects, ensuring these compounds’ potential biological activities.

## 4. Conclusions

Sixteen new diselenide-tethered methyl anthranilate hybrids were synthesized in excellent yields (up to 96%), and their chemical structures were elucidated using different spectroscopic techniques. Their antimicrobial and antitumor activities were assessed against various microbial strains and cancer cells. The OSe **14** showed the best antifungal properties with action similar to the standard drug clotrimazole (IA% = 100%) and manifested promising antibacterial activity against *E. coli* (IA% = 91.3%) and *S. aureus* (IA% = 90.5%). Furthermore, OSe compounds **4**, **5**, **6**, and **15** have also shown good antimicrobial activities. Similarly, the OSe **14** was the most cytotoxic (IC_50_ = 3.57 ± 0.1 µM) against the HepG2 and even more than the Adriamycin drug (IC_50_ = 4.50 ± 0.2 µM) and showed TI of 17, pointing to selective potential and safety. In the case of MCF-7 cells, the OSe **14** and **5** showed comparable cytotoxicity (IC_50_ = 5.64 ± 0.3 µM and 9.89 ± 0.7 µM) to Adriamycin (IC_50_ = 4.17 ± 0.2 µM), whereas moderate cytotoxicity was noted by the OSe **15** and **6** with IC_50_ = 12.76 ± 0.9 µM and 15.92 ± 1.2 µM (Table 1).

Furthermore, OSe compounds **14** and **5** exhibited promising antioxidants with 96%, 92%, 91%, and 86% scavenging activities compared to 95% by vitamin C in the DPPH and ABTS assays, respectively. To this end, these results point to potential antimicrobial, anticancer, and antioxidant activities of the OSe **14** and warrant further studies.

## Figures and Tables

**Table 1:** The antiproliferative activities of the OSe compounds.

Compounds	MCF7 ^a^		HepG2 ^a^		WI38 ^a^
	IC_50_ (µM) ^a^	TI ^c^	IC_50_ (µM) ^a^	TI ^c^	IC_50_ (µM) ^a^
**Adriamycin**	4.17 ± 0.2	1.6	4.50 ± 0.2	1.5	6.72 ± 0.5
**4**	23.45 ± 1.8	2.3	11.21 ± 1.0	4.8	52.62 ± 3.1
**5**	9.89 ± 0.7	3.6	7.08 ± 0.6	5	35.91 ± 2.3
**6**	15.92 ± 1.2	2.7	16.22 ± 1.2	2.6	42.45 ± 2.6
**7**	79.01 ± 4.2	-	83.67 ± 4.4	-	45.73 ± 2.7
**8**	- ^b^	-	- ^b^	-	- ^b^
**9**	- ^b^	-	- ^b^	-	- ^b^
**10**	53.49 ± 3.0	-	81.48 ± 4.2	-	38.23 ± 2.5
**11**	55.10 ± 3.2	-	43.70 ± 2.6	-	- ^b^
**12**	- ^b^	-	- ^b^	-	- ^b^
**13**	76.24 ± 4.0	-	78.53 ± 3.9	-	47.03 ± 2.8
**14**	5.64 ± 0.3	11	3.57 ± 0.1	17	62.38 ± 3.6
**15**	12.76 ± 0.9	2	6.48 ± 0.4	3.9	25.81 ± 1.8
**16**	50.81 ± 2.9	1.4	30.62 ± 2.4	2.3	71.90 ± 4.1

^a^ The MTT bioassay was used to estimate the antitumor properties. MCF-7, HepG2, and WI-38 cells were incubated for 24 h with serial concentrations of the OSe compounds. ^b^ No proliferation inhibition was observed (IC_50_ ˃ 100 µM); ^c^ TI is defined as the ratio between the IC_50_ exhibited by the compound against WI38 cells to the compound’s respective IC_50_ against HepG2 and MCF-7 cells.

**Table 2:** The antimicrobial properties of the OSe compounds.

Compound	*E. coli*		*S. aureus*		*C. Albicans*	
	ZID (mm) ^a^	IA% ^b^	ZID (mm) ^a^	IA% ^b^	ZID (mm) ^a^	IA% ^b^
**4**	14	60.9	16	76.2	18	75.0
**5**	19	82.6	18	85.7	21	87.5
**6**	16	69.6	16	76.2	17	70.8
**7**	-	-	3	14.3	4	16.7
**8**	-	-	-	-	-	-
**9**	-	-	-	-	-	-
**10**	5	21.7	8	38.1	5	20.8
**11**	5	21.7	7	33.3	10	41.7
**12**	-	-	-	-	-	-
**13**	-	-	4	19.0	5	20.8
**14**	21	91.3	19	90.5	24	100
**15**	17	73.9	18	85.7	22	91.7
**16**	6	26.1	9	42.8	13	54.2
**Ampicillin**	23	100	21	100	-	-
**Clotrimazole**	-	-	-	-	24	100

^a^ Inhibition zones (ZID) are expressed in diameters (mm) employing disks (6 mm) soaked with 20 μM of the OSe agents. ^b^ Activity index (IA%) percentage is the ratio of the inhibition zone of the OSe compound to the inhibition zone of the positive control.

**Table 3:** The MIC of OSe compound **14**.

Compounds	MIC (μM)		
	*E. coli*	*S. aureus*	*C. Albicans*
**14**	0.5	2	2
**Ampicillin**	0.5	1	-
**Clotrimazole**	-	-	2

**Table 4:** The OSe compounds **14** redox properties.

Compounds	IC_50_ (µM)	
	DPPH	ABTS
**Vitamin C**	19.18 ± 0.13	28.16 ± 0.19
**14**	18.67 ± 0.12	29.14 ± 0.20

## Data Availability

Not applicable.

## Supplementary Materials

The following supporting information can be downloaded at: https://www.mdpi.com/article/10.3390/biom12121765/s1, Section S1: The biological assays; Section S2: Copies of ^1^H & ^13^CNMR spectra IR and MS.
