# Supplementary material for: Anticancer, Antimicrobial, and Antioxidant Activities of Organodiselenide-Tethered Methyl Anthranilates
Source: Biomolecules. 2022 Nov 27;12(12):1765. doi: 10.3390/biom12121765 (PMC9775310; doi:10.3390/biom12121765)
Supplement: Supplementary file 1 [file biomolecules-12-01765-s001.zip › biomolecules-2001625-supplementary.pdf]

Anticancer, Antimicrobial, and Antioxidant Activities of Organodiselenide-Tethered Methyl Anthranilates

Batool Al-Abdallah [1], Yasair S. Al-Faiyz [1] and Saad Shaaban [1,2,*]

[1] Chemistry Department, College of Science, King Faisal University, Al-Ahsa 31982, Saudi Arabia
[2] Chemistry Department, Faculty of Science, Mansoura University, Mansoura 35516, Egypt
* Correspondence: sibrahim@kfu.edu.sa or dr_saad_chem@mans.edu.sa

SUPPORTING INFORMATION	PAGE
The biological assays	S2
Copies of ^{1}H & ^{13}CNMR spectra IR and MS	S6

1.1. The biological assays

1.1.1. The anticancer activity

The breast adenocarcinoma (MCF-7), Hepatocellular carcinoma (HEPG-2), and normal fibroblast (WI-38) cells were obtained from ATCC via Holding company for biological products and vaccines (VACSERA), Cairo, Egypt. The reagents RPMI-540 medium, MTT, DMSO, and fetal bovine serum was obtained from Sigma Aldrich. Dulbecco's modified Eagle's medium was used as the culture medium for the cells, and the cytotoxicity was evaluated using the reported MTT assay. Doxorubicin was used as the positive reference, and the IC_{50} values were obtained from the corresponding dose-response curve.

MTT assay[1-3]

This colorimetric assay is based on converting the yellow tetrazolium bromide (MTT) to a purple formazan derivative by mitochondrial succinate dehydrogenase in viable cells. Cell lines were cultured in RPMI-540 medium with 10% fetal bovine serum. Antibiotics added were 100 units/ml penicillin and 100µg/ml streptomycin at 37 C in a 5% Co_2 incubator. The cell lines were seeded in a 96-well plate at a density of $1.0 x 10^4$ cells/well. at 37 C for 48 h under 5% Co_2. After incubation, the cells were treated with different concentrations of compounds and incubated for 13 h. After 13 h of drug treatment, 9 µl of MTT solution at 5mg/ml was added and incubated for 4 h. Finally, dimethyl sulfoxide (DMSO) in the volume of 100 µl is added to each well to dissolve the purple formazan formed. The colorimetric assay is measured and recorded at an absorbance of 570 nm using a plate reader (EXL 800, USA). The percentage of the relative cell viability was calculated as (A570 of treated samples/A570 of the untreated sample) X 100.

1.1.2. The antimicrobial activity

The antimicrobial activities of the OSe compounds were evaluated against *C. albicans* yeast as well as *E. coli* gram-negative and *S. aureus* gram-positive bacteria employing the agar well diffusion assay [4].

Briefly, a concentration of 1 mM was prepared for each compound by dissolving in DMSO. Paper discs of standard size (5cm) were sterilized in an autoclave and soaked in 9 μL of the test compounds, and placed in the Petri dishes, which in turn contain a nutrient media (agar 9 g, peptone 5 g, and beef extract 3 g) seeded with the dedicated strain. Incubation lasted for 13 h at 36 ^{0}C. Experiments were replicated three times and the antifungal clotrimazole and antibiotic ampicillin were used as standards. The % activity index for the complex was determined and depicted in table 2.

Furthermore, the MICs (in μM) were determined by the microdilution method according to the reported protocol.

1.1.3. The antioxidant activity

1.1.3.1. The DPPH bioassay

The hydrogen atom or electron donation ability of the corresponding compounds was measured by estimating the bleaching of the purple color of a methanolic solution of DPPH [5]. This spectrophotometric assay uses stable DPPH$^{\cdot}$ reagent. The sample was prepared by adding 90 mL of the OSe compounds (1 mM in methanol) to 400 mL DPPH in methanol. After 30 min of incubation in the dark, the absorbance was read against a blank at 56 nm. Ascorbic acid (vitamin C) and ebselen were used as standard antioxidants (positive control). A blank sample was run without DPPH. A negative control sample was run using methanol instead of the sample. The radical scavenging activity was calculated

using the following equation:

$$\text{Inhibition\%} = (A_{blank} - A_{sample})/(A_{blank})*100.$$

1.1.3.2. The ABTS bioassay

The antioxidant activity of the investigated compounds was assessed using 2,2'-azino-bis(3-ethylbenzothiazoline-6-sulphonic acid (ABTS) method [6]. The radical cation derived from ABTS was prepared by the reaction of 60 mM ABTS solution with 0.3 M manganese dioxide solution in 0.1M

phosphate buffer, pH 7. Then, the mixture was shaken, centrifuged, filtered, and the absorbance ($A_{control}$) of the resulting green-blue solution (ABTS radical solution) was measured at wavelength 734 nm. Then, 50 mL of 1 mg/ml test compound in phosphate-buffered methanol was added. The absorbance (A_{test}) was measured. The reduction in color intensity was expressed as % inhibition. The % inhibition for each compound is calculated from the following equation

$$\text{Inhibition\%} = (A_{control}-A_{sample})/(A_{control})*100$$

Ascorbic acid (vitamin C) was used as a standard antioxidant (positive control). A blank sample was run without ABTS and using MeOH/phosphate buffer (1:1) instead of the sample. A negative control sample was run with MeOH/phosphate buffer (1:1) instead of a tested compound.

*Methyl 2-amino-5-selenocyanatobnzoate. (**4**).*

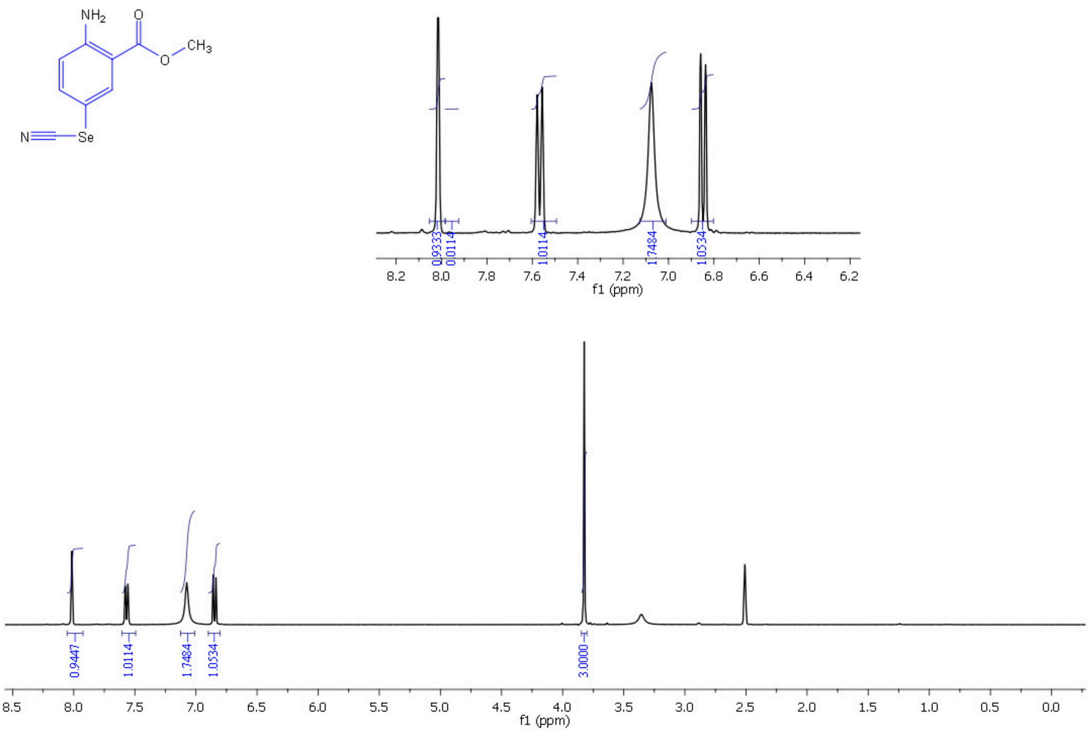

Figure S1. ^{1}HNMR chart of compound **4**

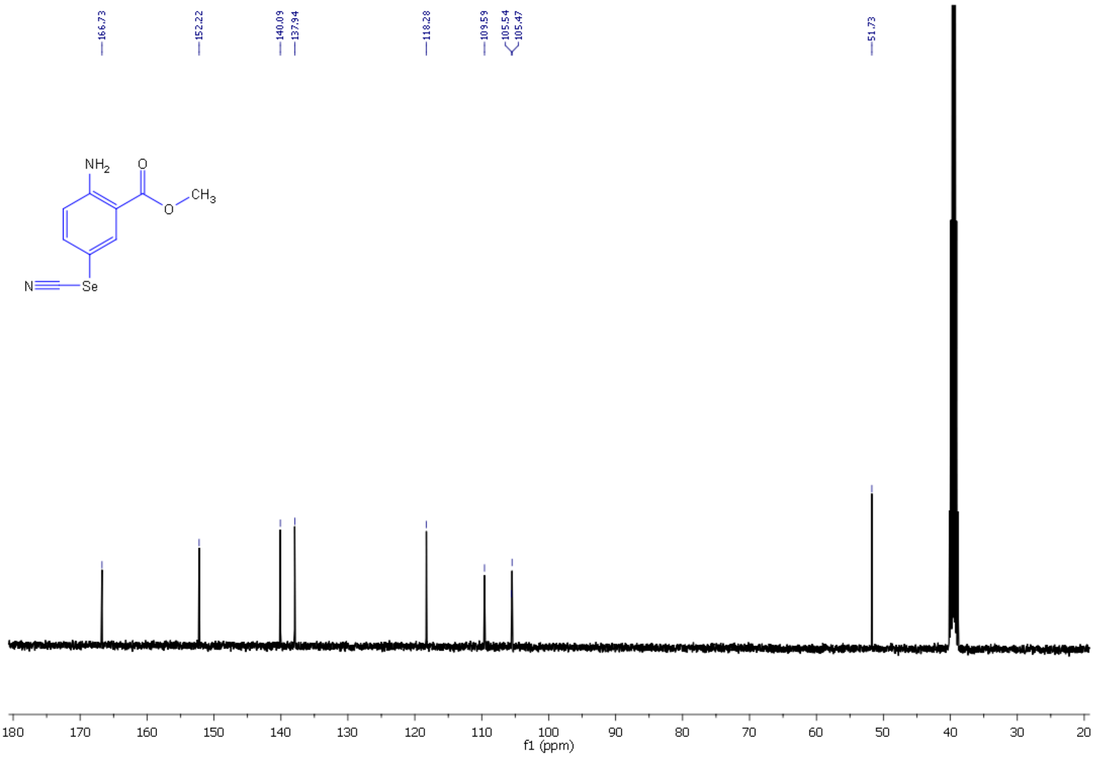

Figure S2. ^{13}CNMR chart of compound **4**

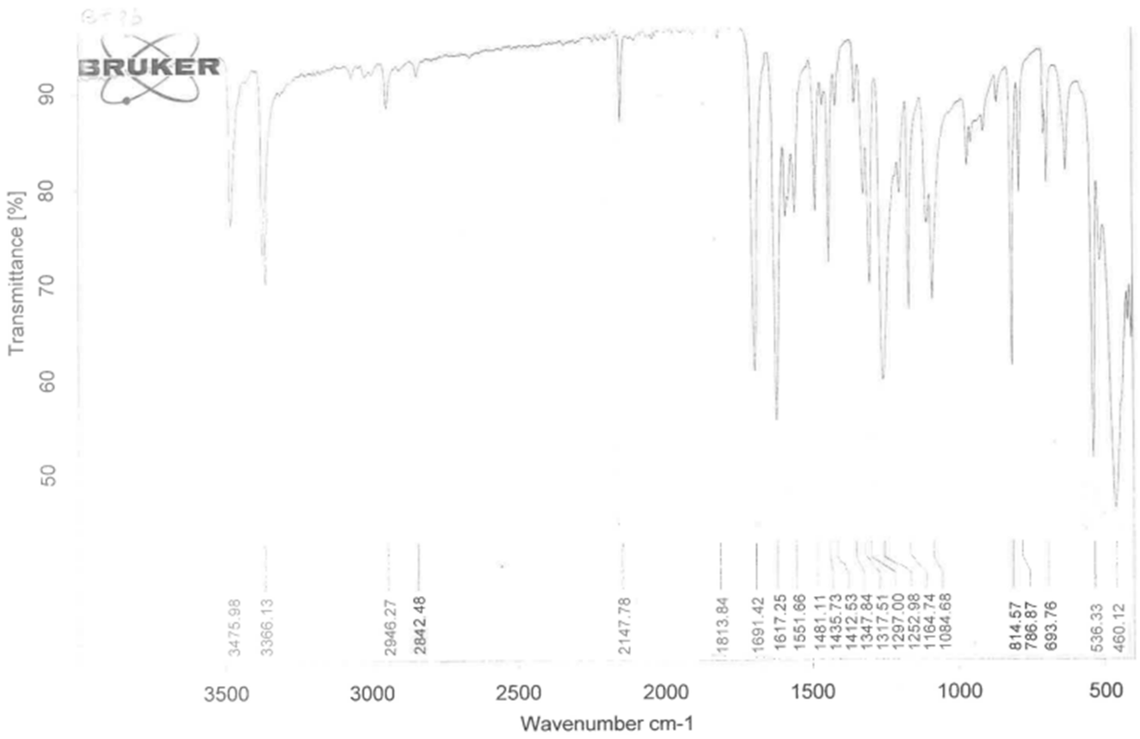

Figure S3. IR chart of compound **4**

06-Jan-07 19:37:16

Cairo University
Micro Analytical Center

DI Analysis
Shimadzu Qp-2010 Plus

Sample Information

Analyzed by	: Dr. Mai Younis
Analyzed	: 06/01/2007 07:26:19 م
Sample Name	: B2
Sample ID	:
Customer Name	: Dr.Mohamed Soliman - Science - Cairo
Data File	: C:\GCMSsolution\Data\Project1\B2.QGD
Org Data File	: C:\GCMSsolution\Data\Project1\B2.QGD
Method File	: C:\GCMSsolution\Data\Project1\High Temperature Op
Org Method File	: C:\GCMSsolution\Data\Project1\High Temperature Op
Report File	:
Tuning File	: C:\GCMSsolution\System\Tune1_default.qgt
$End1f$Modified by	: Dr. Mai Younis
Modified	: 06/01/2007 07:32:04 م

Method

====== Analytical Line 1 ======

IonSourceTemp	:250.00 °C
[MS Table]	
--Group 1 - Event 1--	
Start Time	:0.00min
End Time	:10.00min
ACQ Mode	:Scan
Event Time	:0.50sec
Scan Speed	:1000
Start m/z	:50.00
End m/z	:510.00
Electron Voltage	: 70 eV
Ionization Mode	: EI

C:\GCMSsolution\Data\Project1\B2.QGD

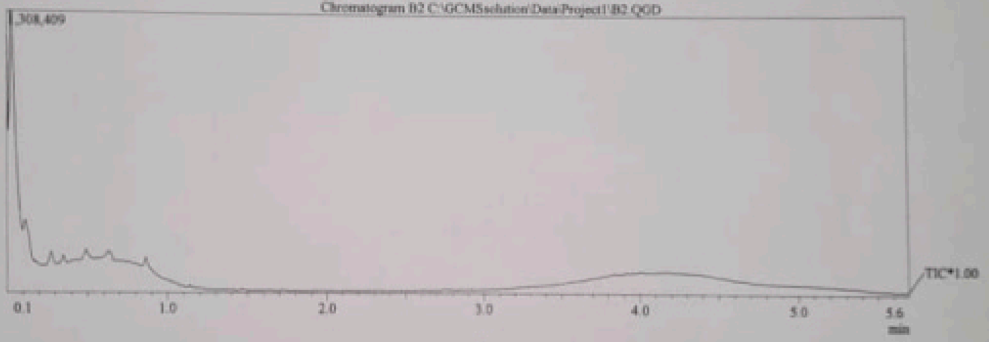

Spectrum

Line#:1 R.Time:4.1(Scan#:490)
MassPeaks:76
RawMode:Single 4.1(490) BasePeak:59(21586)
BG Mode:None Group 1 - Event 1

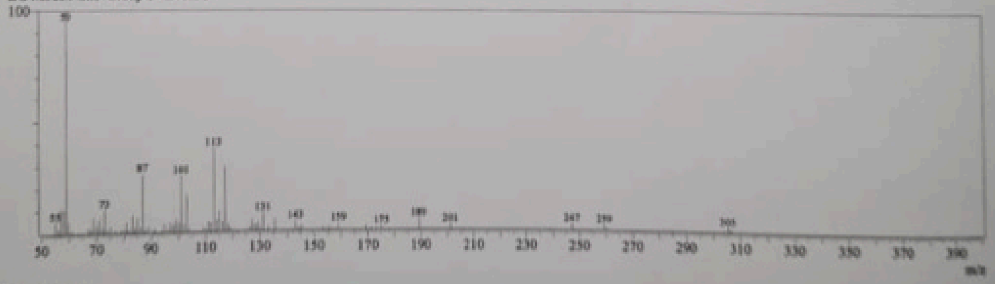

Mass Table
Line#:1 R.Time:4.1(Scan#:490)
MassPeaks:76
RawMode:Single 4.1(490) BasePeak:59(21586)
BG Mode:None Group 1 - Event 1

#	m/z	Abs. In	Rel. Int.	#	m/z	Abs. In	Rel. Int.	#	m/z	Abs. In	Rel. Int.
1	55.05	1062	4.92	4	58.05	2312	10.71	7	61.00	236	1.09
2	56.05	354	1.64	5	59.00	21586	100.00	8	67.10	468	2.17
3	57.05	2418	11.20	6	59.95	1083	5.02	9	68.15	341	1.58

1 / 2

Figure S4. Mass chart of compound **4**

#	m/z	Abs. In	Rel. Int.
10	69.05	1515	7.02
11	70.10	633	2.93
12	71.05	1365	6.32
13	72.05	440	2.04
14	73.00	2258	10.46
15	74.00	302	1.40
16	75.05	583	2.70
17	77.00	268	1.24
18	79.00	223	1.03
19	80.10	324	1.50
20	81.10	954	4.42
21	82.15	315	1.46
22	83.10	1628	7.54
23	84.10	481	2.23
24	85.05	1397	6.47
25	86.05	492	2.28
26	87.05	5752	26.65
27	88.00	314	1.45
28	89.05	560	2.59
29	91.10	327	1.51
30	94.10	202	0.94
31	95.10	807	3.74
32	96.10	278	1.29

#	m/z	Abs. In	Rel. Int.
33	97.10	945	4.38
34	98.15	599	2.77
35	99.10	1268	5.87
36	100.15	840	3.89
37	101.10	5503	25.49
38	102.15	699	3.24
39	103.10	3649	16.90
40	104.10	322	1.49
41	109.10	401	1.86
42	110.10	220	1.02
43	111.10	969	4.49
44	112.15	807	3.74
45	113.10	8084	37.45
46	114.10	1199	5.55
47	115.10	2001	9.27
48	116.25	936	4.34
49	117.15	6264	29.02
50	118.15	607	2.81
51	119.20	271	1.26
52	126.15	310	1.44
53	127.10	1102	5.11
54	128.15	589	2.73
55	129.10	740	3.43

#	m/z	Abs. In	Rel. Int.
56	130.15	304	1.41
57	131.10	1722	7.98
58	133.15	432	2.00
59	135.20	1132	5.24
60	143.10	908	4.21
61	144.10	210	0.97
62	145.20	492	2.28
63	153.20	234	1.08
64	155.20	281	1.30
65	159.15	662	3.07
66	169.20	334	1.55
67	171.20	204	0.95
68	173.20	202	0.94
69	175.20	346	1.60
70	189.20	1038	4.81
71	201.15	502	2.33
72	247.20	575	2.66
73	259.35	515	2.39
74	260.40	207	0.96
75	305.40	401	1.86
76	306.40	281	1.30

2/2

Mass chart of compound **4**

Dimethyl 5,5'-diselanediylbis(2-aminobenzoate) (***5***)

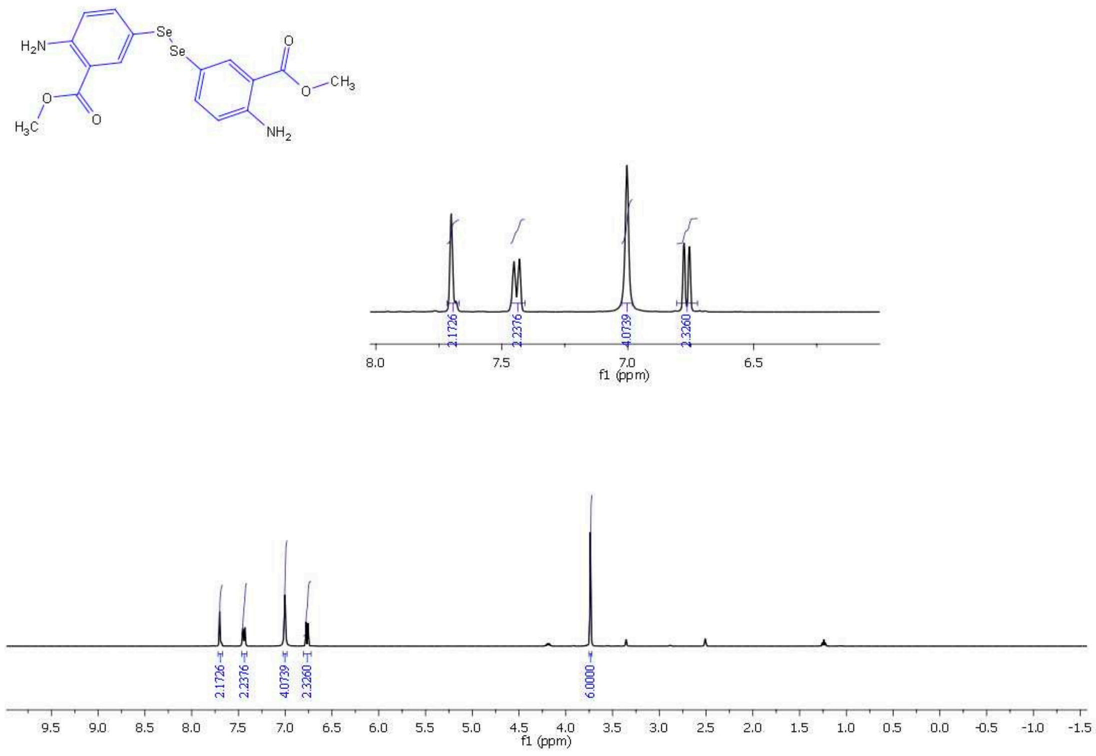

Figure S5. ^{1}HNMR chart of compound **5**

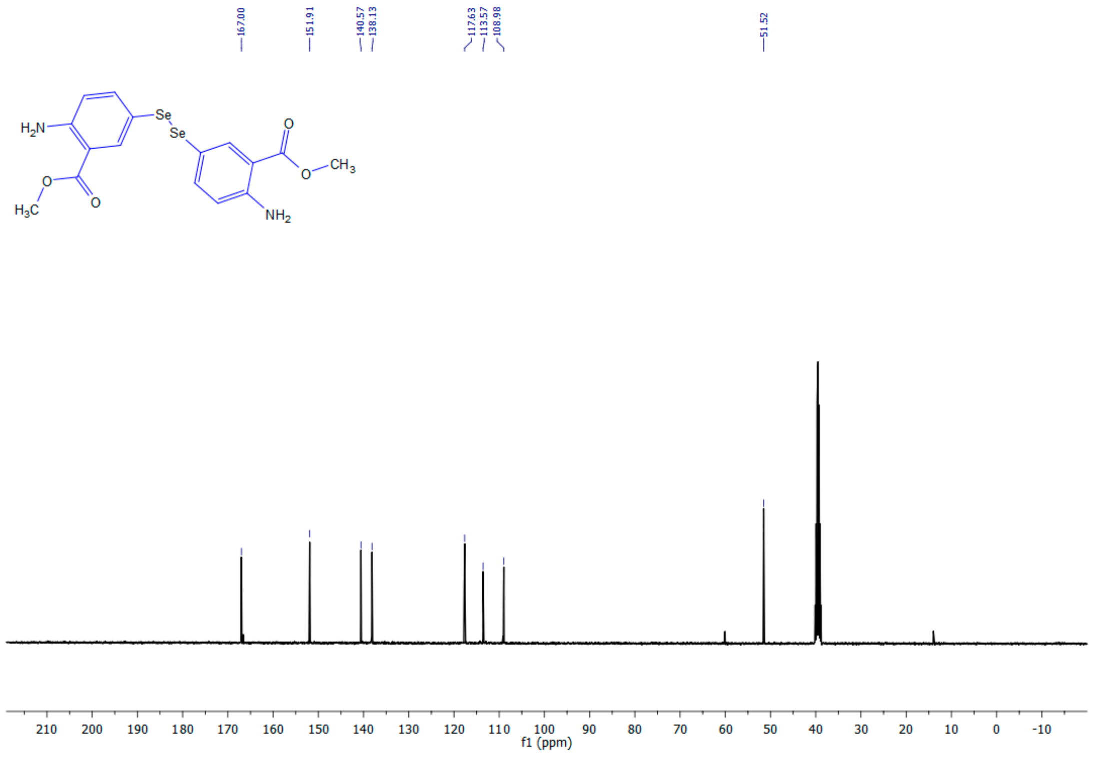

Figure S6. ^{13}CNMR chart of compound **5**

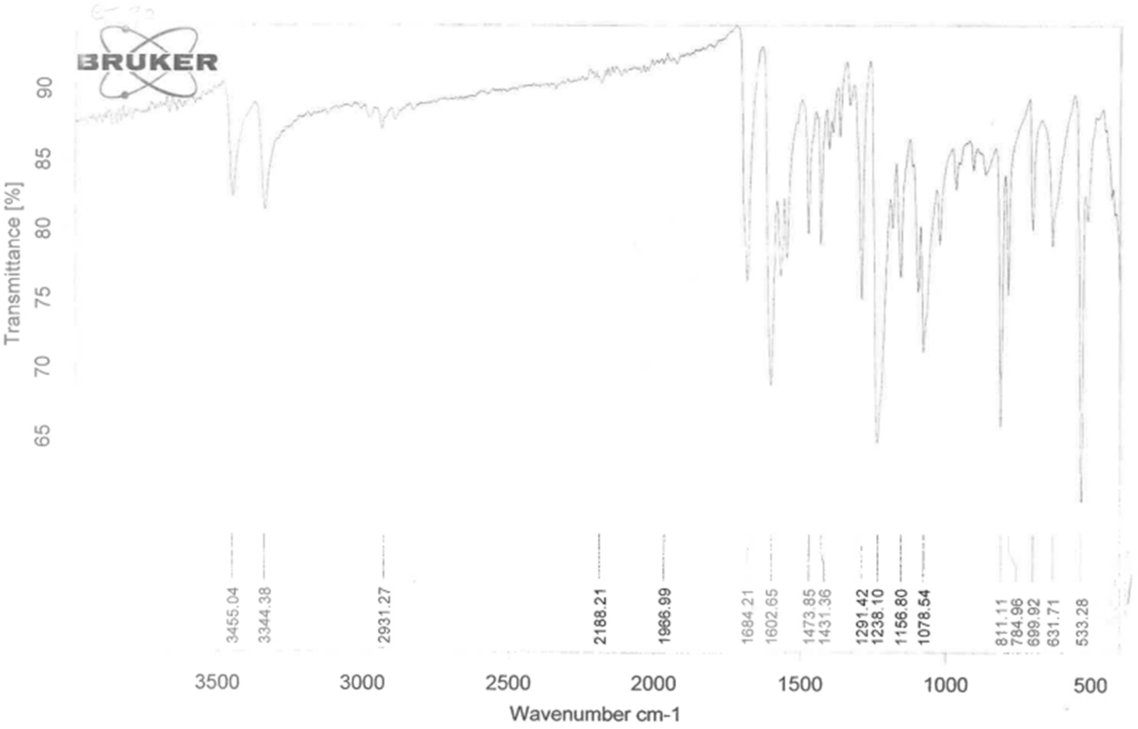

Figure S7. IR chart of compound **5**

06-Jan-07 19:30:32

Cairo University
Micro Analytical Center

DI Analysis
Shimadzu Qp-2010 Plus

Sample Information

Analyzed by	: Dr. Mai Younis
Analyzed	: 06/01/2007 07:17:06 م
Sample Name	: B1
Sample ID	:
Customer Name	: Dr.Mohamed Soliman - Science - Cairo
Data File	: C:\GCMSsolution\Data\Project1\B1.QGD
Org Data File	: C:\GCMSsolution\Data\Project1\B1.QGD
Method File	: C:\GCMSsolution\Data\Project1\High Temperature Op
Org Method File	: C:\GCMSsolution\Data\Project1\High Temperature Op
Report File	:
Tuning File	: C:\GCMSsolution\System\Tune1_default.qgt
$End1f$Modified by	: Dr. Mai Younis
Modified	: 06/01/2007 07:22:24 م

Method

——— Analytical Line 1 ———

IonSourceTemp	:250.00 °C
[MS Table]	
--Group 1 - Event 1--	
Start Time	:0.00min
End Time	:10.00min
ACQ Mode	:Scan
Event Time	:0.50sec
Scan Speed	:1000
Start m/z	:50.00
End m/z	:510.00
Electron Voltage	: 70 eV
Ionization Mode	: EI

C:\GCMSsolution\Data\Project1\B1.QGD

Chromatogram B1 C:\GCMSsolution\Data\Project1\B1.QGD

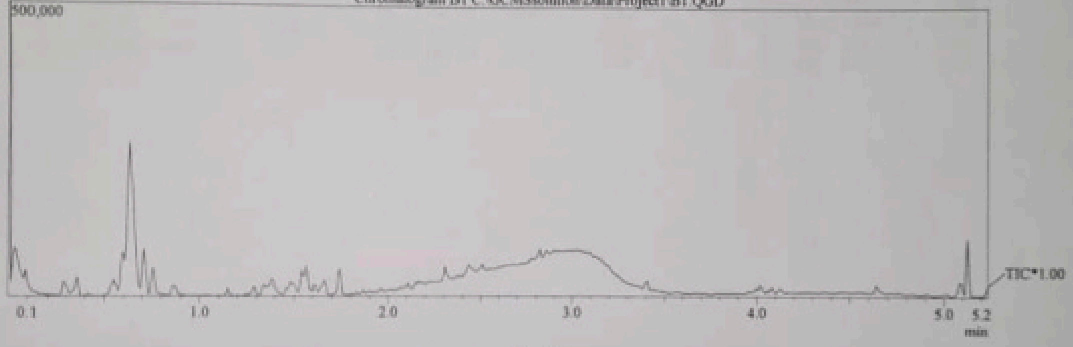

Spectrum

Line#:1 R.Time:2.9(Scan#:352)
MassPeaks:90
RawMode:Single 2.9(352) BasePeak:91(10811)
BG Mode:None Group 1 - Event 1

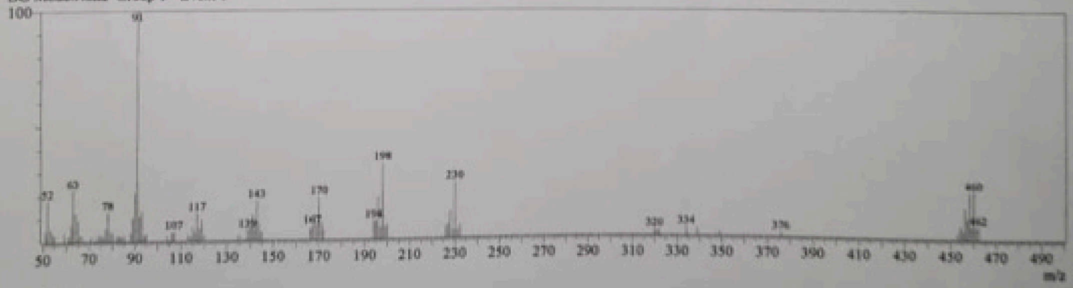

Mass Table
Line#:1 R.Time:2.9(Scan#:352)
MassPeaks:90
RawMode:Single 2.9(352) BasePeak:91(10811)
BG Mode:None Group 1 - Event 1

#	m/z	Abs. In	Rel. Int.	#	m/z	Abs. In	Rel. Int.	#	m/z	Abs. In	Rel. Int.
1	51.05	519	4.80	4	54.05	471	4.36	7	61.00	255	2.36
2	51.95	1953	18.06	5	55.10	305	2.82	8	62.05	723	6.69
3	53.05	620	5.73	6	59.05	411	3.80	9	63.00	2398	22.18

1 / 2

Figure S8. Mass chart of compound **5**

#	m/z	Abs. In	Rel. Int.
10	64.05	1311	12.13
11	65.05	960	8.88
12	66.00	278	2.57
13	67.00	247	2.28
14	71.00	202	1.87
15	74.00	313	2.90
16	75.00	255	2.36
17	76.00	214	1.98
18	77.05	571	5.28
19	78.05	1366	12.64
20	79.10	466	4.31
21	80.10	388	3.59
22	82.10	233	2.16
23	83.10	260	2.40
24	84.10	220	2.03
25	85.10	278	2.57
26	88.05	377	3.49
27	89.05	1081	10.00
28	90.05	2287	21.15
29	91.05	10811	100.00
30	92.05	1150	10.64
31	92.95	1429	13.22
32	94.00	254	2.35
33	95.00	354	3.27
34	104.00	249	2.30
35	106.10	398	3.68
36	107.00	444	4.11

#	m/z	Abs. In	Rel. Int.
37	113.00	308	2.85
38	113.90	222	2.05
39	114.90	636	5.88
40	116.05	385	3.56
41	117.00	1319	12.20
42	118.05	599	5.54
43	119.10	1022	9.45
44	120.10	310	2.87
45	135.10	289	2.67
46	139.00	482	4.46
47	140.00	614	5.68
48	141.05	1196	11.06
49	142.05	619	5.73
50	143.00	1894	17.52
51	144.00	850	7.86
52	144.95	409	3.78
53	166.05	493	4.56
54	167.05	639	5.91
55	168.05	1148	10.62
56	169.15	688	6.36
57	170.05	2031	18.79
58	171.10	802	7.42
59	172.10	420	3.88
60	194.05	863	7.98
61	195.05	858	7.94
62	196.00	1993	18.43
63	197.05	575	5.32

#	m/z	Abs. In	Rel. Int.
64	198.00	3602	33.32
65	199.00	591	5.47
66	200.05	690	6.38
67	226.05	505	4.67
68	227.05	655	6.06
69	228.00	1222	11.30
70	229.15	316	2.92
71	230.05	2640	24.42
72	231.05	347	3.21
73	232.05	594	5.49
74	320.00	290	2.68
75	321.00	222	2.05
76	322.00	228	2.11
77	334.00	398	3.68
78	339.00	318	2.94
79	349.00	230	2.13
80	376.00	215	1.99
81	453.00	263	2.43
82	454.20	604	5.59
83	455.20	425	3.93
84	456.25	1479	13.68
85	457.20	1015	9.39
86	458.20	2169	20.06
87	459.15	562	5.20
88	460.15	2244	20.76
89	461.15	521	4.82
90	462.10	618	5.72

Mass chart of compound **5**

Dimethyl 5,5'-diselanediylbis(2-(2-(1-cyano-2-ethoxy-2-oxoethylidene) hydrazinyl) benzoate) (***6***).

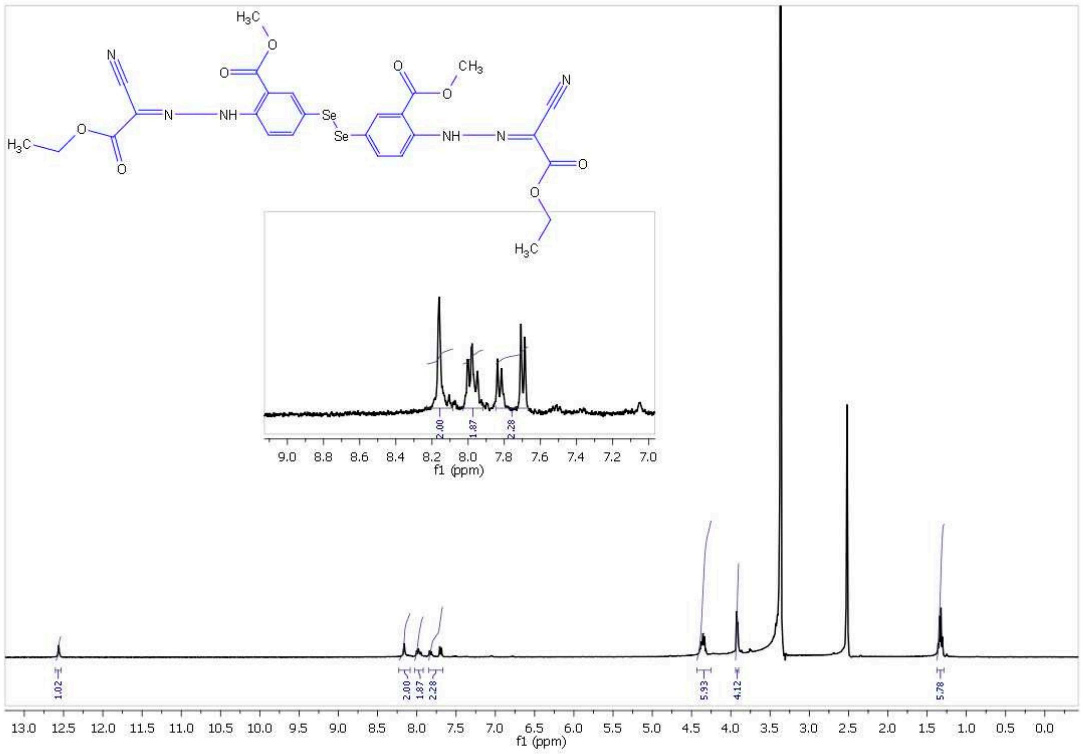

Figure S9. ^{1}HNMR chart of compound **6**

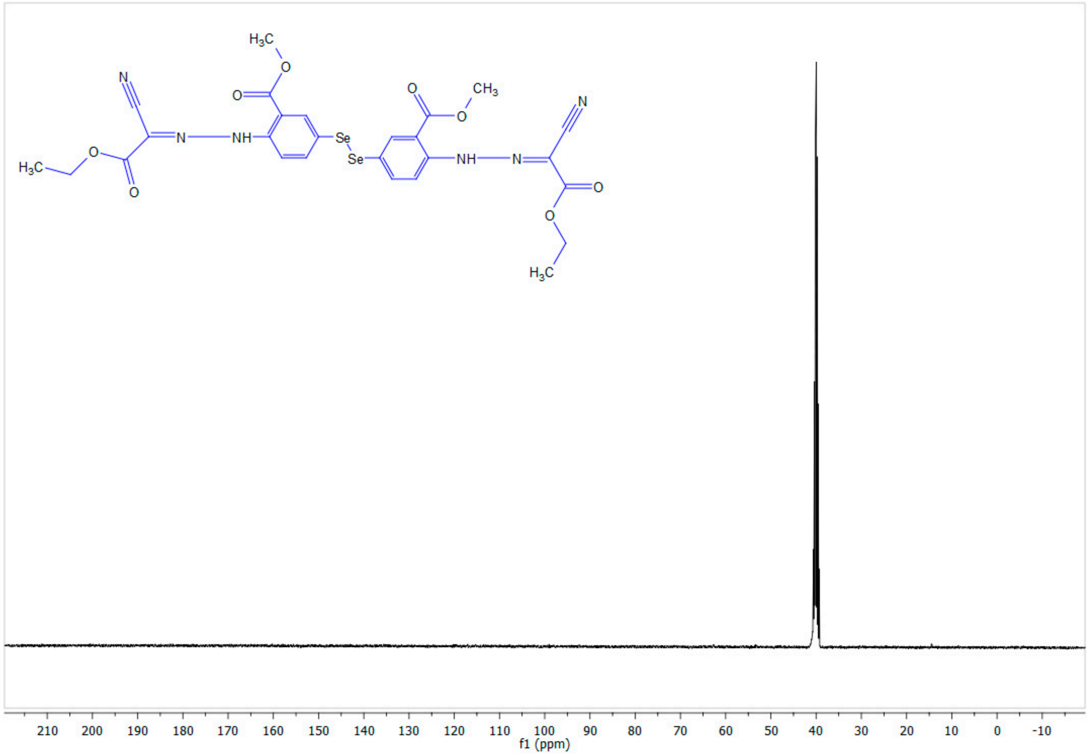

Figure S10. ^{13}CNMR chart of compound **6**

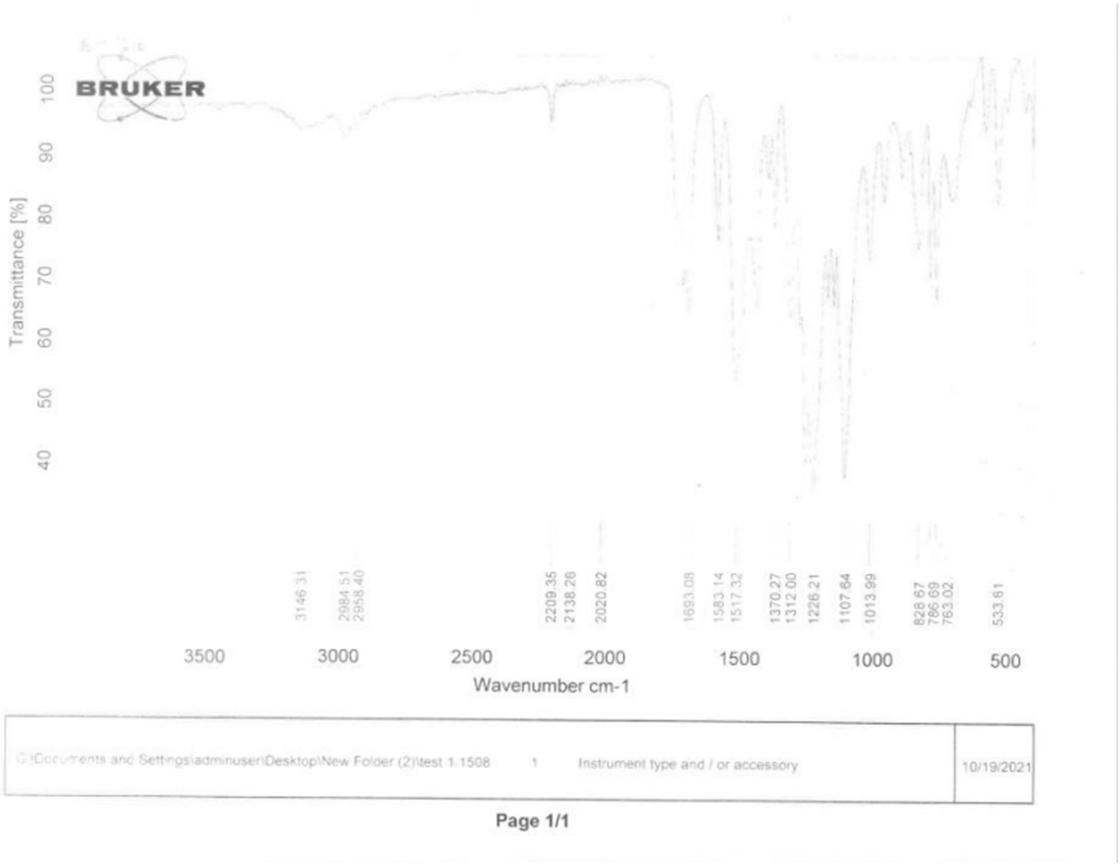

Figure S11. IR chart of compound **6**

Cairo University
Micro Analytical Center

DI Analysis
Shimadzu Qp-2010 Plus

Sample Information		Method	
Analyzed by	: Dr. Mai Younis	—— Analytical Line 1 ——	
Analyzed	: 10/01/2007 02:24:35 ص	IonSourceTemp	:250.00 °C
Sample Name	: B33	[MS Table]	
Sample ID	:	--Group 1 - Event 1--	
Customer Name	: Dr.Mohamed Soliman - Science - Cairo	Start Time	:0.00min
Data File	: C:\GCMSsolution\Data\Project1\B33.QGD	End Time	:10.00min
Org Data File	: C:\GCMSsolution\Data\Project1\B33.QGD	ACQ Mode	:Scan
Method File	: C:\GCMSsolution\Data\Project1\High Temperature Op	Event Time	:0.50sec
Org Method File	: C:\GCMSsolution\Data\Project1\High Temperature Op	Scan Speed	:2000
Report File	:	Start m/z	:50.00
Tuning File	: C:\GCMSsolution\System\Tune1_default.qgt	End m/z	:1000.00
$EndIf$Modified by	: Dr. Mai Younis		
Modified	: 10/01/2007 02:34:35 ص	Electron Voltage	: 70 eV
		Ionization Mode	: EI

C:\GCMSsolution\Data\Project1\B33.QGD

Chromatogram B33 C:\GCMSsolution\Data\Project1\B33.QGD

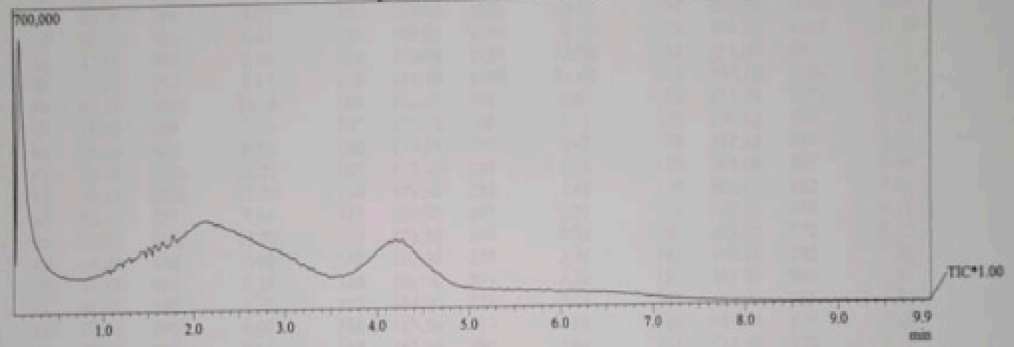

Spectrum

Line#:1 R.Time:4.2(Scan#:507)
MassPeaks:211
RawMode:Single 4.2(507) BasePeak:59(10447)
BG Mode:None Group 1 - Event 1

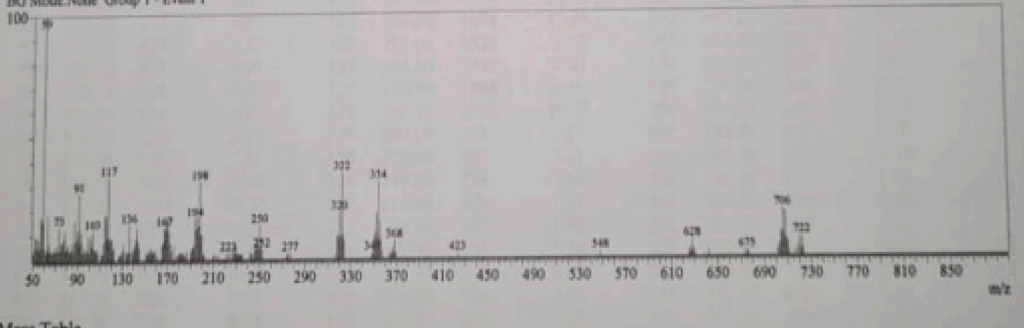

Mass Table
Line#:1 R.Time:4.2(Scan#:507)
MassPeaks:211
RawMode:Single 4.2(507) BasePeak:59(10447)
BG Mode:None Group 1 - Event 1

#	m/z	Abs. In	Rel. Int.	#	m/z	Abs. In	Rel. Int.	#	m/z	Abs. In	Rel. Int.
1	50.00	329	3.15	4	53.05	980	9.38	7	56.15	496	4.75
2	51.00	529	5.06	5	54.10	540	5.17	8	57.10	1842	17.63
3	52.05	996	9.53	6	55.10	874	8.37	9	58.15	1622	15.53

1 / 2

Figure S12. Mass chart of compound **6**

#	m/z	Abs. In	Rel. Int.
10	59.10	10447	100.00
11	60.10	566	5.42
12	61.10	330	3.16
13	62.15	462	4.42
14	63.05	1936	18.53
15	64.05	691	6.61
16	65.00	417	3.99
17	66.00	281	2.69
18	67.00	425	4.07
19	68.00	385	3.69
20	69.00	537	5.14
21	70.00	377	3.61
22	71.15	662	6.34
23	72.20	422	4.04
24	73.15	1417	13.56
25	74.20	433	4.14
26	75.15	775	7.42
27	76.15	685	6.56
28	77.15	906	8.67
29	78.10	1233	11.80
30	79.10	426	4.08
31	80.10	537	5.14
32	81.10	692	6.62
33	82.10	382	3.66
34	83.10	399	3.82
35	84.10	482	4.61
36	85.15	783	7.49
37	86.10	330	3.16
38	87.15	1699	16.26
39	88.10	769	7.36
40	89.10	980	9.38
41	90.10	1276	12.21
42	91.15	2828	27.07
43	92.15	777	7.44
44	93.05	1209	11.57
45	94.10	319	3.05
46	95.10	551	5.27
47	96.10	348	3.33
48	97.10	490	4.69
49	98.20	398	3.81
50	99.20	1076	10.30
51	100.10	311	2.98
52	101.15	959	9.18
53	102.10	604	5.78
54	103.20	1239	11.86
55	104.20	503	4.81
56	105.20	433	4.14
57	106.20	452	4.33
58	107.20	415	3.97
59	108.20	262	2.51
60	111.20	274	2.62
61	112.20	222	2.13
62	113.15	745	7.13
63	114.15	1923	18.41
64	115.20	1895	18.14
65	116.20	954	9.13
66	117.20	3516	33.66
67	118.10	900	8.61
68	119.15	949	9.08
69	120.20	860	8.23
70	121.20	434	4.15
71	122.05	597	5.71
72	127.10	218	2.09
73	128.10	217	2.08
74	129.10	209	2.00
75	130.10	414	3.96
76	131.20	686	6.57
77	133.20	353	3.38

#	m/z	Abs. In	Rel. Int.
78	134.20	279	2.67
79	135.20	359	3.44
80	136.20	1486	14.22
81	137.20	401	3.84
82	139.20	338	3.24
83	140.20	492	4.71
84	141.10	634	6.07
85	142.10	906	8.67
86	143.10	1346	12.88
87	144.10	777	7.44
88	145.10	502	4.81
89	151.10	319	3.05
90	152.10	244	2.34
91	153.10	385	3.69
92	154.10	290	2.78
93	155.10	521	4.99
94	156.10	439	4.20
95	157.10	330	3.16
96	158.10	337	3.23
97	159.10	450	4.31
98	161.10	228	2.18
99	165.10	594	5.69
100	166.05	758	7.26
101	167.10	1362	13.04
102	168.05	1263	12.09
103	169.05	1748	16.73
104	170.05	1649	15.78
105	171.10	1199	11.48
106	172.10	695	6.65
107	173.10	388	3.71
108	175.25	592	5.67
109	177.30	204	1.95
110	179.30	252	2.41
111	180.30	241	2.31
112	181.30	343	3.28
113	182.30	257	2.46
114	183.30	303	2.90
115	184.30	226	2.16
116	185.30	214	2.05
117	187.30	382	3.66
118	190.30	286	2.74
119	191.05	450	4.31
120	192.00	542	5.19
121	193.05	579	5.54
122	194.00	1745	16.70
123	195.05	1275	12.20
124	196.00	1874	17.94
125	197.00	1420	13.59
126	198.00	3302	31.61
127	198.95	1086	10.40
128	200.00	1052	10.07
129	201.00	263	2.52
130	202.00	222	2.13
131	210.00	239	2.29
132	211.00	228	2.18
133	214.00	207	1.98
134	221.00	238	2.28
135	223.00	278	2.66
136	225.00	314	3.01
137	227.00	249	2.38
138	228.00	490	4.69
139	229.00	241	2.31
140	230.00	551	5.27
141	231.00	249	2.38
142	232.00	241	2.31
143	233.00	201	1.92
144	234.00	218	2.09
145	235.00	210	2.01

#	m/z	Abs. In	Rel. Int.
146	242.00	268	2.57
147	243.00	273	2.61
148	244.00	226	2.16
149	246.00	378	3.62
150	247.05	660	6.32
151	248.00	953	9.12
152	249.00	450	4.31
153	250.10	1441	13.79
154	251.10	647	6.19
155	252.10	422	4.04
156	274.10	204	1.95
157	275.10	228	2.18
158	277.10	249	2.38
159	316.10	228	2.18
160	318.15	975	9.33
161	319.20	961	9.20
162	320.15	1969	18.85
163	321.15	934	8.94
164	322.15	3662	35.05
165	323.15	1044	9.99
166	324.15	786	7.52
167	348.20	209	2.00
168	349.20	246	2.35
169	350.20	871	8.34
170	351.20	1094	10.47
171	352.20	1921	18.39
172	353.20	847	8.11
173	354.15	3270	31.30
174	355.10	1572	15.05
175	356.10	774	7.41
176	357.10	340	3.25
177	364.10	255	2.44
178	365.10	212	2.03
179	366.10	450	4.31
180	367.10	225	2.15
181	368.15	754	7.22
182	369.20	263	2.52
183	423.20	206	1.97
184	548.20	249	2.38
185	625.20	220	2.11
186	626.20	353	3.38
187	627.20	215	2.06
188	628.20	698	6.68
189	629.20	330	3.16
190	630.20	218	2.09
191	642.20	330	3.16
192	673.20	206	1.97
193	675.20	222	2.13
194	676.20	207	1.98
195	701.20	246	2.35
196	702.20	369	3.53
197	703.20	535	5.12
198	704.30	1047	10.02
199	705.25	915	8.76
200	706.25	1965	18.81
201	707.20	922	8.83
202	708.25	1884	18.03
203	709.20	700	6.70
204	710.20	633	6.06
205	711.20	220	2.11
206	716.20	204	1.95
207	718.20	380	3.64
208	720.25	751	7.19
209	721.30	298	2.85
210	722.25	806	7.72
211	723.30	319	3.05

2 / 2

Mass chart of compound **6**

Dimethyl 5,5'-diselanediylbis(2-formamidobenzoate) (7).

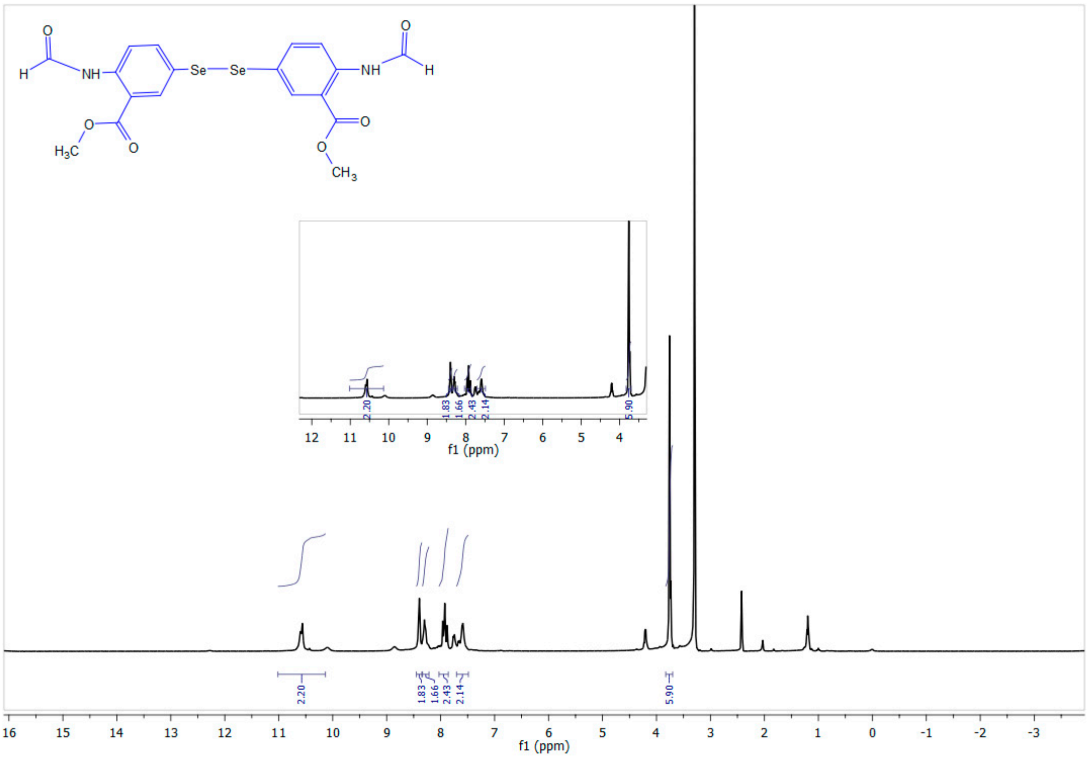

Figure S13. ^{1}HNMR chart of compound **7**

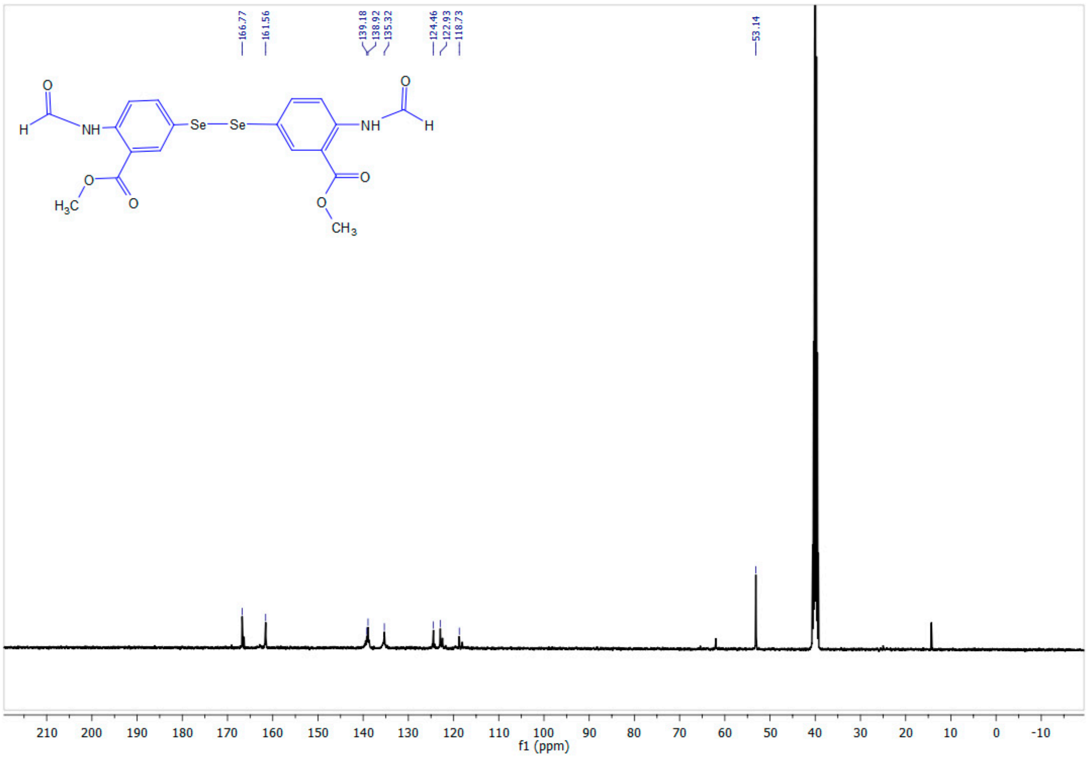

Figure S14. ^{13}CNMR chart of compound **7**

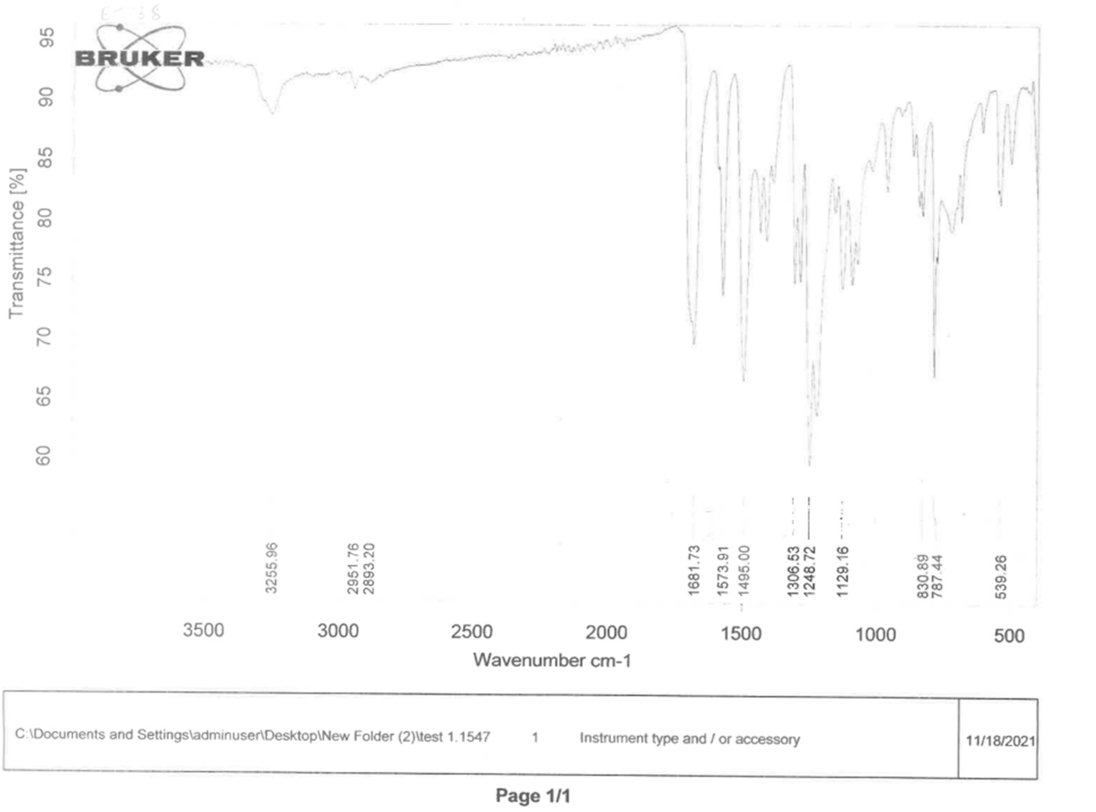

Figure S15. IR chart of compound **7**

Cairo University
Micro Analytical Center

DI Analysis
Shimadzu Qp-2010 Plus

Sample Information		Method	
Analyzed by	: Dr. Mai Younis	——— Analytical Line 1 ———	
Analyzed	: 10/01/2007 03:21:04 ص	IonSourceTemp	:250.00 °C
Sample Name	: B21	[MS Table]	
Sample ID	:	--Group 1 - Event 1--	
Customer Name	: Dr Mohamed Soliman - Science - Cairo	Start Time	:0.00min
Data File	: C:\GCMSsolution\Data\Project1\B21.QGD	End Time	:10.00min
Org Data File	: C:\GCMSsolution\Data\Project1\B21.QGD	ACQ Mode	:Scan
Method File	: C:\GCMSsolution\Data\Project1\High Temperature Op	Event Time	:0.50sec
Org Method File	: C:\GCMSsolution\Data\Project1\High Temperature Op	Scan Speed	:2000
Report File	:	Start m/z	:50.00
Tuning File	: C:\GCMSsolution\System\Tune1_default.qgt	End m/z	:1000.00
$Endif$Modified by	: Dr. Mai Younis		
Modified	: 10/01/2007 03:25:38 ص	Electron Voltage	: 70 eV
		Ionization Mode	: EI

C:\GCMSsolution\Data\Project1\B21.QGD

Chromatogram B21 C:\GCMSsolution\Data\Project1\B21.QGD

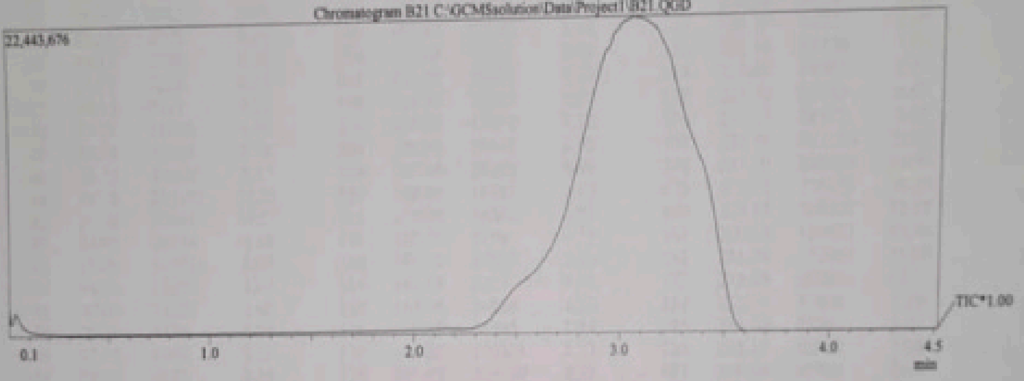

Spectrum

Line#:1 R.Time:3.1(Scan#:377)
MassPeaks:570
RawMode:Single 3.1(377) BasePeak:198(1464969)
BG Mode:None Group 1 - Event 1

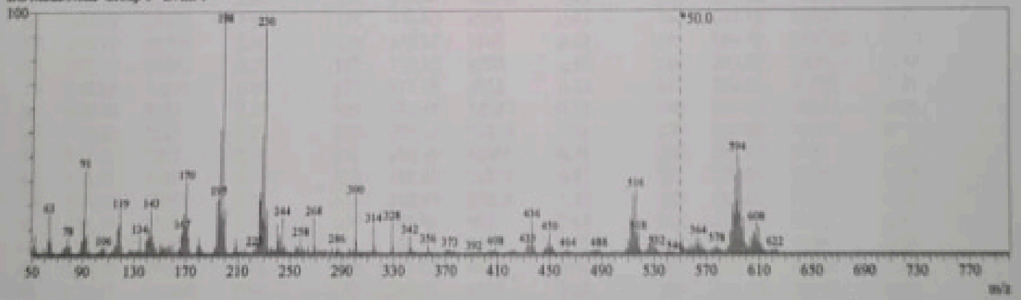

Mass Table
Line#:1 R.Time:3.1(Scan#:377)
MassPeaks:570
RawMode:Single 3.1(377) BasePeak:198(1464969)
BG Mode:None Group 1 - Event 1

#	m/z	Abs. In	Rel. Int.	#	m/z	Abs. In	Rel. Int.	#	m/z	Abs. In	Rel. Int.
1	50.05	24594	1.68	4	53.05	42252	2.88	7	56.05	3027	0.21
2	51.05	55368	3.78	5	54.05	20407	1.39	8	57.05	6534	0.45
3	52.05	116377	7.94	6	55.05	9644	0.66	9	58.05	9131	0.62

1 / 4

Figure S16. Mass chart of compound **7**

#	m/z	Abs. In	Rel. Int.	#	m/z	Abs. In	Rel. Int.	#	m/z	Abs. In	Rel. Int.
10	59.00	36338	2.48	79	128.10	19204	1.31	148	197.05	239788	16.37
11	60.15	5776	0.39	80	129.15	18863	1.29	149	198.00	146496	100.00
12	61.15	23145	1.58	81	130.10	24905	1.70	150	198.95	206870	14.12
13	62.15	80056	5.46	82	131.15	15227	1.04	151	200.00	265948	18.15
14	63.05	234780	16.03	83	132.15	15925	1.09	152	200.95	33569	2.29
15	64.05	103512	7.07	84	133.25	21682	1.48	153	201.95	4387	0.30
16	65.05	62224	4.25	85	134.20	108370	7.40	154	203.10	2042	0.14
17	66.05	17090	1.17	86	135.15	35114	2.40	155	204.10	2559	0.17
18	67.05	10927	0.75	87	136.05	8126	0.55	156	205.05	3675	0.25
19	68.05	5612	0.38	88	137.05	12597	0.86	157	206.15	7640	0.52
20	69.05	6453	0.44	89	138.05	18067	1.23	158	207.15	34027	2.32
21	70.10	2214	0.15	90	139.05	70881	4.84	159	208.15	96895	6.61
22	71.10	4134	0.28	91	140.05	87187	5.95	160	209.10	55556	3.79
23	72.05	2060	0.14	92	141.05	150661	10.28	161	210.10	18156	1.24
24	73.05	9461	0.65	93	142.05	98499	6.72	162	211.05	7541	0.51
25	74.05	27743	1.89	94	143.05	262806	17.94	163	212.05	12728	0.87
26	75.05	39024	2.66	95	144.05	107906	7.37	164	213.05	10172	0.69
27	76.05	47488	3.24	96	145.05	69056	4.71	165	214.05	17585	1.20
28	77.05	52825	3.61	97	146.10	44022	3.00	166	215.05	5925	0.40
29	78.05	96196	6.57	98	147.10	34598	2.36	167	216.05	32339	2.21
30	79.05	41615	2.84	99	148.10	8207	0.56	168	216.95	6128	0.42
31	79.95	52962	3.62	100	149.25	11918	0.81	169	218.05	8970	0.61
32	81.05	7497	0.51	101	150.15	58987	4.03	170	219.05	4734	0.32
33	82.00	8503	0.58	102	151.15	51884	3.54	171	220.05	4698	0.32
34	83.15	4730	0.32	103	151.95	28210	1.93	172	221.10	11732	0.80
35	84.15	3783	0.26	104	152.95	38175	2.61	173	222.10	15578	1.06
36	85.15	5226	0.36	105	153.95	45823	3.13	174	223.05	34599	2.36
37	86.15	3311	0.23	106	154.95	28497	1.95	175	224.10	53783	3.67
38	87.15	11655	0.80	107	155.95	47292	3.23	176	225.15	88572	6.05
39	88.15	34322	2.34	108	156.95	39418	2.69	177	226.10	317178	21.65
40	89.15	80966	5.53	109	157.90	58238	3.98	178	227.10	365674	24.96
41	90.15	208182	14.21	110	158.95	16555	1.13	179	228.05	716179	48.89
42	91.05	506455	34.57	111	159.90	54662	3.73	180	229.15	204352	13.95
43	92.05	91654	6.26	112	160.95	5109	0.35	181	230.05	136627	93.26
44	93.00	117914	8.05	113	161.90	16975	1.16	182	231.05	171303	11.69
45	94.05	15285	1.04	114	163.05	5277	0.36	183	232.05	267631	18.27
46	95.00	24652	1.68	115	164.05	24885	1.70	184	232.95	37429	2.55
47	96.05	4228	0.29	116	165.05	34245	2.34	185	234.10	18561	1.27
48	97.15	4299	0.29	117	166.05	110381	7.53	186	235.15	28389	1.94
49	98.15	3622	0.25	118	167.05	136789	9.34	187	236.15	45786	3.13
50	99.10	3818	0.26	119	168.05	240735	16.43	188	237.15	18519	1.26
51	100.15	3684	0.25	120	169.05	157134	10.73	189	238.15	14271	0.97
52	101.15	8551	0.58	121	170.05	431966	29.49	190	239.25	26021	1.78
53	102.10	11721	0.80	122	171.05	149906	10.23	191	240.15	176287	12.03
54	103.15	20032	1.37	123	172.00	101633	6.94	192	241.15	82697	5.64
55	104.15	38808	2.65	124	173.00	28470	1.94	193	242.10	113735	7.76
56	105.05	27074	1.85	125	174.00	6508	0.44	194	243.15	32347	2.21
57	106.10	38996	2.66	126	175.15	1968	0.13	195	244.10	213769	14.59
58	107.10	33393	2.28	127	176.15	1458	0.10	196	245.05	34991	2.39
59	108.05	10011	0.68	128	177.15	3821	0.26	197	246.05	47288	3.23
60	109.10	4231	0.29	129	178.15	12523	0.85	198	247.05	13842	0.94
61	110.10	3010	0.21	130	179.15	51078	3.49	199	248.00	11108	0.76
62	111.10	5324	0.36	131	180.15	92866	6.34	200	249.05	9182	0.63
63	112.15	5457	0.37	132	181.15	50767	3.47	201	250.05	12407	0.85
64	113.10	19951	1.36	133	182.10	28319	1.93	202	251.00	9151	0.62
65	114.10	27367	1.87	134	183.05	9951	0.68	203	252.15	6532	0.45
66	115.05	56985	3.89	135	184.05	7677	0.52	204	253.25	7790	0.53
67	116.15	48386	3.30	136	185.05	4424	0.30	205	254.15	46608	3.18
68	117.05	145589	9.94	137	186.05	4173	0.28	206	255.10	29241	2.00
69	118.15	175690	11.99	138	187.05	2929	0.20	207	256.05	47888	3.27
70	119.10	263689	18.00	139	188.05	2012	0.14	208	257.15	25607	1.75
71	120.10	55866	3.81	140	189.05	2466	0.17	209	258.10	89121	6.08
72	121.10	10935	0.75	141	190.05	3154	0.22	210	259.05	36516	2.49
73	122.15	5648	0.39	142	191.05	6704	0.46	211	260.10	35807	2.44
74	123.15	4246	0.29	143	192.05	33178	2.26	212	261.10	17399	1.19
75	124.15	3163	0.22	144	193.05	42554	2.90	213	262.15	10618	0.72
76	125.15	9613	0.66	145	194.05	314135	21.44	214	263.20	21540	1.47
77	126.15	19499	1.33	146	195.05	336790	22.99	215	264.15	48109	3.28
78	127.15	34671	2.37	147	196.00	746338	50.95	216	265.15	11512	0.79

2 / 4

Mass chart of compound **7**

#	m/z	Abs. In	Rel. Int.	#	m/z	Abs. In	Rel. Int.	#	m/z	Abs. In	Rel. Int.
217	266.20	5574	0.38	286	335.05	4966	0.34	355	405.20	11647	0.80
218	267.25	18289	1.25	287	336.00	10442	0.71	356	406.20	19190	1.31
219	268.20	214457	14.64	288	337.05	3758	0.26	357	407.25	7865	0.54
220	269.15	50722	3.46	289	338.00	11316	0.77	358	408.20	34479	2.35
221	270.05	18993	1.30	290	339.20	3492	0.24	359	409.15	9079	0.62
222	271.15	8270	0.56	291	340.15	5991	0.41	360	410.15	7270	0.50
223	272.10	22950	1.57	292	341.25	11436	0.78	361	411.10	1921	0.13
224	273.05	12189	0.83	293	342.25	102667	7.01	362	412.15	716	0.05
225	274.00	18033	1.23	294	343.15	33975	2.32	363	413.15	1117	0.08
226	275.00	12638	0.86	295	344.05	11641	0.79	364	414.15	977	0.07
227	276.00	23305	1.59	296	345.10	14649	1.00	365	415.15	2414	0.16
228	277.05	10220	0.70	297	346.10	6401	0.44	366	416.25	1818	0.12
229	277.95	26831	1.83	298	347.05	4322	0.30	367	417.20	4686	0.32
230	278.95	7502	0.51	299	348.10	6097	0.42	368	418.20	6340	0.43
231	280.00	12959	0.88	300	349.05	2676	0.18	369	419.20	8022	0.55
232	281.25	7089	0.48	301	350.05	3422	0.23	370	420.20	12442	0.85
233	282.20	36882	2.52	302	351.15	2694	0.18	371	421.20	9254	0.63
234	283.15	11288	0.77	303	352.05	3204	0.22	372	422.20	23462	1.60
235	284.15	6929	0.47	304	353.10	3617	0.25	373	423.05	12365	0.84
236	285.25	10309	0.70	305	354.25	2467	0.17	374	424.05	7087	0.48
237	286.20	47882	3.27	306	355.25	7315	0.50	375	425.05	8548	0.58
238	287.05	19396	1.32	307	356.25	46877	3.20	376	426.00	2537	0.17
239	288.05	16595	1.13	308	357.15	13638	0.93	377	427.05	3033	0.21
240	289.05	13257	0.90	309	358.15	3305	0.23	378	428.05	1265	0.09
241	290.05	6268	0.43	310	359.10	1993	0.14	379	429.15	1679	0.11
242	291.10	4498	0.31	311	360.15	1330	0.09	380	430.15	4607	0.31
243	292.15	2391	0.16	312	361.15	1094	0.07	381	431.15	5087	0.35
244	293.15	3914	0.27	313	362.15	1676	0.11	382	432.15	37770	2.58
245	294.25	4362	0.30	314	363.05	962	0.07	383	433.15	47768	3.26
246	295.20	20102	1.37	315	364.15	1081	0.07	384	434.15	101178	6.91
247	296.15	27404	1.87	316	365.15	869	0.06	385	435.25	43189	2.95
248	297.15	7767	0.53	317	366.15	796	0.05	386	436.20	196380	13.41
249	298.25	4513	0.31	318	367.25	2136	0.15	387	437.10	54367	3.71
250	299.25	26163	1.79	319	368.25	2672	0.18	388	438.15	42121	2.88
251	300.20	341862	23.34	320	369.20	8393	0.57	389	439.10	11220	0.77
252	301.15	78463	5.36	321	370.25	23395	1.60	390	440.05	2091	0.14
253	302.15	15487	1.06	322	371.15	19186	1.31	391	441.10	658	0.04
254	303.15	5962	0.41	323	372.15	9747	0.67	392	442.15	451	0.03
255	304.00	9417	0.64	324	373.10	26022	1.78	393	443.25	974	0.07
256	305.00	6111	0.42	325	374.10	12108	0.83	394	444.20	3310	0.23
257	306.00	11102	0.76	326	375.15	10231	0.70	395	445.25	4151	0.28
258	307.00	6762	0.46	327	376.15	15771	1.08	396	446.20	25433	1.74
259	308.00	10218	0.70	328	377.10	5465	0.37	397	447.20	31066	2.12
260	309.05	8278	0.57	329	378.15	4515	0.31	398	448.20	64380	4.39
261	310.05	14253	0.97	330	379.15	1506	0.10	399	449.25	34343	2.34
262	311.20	4722	0.32	331	380.20	1839	0.13	400	450.20	122422	8.36
263	312.15	8322	0.57	332	381.20	630	0.04	401	451.10	48983	3.34
264	313.25	18558	1.27	333	382.35	803	0.05	402	452.15	32570	2.22
265	314.20	172178	11.75	334	383.35	1024	0.07	403	453.15	21234	1.45
266	315.15	47455	3.24	335	384.30	3825	0.26	404	454.00	6215	0.42
267	316.00	20338	1.39	336	385.25	2413	0.16	405	455.05	5959	0.41
268	317.00	9206	0.63	337	386.75	2422	0.17	406	456.05	3265	0.22
269	318.00	8063	0.55	338	387.65	2694	0.18	407	457.10	2191	0.15
270	319.05	4177	0.29	339	388.75	2477	0.17	408	458.10	2047	0.14
271	320.00	6754	0.46	340	389.75	4577	0.31	409	459.15	1616	0.11
272	321.10	3839	0.26	341	390.65	4336	0.30	410	460.20	6214	0.42
273	322.05	3004	0.21	342	391.75	4915	0.34	411	461.25	6400	0.44
274	323.20	2350	0.16	343	392.75	3792	0.26	412	462.20	14204	0.97
275	324.15	2187	0.15	344	393.75	6278	0.43	413	463.25	5914	0.40
276	325.15	1645	0.11	345	394.70	4116	0.28	414	464.20	24223	1.65
277	326.25	2909	0.20	346	395.75	6297	0.43	415	465.15	7959	0.54
278	327.25	12951	0.88	347	396.75	3150	0.22	416	466.20	6337	0.43
279	328.25	185457	12.66	348	397.75	6331	0.43	417	467.15	2907	0.20
280	329.15	43614	2.98	349	399.25	4067	0.28	418	468.05	1516	0.10
281	330.15	8245	0.56	350	400.15	3944	0.27	419	469.05	1649	0.11
282	331.05	2873	0.20	351	401.20	6143	0.42	420	470.05	1528	0.10
283	332.05	4167	0.28	352	402.15	4691	0.32	421	471.15	1527	0.10
284	333.05	3022	0.21	353	403.20	10460	0.71	422	472.05	2043	0.14
285	334.00	7474	0.51	354	404.15	11730	0.80	423	473.05	1822	0.12

3 / 4

Mass chart of compound **7**

#	m/z	Abs. In	Rel. Int.	#	m/z	Abs. In	Rel. Int.	#	m/z	Abs. In	Rel. Int.
424	474.05	2280	0.16	473	523.15	8091	0.55	522	574.05	505	0.03
425	475.15	2371	0.16	474	524.15	21834	1.49	523	575.00	247	0.02
426	476.05	2926	0.20	475	525.15	22476	1.53	524	576.05	680	0.05
427	477.15	3029	0.21	476	526.15	55056	3.76	525	576.95	551	0.04
428	478.05	4215	0.29	477	527.15	43844	2.99	526	578.00	972	0.07
429	479.15	3675	0.25	478	528.10	92798	6.33	527	579.00	415	0.03
430	480.05	5111	0.35	479	529.15	35399	2.42	528	579.85	565	0.04
431	481.15	6041	0.41	480	530.10	99618	6.80	529	580.90	292	0.02
432	482.10	9151	0.62	481	531.10	27508	1.88	530	581.90	295	0.02
433	483.10	9470	0.65	482	532.10	32623	2.23	531	583.90	313	0.02
434	484.10	16985	1.16	483	533.10	9073	0.62	532	585.00	257	0.02
435	485.10	11826	0.81	484	534.10	4012	0.27	533	586.05	744	0.05
436	486.10	22422	1.53	485	535.15	1423	0.10	534	587.05	809	0.06
437	487.15	8484	0.58	486	536.15	1038	0.07	535	588.00	2423	0.17
438	488.10	23593	1.61	487	537.20	1092	0.07	536	589.05	2699	0.18
439	489.10	5918	0.40	488	538.20	2463	0.17	537	590.05	5818	0.40
440	490.10	7351	0.50	489	539.25	2519	0.17	538	591.05	4705	0.32
441	491.15	2224	0.15	490	540.15	5813	0.40	539	592.05	9235	0.63
442	492.05	1433	0.10	491	541.20	4666	0.32	540	593.05	6356	0.43
443	493.15	895	0.06	492	542.15	8850	0.60	541	594.00	11919	0.81
444	494.15	1397	0.10	493	543.15	3404	0.23	542	595.05	4809	0.33
445	495.15	1521	0.10	494	544.15	10121	0.69	543	596.05	9954	0.68
446	496.15	2606	0.18	495	545.15	2675	0.18	544	597.05	2893	0.20
447	497.15	2497	0.17	496	546.15	3410	0.23	545	598.05	4235	0.29
448	498.20	4898	0.33	497	547.20	1230	0.08	546	599.10	1218	0.08
449	499.15	3785	0.26	498	548.15	591	0.04	547	600.10	1034	0.07
450	500.15	6737	0.46	499	549.50	644	0.04	548	601.05	567	0.04
451	501.15	3026	0.21	500	550.75	485	0.03	549	602.05	897	0.06
452	502.10	6652	0.45	501	551.80	895	0.06	550	603.05	765	0.05
453	503.15	2490	0.17	502	552.75	535	0.04	551	604.05	1812	0.12
454	504.10	2625	0.18	503	554.10	634	0.04	552	605.15	1443	0.10
455	505.15	1233	0.08	504	555.20	423	0.03	553	606.10	2610	0.18
456	506.15	2859	0.20	505	556.20	698	0.05	554	607.05	2092	0.14
457	507.15	4058	0.28	506	557.10	402	0.03	555	608.05	3528	0.24
458	508.15	18323	1.25	507	558.10	958	0.07	556	609.05	1284	0.09
459	509.15	28542	1.95	508	559.15	515	0.04	557	610.05	2987	0.20
460	510.10	80658	5.51	509	560.10	1039	0.07	558	611.05	928	0.06
461	511.15	79167	5.40	510	561.05	753	0.05	559	612.00	1057	0.07
462	512.10	210383	14.36	511	562.10	1583	0.11	560	613.00	426	0.03
463	513.15	165589	11.30	512	563.10	938	0.06	561	614.00	295	0.02
464	514.10	346700	23.67	513	564.05	1915	0.13	562	615.00	231	0.02
465	515.15	130687	8.92	514	564.95	663	0.05	563	616.00	218	0.01
466	516.10	377607	25.78	515	566.00	1260	0.09	564	618.00	262	0.02
467	517.10	103930	7.09	516	566.95	441	0.03	565	619.00	242	0.02
468	518.10	122695	8.38	517	568.00	676	0.05	566	620.00	412	0.03
469	519.05	31496	2.15	518	569.00	327	0.02	567	621.00	337	0.02
470	520.10	15218	1.04	519	570.00	327	0.02	568	622.00	554	0.04
471	521.10	4263	0.29	520	572.00	258	0.02	569	623.00	289	0.02
472	522.15	5536	0.38	521	573.00	207	0.01	570	624.00	510	0.03

4 / 4

Mass chart of compound **7**

Dimethyl 5,5'-diselanediylbis(2-acetamidobenzoate) (***8***).

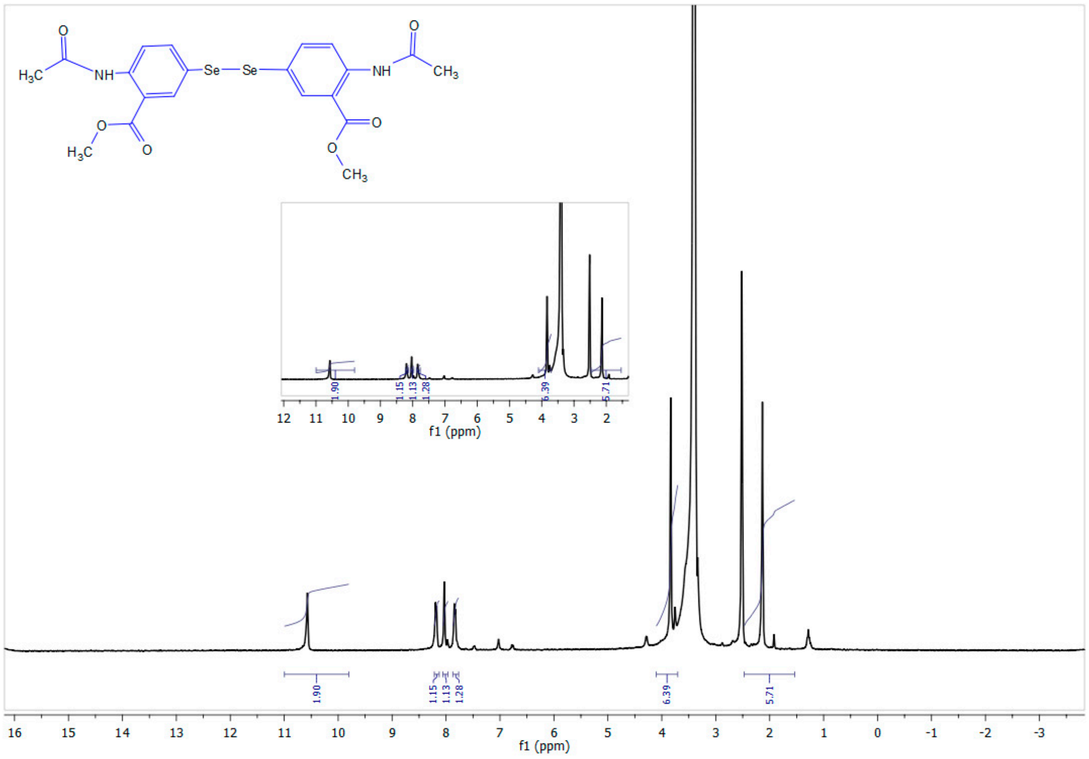

Figure S17. ^{1}HNMR chart of compound **8**

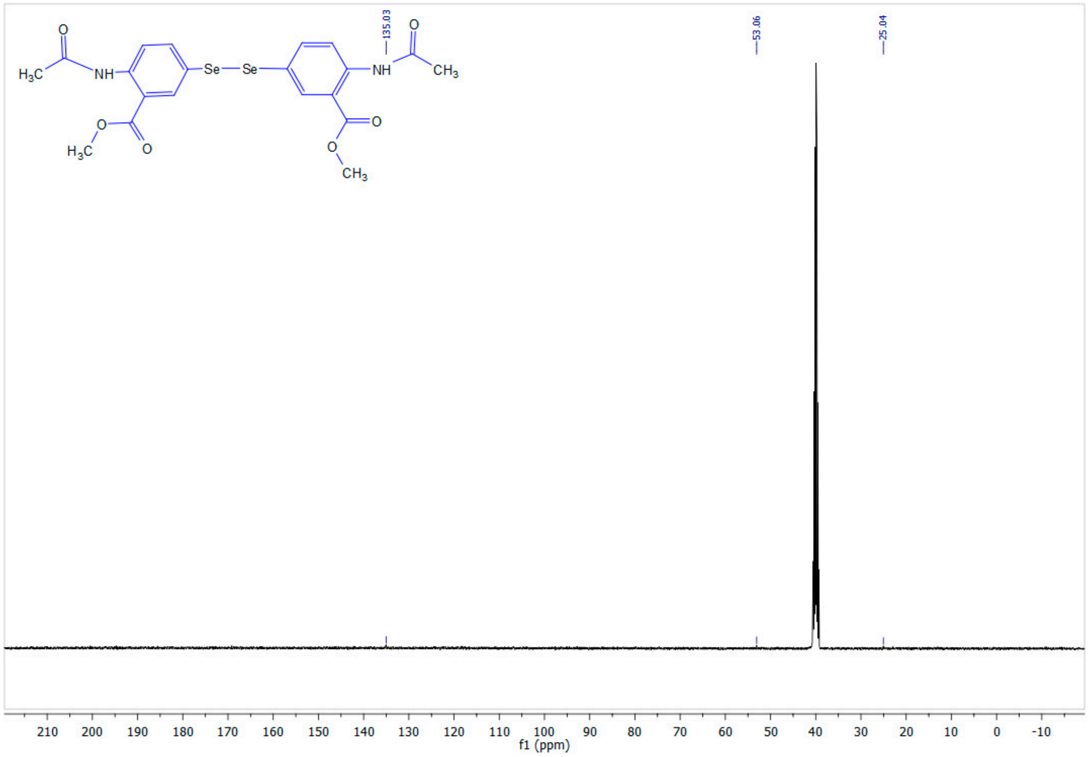

Figure S18. ^{13}CNMR chart of compound **8**

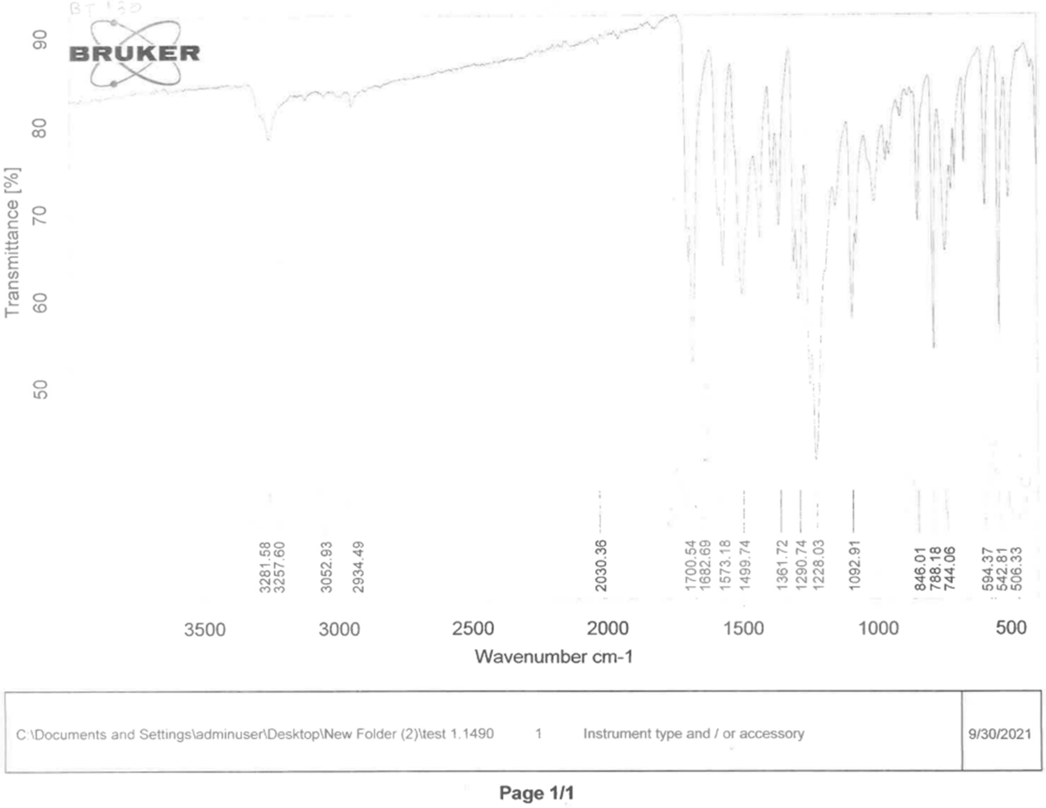

Figure S19. IR chart of compound **8**

26-Jan-07 03:22:33

Cairo University
Micro Analytical Center

DI Analysis
Shimadzu Qp-2010 Plus

Sample Information

Analyzed by	: Dr. Mai Younis
Analyzed	: 26/01/2007 03:04:59 ص
Sample Name	: B20
Sample ID	:
Customer Name	: Dr.Mohamed Soliman - Science - Cairo
Data File	: C:\GCMSsolution\Data\Project1\B20.QGD
Org Data File	: C:\GCMSsolution\Data\Project1\B20.QGD
Method File	: C:\GCMSsolution\Data\Project1\High Temperature Op
Org Method File	: C:\GCMSsolution\Data\Project1\High Temperature Op
Report File	:
Tuning File	: C:\GCMSsolution\System\Tune1_default.qgt
$EndIf$Modified by	: Dr. Mai Younis
Modified	: 26/01/2007 03:14:59 ص

Method

===== Analytical Line 1 =====	
IonSourceTemp	:250.00 °C
[MS Table]	
--Group 1 - Event 1--	
Start Time	:0.00min
End Time	:10.00min
ACQ Mode	:Scan
Event Time	:0.50sec
Scan Speed	:2000
Start m/z	:50.00
End m/z	:1000.00
Electron Voltage	: 70 eV
Ionization Mode	: EI

C:\GCMSsolution\Data\Project1\B20.QGD

Chromatogram B20 C:\GCMSsolution\Data\Project1\B20.QGD

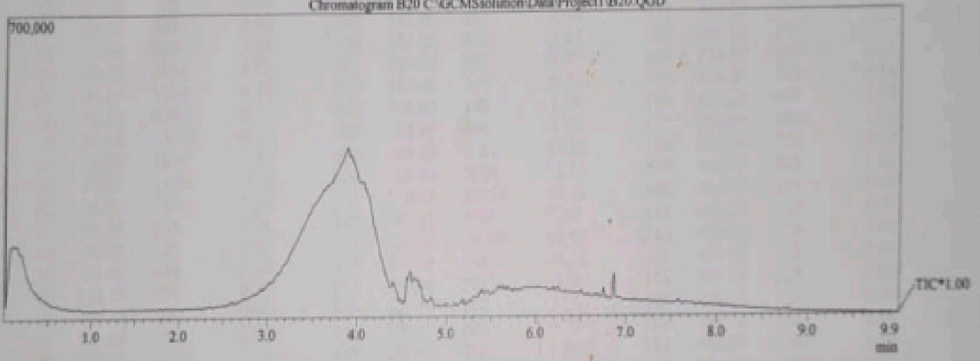

Spectrum

Line#:1 R.Time:3.9(Scan#:466)
MassPeaks:225(Peak Elimination m/z: 570.20)
RawMode:Single 3.9(466) BasePeak:230(33772)
BG Mode:None Group 1 - Event 1

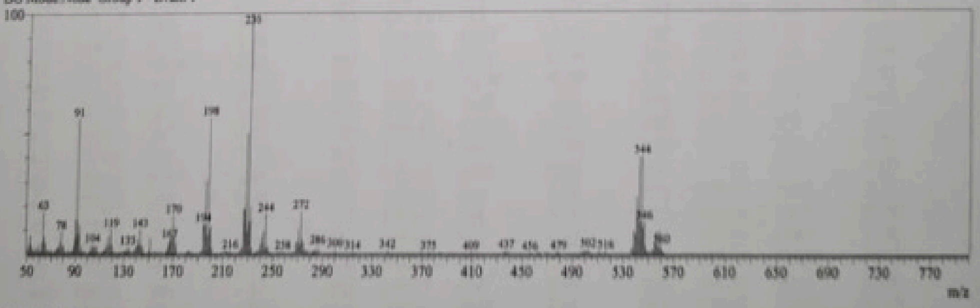

Mass Table
Line#:1 R.Time:3.9(Scan#:466)
MassPeaks:225(Peak Elimination m/z: 570.20)
RawMode:Single 3.9(466) BasePeak:230(33772)
BG Mode:None Group 1 - Event 1

#	m/z	Abs. In	Rel. Int.	#	m/z	Abs. In	Rel. Int.	#	m/z	Abs. In	Rel. Int.
1	50.00	503	1.49	4	53.05	1107	3.28	7	56.10	265	0.78
2	51.10	1241	3.67	5	54.10	612	1.81	8	57.10	674	2.00
3	52.10	2558	7.57	6	55.10	522	1.55	9	58.10	350	1.04

Figure S20. Mass chart of compound **8**

#	m/z	Abs. In	Rel. Int.	#	m/z	Abs. In	Rel. Int.	#	m/z	Abs. In	Rel. Int.
10	59.10	1150	3.41	79	141.10	2124	6.29	148	258.10	273	0.81
11	60.10	262	0.78	80	142.10	1193	3.53	149	266.10	234	0.69
12	61.10	506	1.50	81	143.10	3423	10.14	150	268.20	1455	4.31
13	62.15	1522	4.51	82	144.15	1202	3.56	151	269.20	1751	5.18
14	63.10	5911	17.50	83	145.05	866	2.56	152	270.20	3359	9.95
15	64.10	2404	7.12	84	146.10	302	0.89	153	271.25	1293	3.83
16	65.10	1428	4.23	85	150.25	468	1.39	154	272.15	5965	17.66
17	66.10	606	1.79	86	151.20	2284	6.76	155	273.10	1935	5.73
18	67.10	402	1.19	87	153.20	401	1.19	156	274.15	1566	4.64
19	68.10	263	0.78	88	155.20	258	0.76	157	275.10	511	1.51
20	69.10	545	1.61	89	156.20	297	0.88	158	276.10	406	1.20
21	70.10	249	0.74	90	158.20	242	0.72	159	278.10	484	1.43
22	71.10	266	0.79	91	159.20	210	0.62	160	280.10	234	0.69
23	73.10	303	0.90	92	161.20	202	0.60	161	282.10	306	0.91
24	74.10	714	2.11	93	164.20	212	0.63	162	283.10	310	0.92
25	75.10	783	2.32	94	165.15	640	1.90	163	284.10	610	1.81
26	76.15	822	2.43	95	166.10	1361	4.03	164	285.10	385	1.14
27	77.15	1711	5.07	96	167.10	1886	5.58	165	286.15	1014	3.00
28	78.10	3119	9.24	97	168.10	3095	9.16	166	287.20	265	0.78
29	79.20	1262	3.74	98	169.10	2214	6.56	167	300.20	559	1.66
30	80.15	1005	2.98	99	170.05	5367	15.89	168	314.20	247	0.73
31	81.20	351	1.04	100	171.10	2543	7.53	169	342.20	441	1.31
32	83.20	350	1.04	101	172.00	1362	4.03	170	369.20	207	0.61
33	84.20	223	0.66	102	173.00	492	1.46	171	375.20	222	0.66
34	85.20	335	0.99	103	181.00	257	0.76	172	384.20	226	0.67
35	87.20	420	1.24	104	182.00	420	1.24	173	409.20	246	0.73
36	88.15	1036	3.07	105	183.00	252	0.75	174	435.20	254	0.75
37	89.10	2164	6.41	106	185.00	247	0.73	175	436.20	255	0.76
38	90.15	4905	14.52	107	192.00	398	1.18	176	437.20	449	1.33
39	91.15	18935	56.07	108	193.10	466	1.38	177	439.20	449	1.33
40	92.05	2859	8.47	109	194.10	4161	12.32	178	456.20	226	0.67
41	93.05	4108	12.16	110	195.10	3886	11.51	179	462.20	302	0.89
42	94.00	383	1.13	111	196.05	10254	30.36	180	464.20	231	0.68
43	95.10	938	2.78	112	197.15	2506	7.42	181	468.20	260	0.77
44	96.10	225	0.67	113	198.05	19109	56.58	182	470.20	274	0.81
45	97.10	406	1.20	114	199.05	3453	10.22	183	477.20	215	0.64
46	101.10	238	0.70	115	200.05	3845	11.39	184	478.20	222	0.66
47	102.10	306	0.91	116	201.10	748	2.21	185	479.20	316	0.94
48	103.15	954	2.82	117	209.10	260	0.77	186	496.20	292	0.86
49	104.20	1278	3.78	118	211.10	350	1.04	187	498.20	420	1.24
50	105.15	849	2.51	119	212.10	367	1.09	188	499.20	318	0.94
51	106.10	1078	3.19	120	213.10	263	0.78	189	500.30	630	1.87
52	107.20	913	2.70	121	214.10	266	0.79	190	501.30	233	0.69
53	108.20	226	0.67	122	216.10	414	1.23	191	502.30	638	1.89
54	109.20	236	0.70	123	223.10	278	0.82	192	504.30	351	1.04
55	112.20	214	0.63	124	224.15	929	2.75	193	510.30	252	0.75
56	113.20	431	1.28	125	225.15	732	2.17	194	511.30	228	0.68
57	114.05	520	1.54	126	226.10	6334	18.76	195	512.30	263	0.78
58	115.05	1330	3.94	127	227.10	6332	18.75	196	515.30	215	0.64
59	116.10	806	2.39	128	228.10	16798	49.74	197	516.30	447	1.32
60	117.10	2417	7.16	129	229.15	3157	9.35	198	528.30	212	0.63
61	118.15	1273	3.77	130	230.10	33772	100.00	199	536.25	679	2.01
62	119.15	3449	10.21	131	231.05	4489	13.29	200	537.20	929	2.75
63	120.20	974	2.88	132	232.10	6570	19.45	201	538.25	3310	9.80
64	121.20	334	0.99	133	233.05	864	2.56	202	539.35	2783	8.24
65	123.20	225	0.67	134	236.10	206	0.61	203	540.30	7903	23.40
66	126.20	242	0.72	135	237.10	294	0.87	204	541.25	6033	17.86
67	127.20	244	0.72	136	238.10	487	1.44	205	542.30	13409	39.70
68	129.20	390	1.15	137	239.15	676	2.00	206	543.35	4404	13.04
69	130.20	354	1.05	138	240.15	1315	3.89	207	544.30	13558	40.15
70	131.20	332	0.98	139	241.15	2341	6.93	208	545.30	3666	10.86
71	132.20	703	2.08	140	242.15	3157	9.35	209	546.30	4534	13.43
72	133.20	946	2.80	141	243.25	991	2.93	210	547.25	1122	3.32
73	134.20	370	1.10	142	244.15	5586	16.54	211	548.15	686	2.03
74	135.20	602	1.78	143	245.15	702	2.08	212	549.10	242	0.72
75	137.20	324	0.96	144	246.15	1116	3.30	213	550.10	311	0.92
76	138.20	313	0.93	145	248.10	225	0.67	214	551.10	215	0.64
77	139.15	868	2.57	146	254.10	257	0.76	215	552.30	695	2.06
78	140.10	1078	3.19	147	256.10	231	0.68	216	553.30	588	1.74

2 / 3

Mass chart of compound **8**

dimethyl 5,5'-diselanediylbis(2-(2-chloroacetamido) benzoate) (**9**).

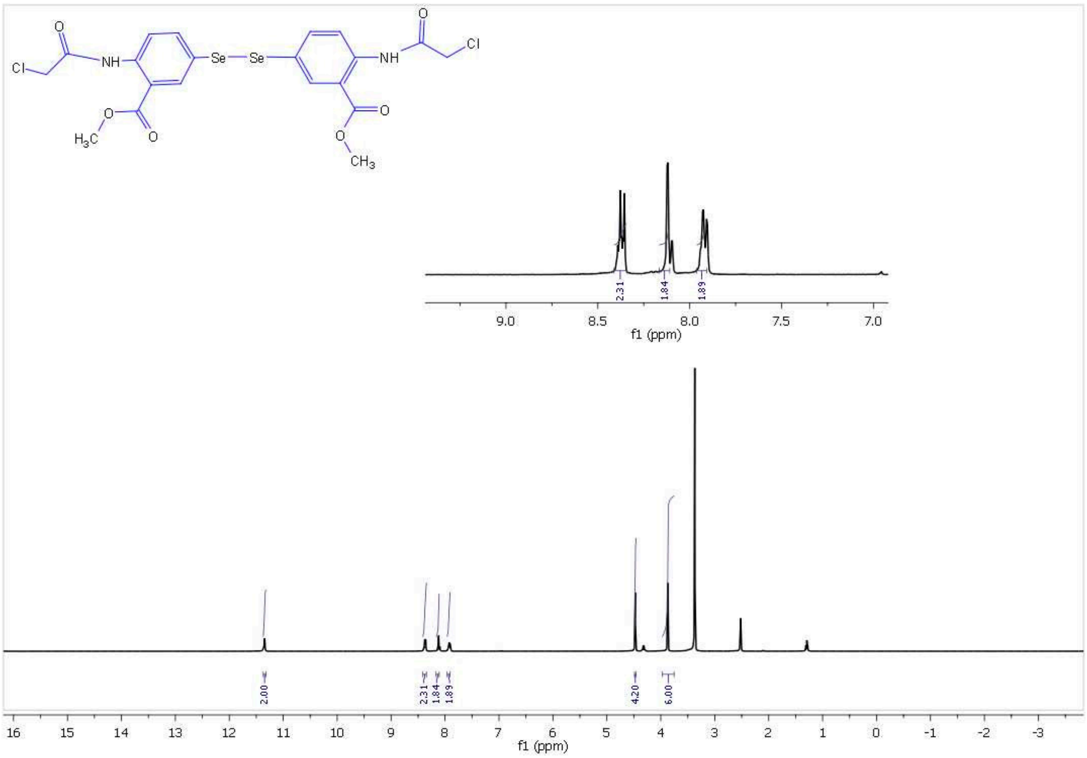

Figure S21. ^{1}HNMR chart of compound **9**

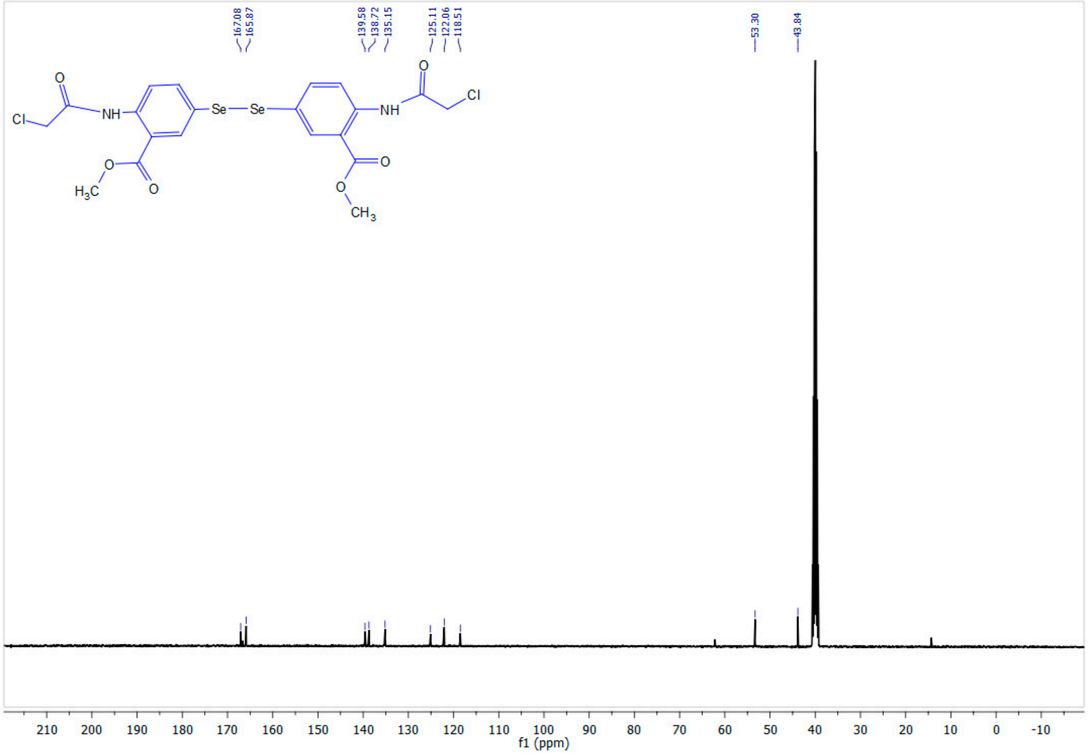

Figure S22. ^{13}CNMR chart of compound **9**

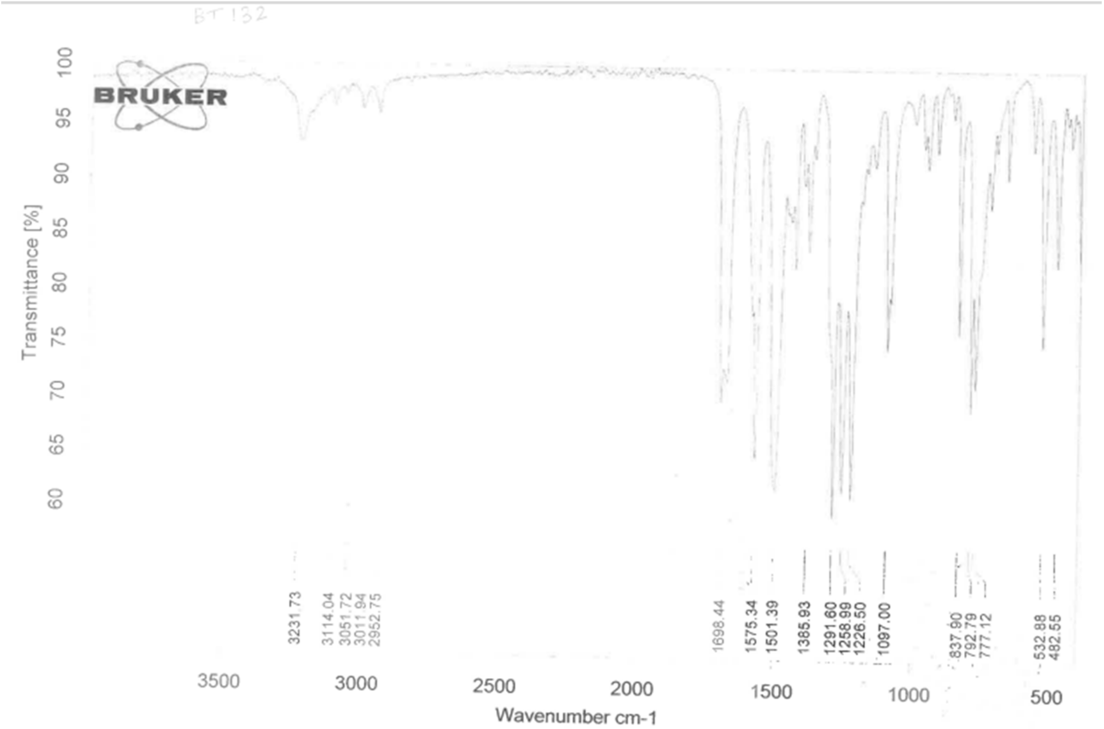

Figure S23. IR chart of compound **9**

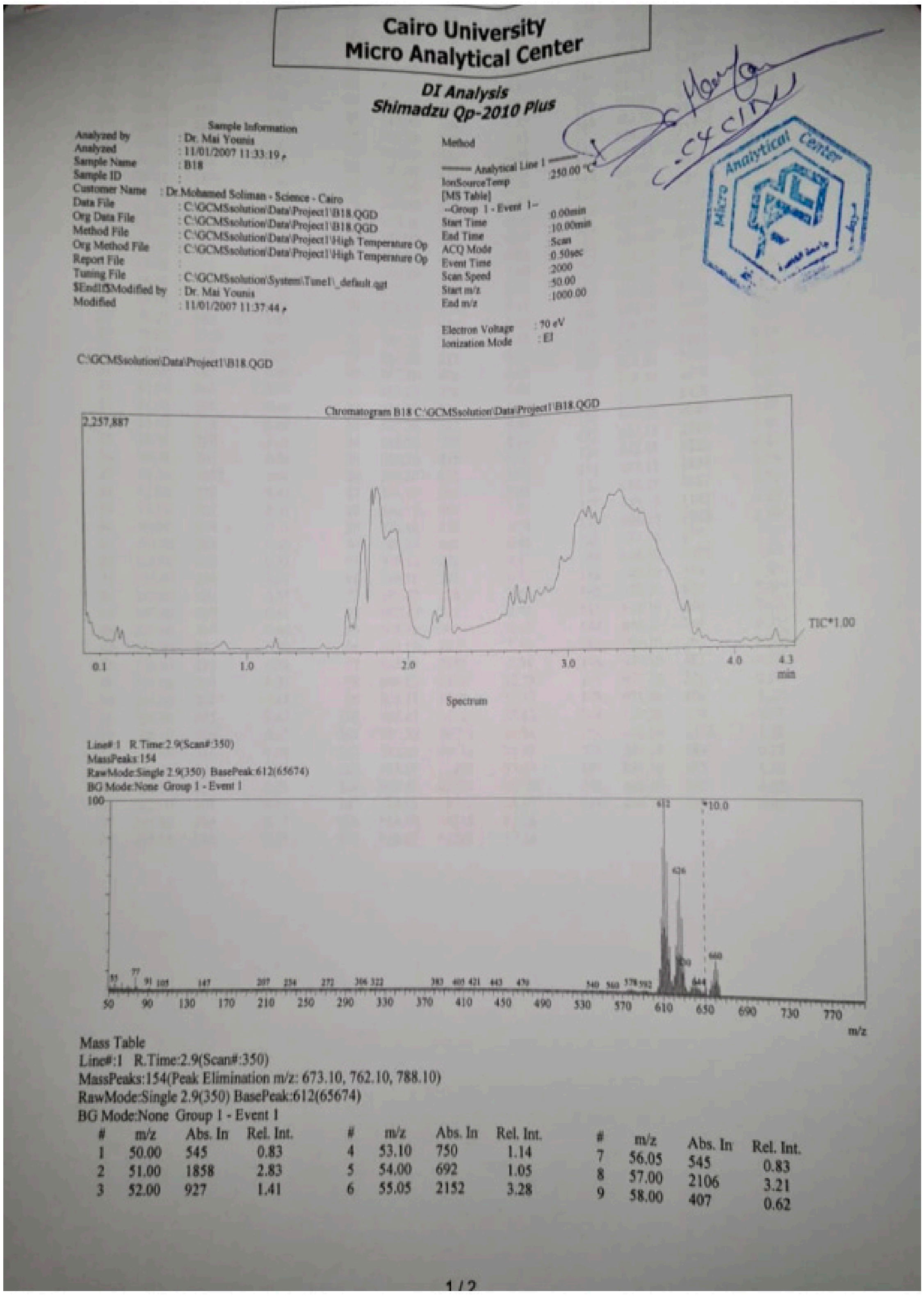

Cairo University
Micro Analytical Center

DI Analysis
Shimadzu Qp-2010 Plus

Sample Information

Analyzed by : Dr. Mai Younis
Analyzed : 11/01/2007 11:33:19 م
Sample Name : B18
Sample ID :
Customer Name : Dr.Mohamed Soliman - Science - Cairo
Data File : C:\GCMSsolution\Data\Project1\B18.QGD
Org Data File : C:\GCMSsolution\Data\Project1\B18.QGD
Method File : C:\GCMSsolution\Data\Project1\High Temperature Op
Org Method File : C:\GCMSsolution\Data\Project1\High Temperature Op
Report File :
Tuning File : C:\GCMSsolution\System\Tune1_default.qgt
$End1$Modified by : Dr. Mai Younis
Modified : 11/01/2007 11:37:44 م

Method

—— Analytical Line 1 ——
IonSourceTemp :250.00 °C
[MS Table]
--Group 1 - Event 1--
Start Time :0.00min
End Time :10.00min
ACQ Mode :Scan
Event Time :0.50sec
Scan Speed :2000
Start m/z :50.00
End m/z :1000.00

Electron Voltage : 70 eV
Ionization Mode : EI

C:\GCMSsolution\Data\Project1\B18.QGD

Mass Table
Line#:1 R.Time:2.9(Scan#:350)
MassPeaks:154(Peak Elimination m/z: 673.10, 762.10, 788.10)
RawMode:Single 2.9(350) BasePeak:612(65674)
BG Mode:None Group 1 - Event 1

#	m/z	Abs. In	Rel. Int.	#	m/z	Abs. In	Rel. Int.	#	m/z	Abs. In	Rel. Int.
1	50.00	545	0.83	4	53.10	750	1.14	7	56.05	545	0.83
2	51.00	1858	2.83	5	54.00	692	1.05	8	57.00	2106	3.21
3	52.00	927	1.41	6	55.05	2152	3.28	9	58.00	407	0.62

1 / 2

Figure S24. Mass chart of compound **9**

#	m/z	Abs. In	Rel. Int.
10	59.05	954	1.45
11	60.05	1042	1.59
12	61.10	380	0.58
13	61.95	614	0.93
14	63.05	2356	3.59
15	64.10	710	1.08
16	65.10	558	0.85
17	66.10	282	0.43
18	67.15	803	1.22
19	68.10	415	0.63
20	69.10	1537	2.34
21	70.10	551	0.84
22	71.10	1018	1.55
23	73.05	824	1.25
24	74.00	465	0.71
25	75.00	278	0.42
26	76.05	703	1.07
27	77.05	4277	6.51
28	78.05	980	1.49
29	79.05	1359	2.07
30	80.00	284	0.43
31	81.00	262	0.40
32	83.00	462	0.70
33	84.00	286	0.44
34	85.00	274	0.42
35	89.00	209	0.32
36	90.00	250	0.38
37	91.00	1057	1.61
38	92.00	278	0.42
39	93.00	223	0.34
40	99.00	204	0.31
41	105.00	284	0.43
42	115.00	210	0.32
43	125.00	204	0.31
44	147.00	206	0.31
45	207.00	407	0.62
46	209.00	300	0.46
47	212.00	527	0.80
48	234.00	223	0.34
49	272.00	209	0.32
50	304.00	286	0.44
51	306.00	415	0.63
52	308.00	426	0.65
53	320.00	313	0.48
54	322.00	276	0.42
55	326.00	204	0.31
56	383.00	305	0.46
57	391.00	206	0.31
58	405.00	230	0.35

#	m/z	Abs. In	Rel. Int.
59	407.00	273	0.42
60	421.00	374	0.57
61	435.00	201	0.31
62	443.00	215	0.33
63	470.00	292	0.44
64	472.00	268	0.41
65	540.00	218	0.33
66	544.00	215	0.33
67	548.00	354	0.54
68	550.00	210	0.32
69	552.00	255	0.39
70	560.00	246	0.37
71	562.00	231	0.35
72	574.00	569	0.87
73	575.00	402	0.61
74	576.10	1110	1.69
75	577.00	353	0.54
76	578.00	1385	2.11
77	579.20	434	0.66
78	580.20	778	1.18
79	581.20	217	0.33
80	582.20	436	0.66
81	583.20	278	0.42
82	585.20	234	0.36
83	587.20	327	0.50
84	588.20	327	0.50
85	589.20	415	0.63
86	590.20	673	1.02
87	591.10	295	0.45
88	592.15	860	1.31
89	593.10	378	0.58
90	594.10	602	0.92
91	595.10	276	0.42
92	596.10	281	0.43
93	597.10	218	0.33
94	602.10	346	0.53
95	603.10	295	0.45
96	604.10	1833	2.79
97	605.15	2195	3.34
98	606.15	8221	12.52
99	607.15	8098	12.33
100	608.10	25362	38.62
101	609.15	19573	29.80
102	610.10	49014	74.63
103	611.15	21892	33.33
104	612.10	65674	100.00
105	613.15	18961	28.87
106	614.10	44318	67.48
107	615.15	11267	17.16

#	m/z	Abs. In	Rel. Int.
108	616.10	15687	23.89
109	617.10	3929	5.98
110	618.15	4328	6.59
111	619.15	2547	3.88
112	620.15	5955	9.07
113	621.15	5755	8.76
114	622.15	16067	24.46
115	623.15	12409	18.89
116	624.15	31335	47.71
117	625.15	13327	20.29
118	626.15	39401	59.99
119	627.15	12515	19.06
120	628.15	25447	38.75
121	629.10	6876	10.47
122	630.15	8710	13.26
123	631.15	2531	3.85
124	632.15	2020	3.08
125	633.20	815	1.24
126	634.15	1129	1.72
127	635.15	1040	1.58
128	636.20	2655	4.04
129	637.20	2030	3.09
130	638.15	4478	6.82
131	639.15	2428	3.70
132	640.20	6089	9.27
133	641.15	2269	3.45
134	642.15	4222	6.43
135	643.15	1834	2.79
136	644.15	2467	3.76
137	645.15	1182	1.80
138	646.10	1898	2.89
139	647.15	677	1.03
140	648.10	1182	1.80
141	649.10	454	0.69
142	650.10	526	0.80
143	651.10	260	0.40
144	654.10	201	0.31
145	655.10	202	0.31
146	656.10	481	0.73
147	657.10	326	0.50
148	658.20	833	1.27
149	659.20	377	0.57
150	660.20	1118	1.70
151	661.10	494	0.75
152	662.10	855	1.30
153	663.10	343	0.52
154	664.10	310	0.47

Mass chart of compound **9**

*dimethyl 5,5'-diselanediylbis(2-(2-phenoxyacetamido) benzoate) (**10**).*

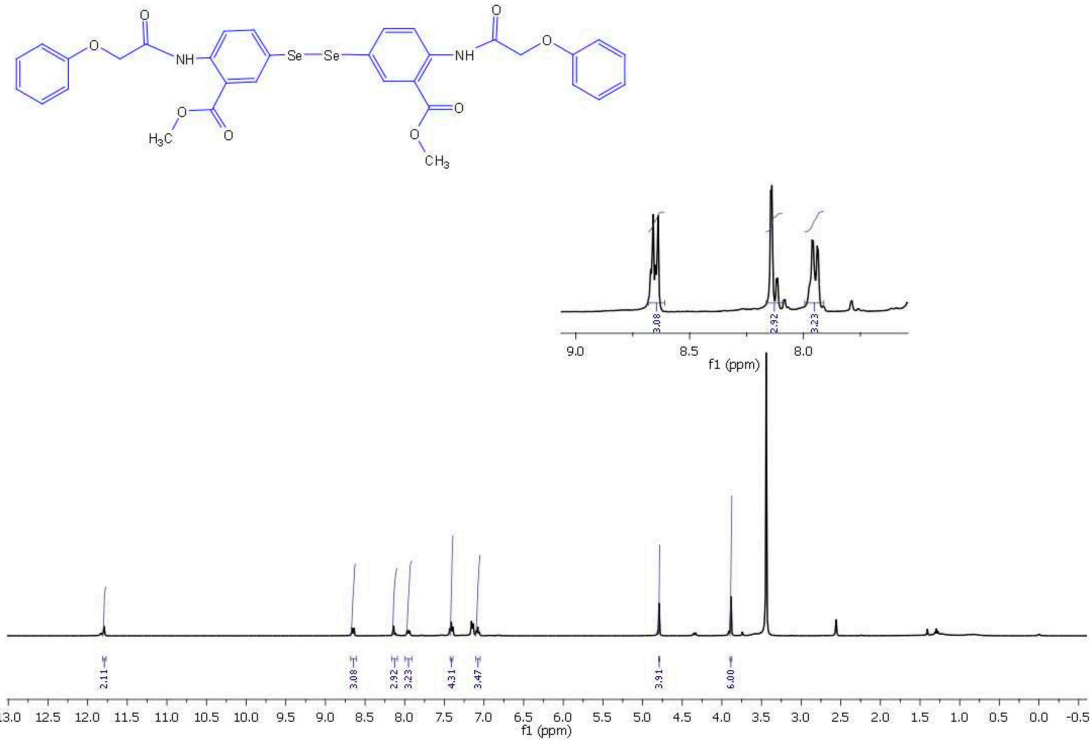

Figure S25. ^{1}HNMR chart of compound **10**

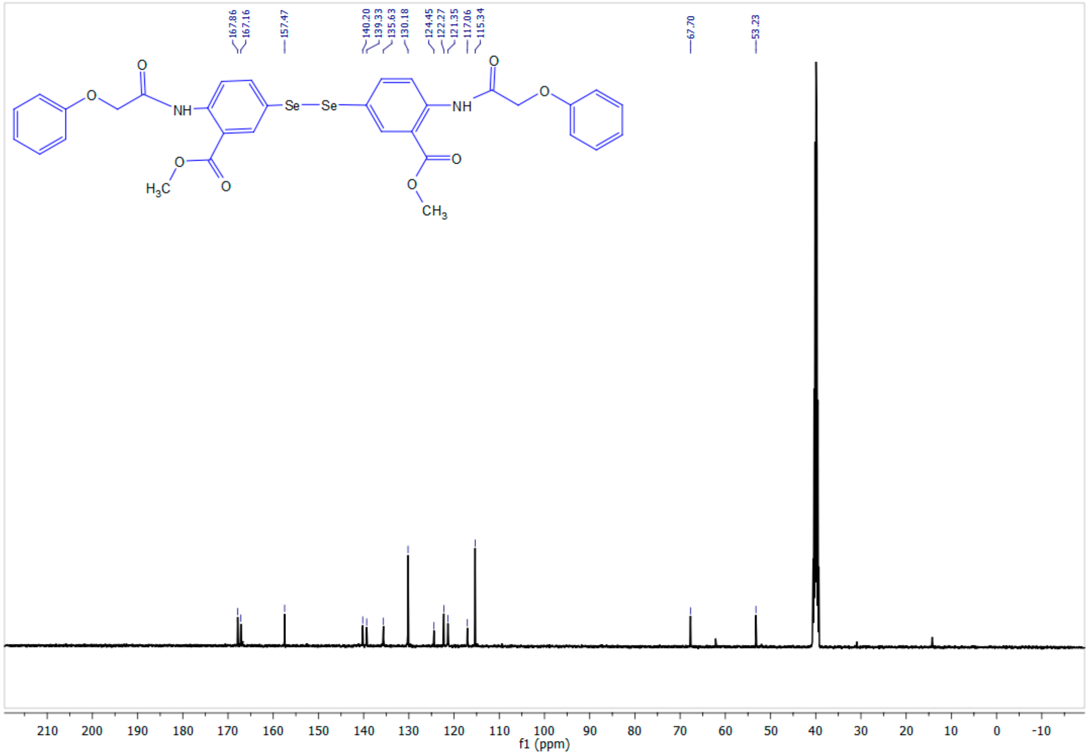

Figure S26. ^{13}CNMR chart of compound **10**

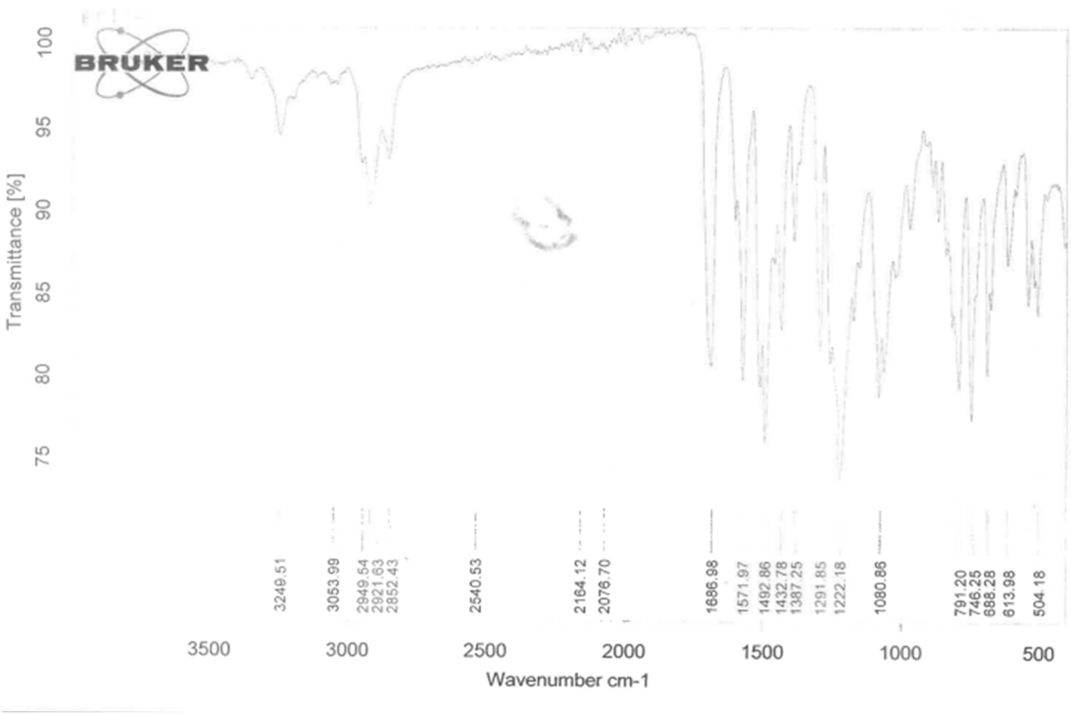

Figure S27. IR chart of compound **10**

Cairo University
Micro Analytical Center

DI Analysis
Shimadzu Qp-2010 Plus

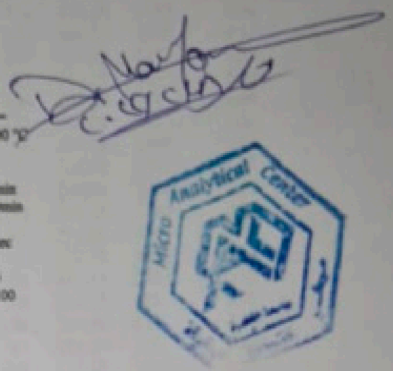

Sample Information		Method	
Analyzed by	: Dr. Mai Younis	——— Analytical Line 1 ———	
Analyzed	: 10/01/2007 01:52:05 ص	IonSourceTemp	:250.00 °C
Sample Name	: B19	[MS Table]	
Sample ID	:	--Group 1 - Event 1--	
Customer Name	: Dr.Mohamed Soliman - Science - Cairo	Start Time	:0.00min
Data File	: C:\GCMSsolution\Data\Project1\B19.QGD	End Time	:10.00min
Org Data File	: C:\GCMSsolution\Data\Project1\B19.QGD	ACQ Mode	:Scan
Method File	: C:\GCMSsolution\Data\Project1\High Temperature Op	Event Time	:0.50sec
Org Method File	: C:\GCMSsolution\Data\Project1\High Temperature Op	Scan Speed	:2000
Report File	:	Start m/z	:50.00
Tuning File	: C:\GCMSsolution\System\Tune1_default.qgt	End m/z	:1000.00
$EndIf$Modified by	: Dr. Mai Younis		
Modified	: 10/01/2007 01:56:55 ص		
		Electron Voltage	: 70 eV
		Ionization Mode	: EI

C:\GCMSsolution\Data\Project1\B19.QGD

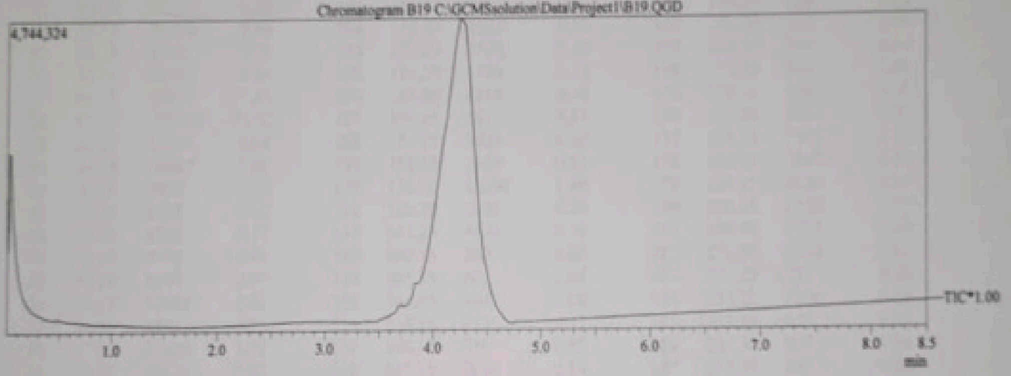

Spectrum

Line#:1 R.Time:4.3(Scan#:519)
MassPeaks:273
RawMode:Single 4.3(519) BasePeak:59(1235801)
BG Mode:None Group 1 - Event 1

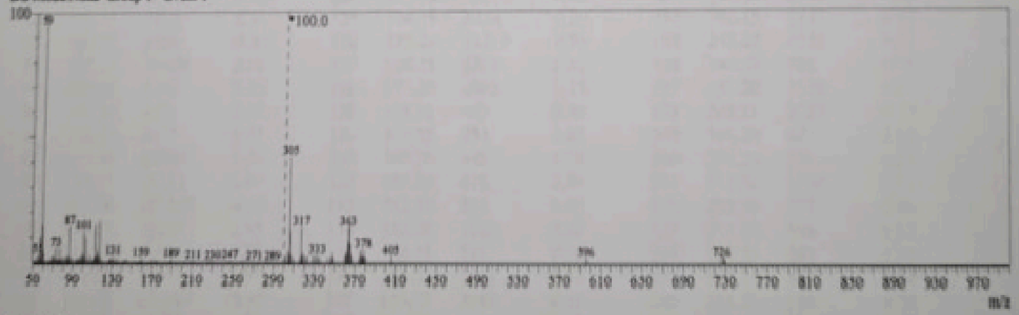

Mass Table
Line#:1 R.Time:4.3(Scan#:519)
MassPeaks:273
RawMode:Single 4.3(519) BasePeak:59(1235801)
BG Mode:None Group 1 - Event 1

#	m/z	Abs. In	Rel. Int.	#	m/z	Abs. In	Rel. Int.	#	m/z	Abs. In	Rel. Int.
1	50.15	2698	0.22	4	53.10	3495	0.28	7	56.15	19746	1.60
2	51.05	9293	0.75	5	54.15	5683	0.46	8	57.10	123422	9.99
3	52.05	2888	0.23	6	55.10	37022	3.00	9	58.15	183707	14.87

1 / 3

Figure S28. Mass chart of compound **10**

#	m/z	Abs. In	Rel. Int.	#	m/z	Abs. In	Rel. Int.	#	m/z	Abs. In	Rel. Int.
10	59.10	123580	100.00	79	128.25	9913	0.80	148	197.15	1106	0.09
11	60.05	51056	4.13	80	129.20	12753	1.03	149	198.05	3266	0.26
12	61.10	9529	0.77	81	130.25	6350	0.51	150	199.20	1182	0.10
13	62.15	1694	0.14	82	131.20	27886	2.26	151	200.15	1450	0.12
14	63.10	4455	0.36	83	132.25	5385	0.44	152	201.25	5737	0.46
15	64.15	2465	0.20	84	133.20	8585	0.69	153	202.20	1385	0.11
16	65.10	7606	0.62	85	134.25	2900	0.23	154	203.25	2177	0.18
17	66.15	3107	0.25	86	135.25	16736	1.35	155	204.25	591	0.05
18	67.10	8836	0.72	87	136.25	1396	0.11	156	205.30	774	0.06
19	68.15	5741	0.46	88	137.20	1366	0.11	157	206.10	412	0.03
20	69.10	29596	2.39	89	138.25	929	0.08	158	207.05	1397	0.11
21	70.15	13485	1.09	90	139.20	3474	0.28	159	208.10	1767	0.14
22	71.10	35452	2.87	91	140.25	2026	0.16	160	209.05	3204	0.26
23	72.15	16940	1.37	92	141.20	4914	0.40	161	210.15	1494	0.12
24	73.10	68638	5.55	93	142.25	4078	0.33	162	211.05	5748	0.47
25	74.15	7886	0.64	94	143.20	18249	1.48	163	212.15	1930	0.16
26	75.15	16723	1.35	95	144.20	4407	0.36	164	213.15	2716	0.22
27	76.15	7868	0.64	96	145.20	7615	0.62	165	214.25	634	0.05
28	77.10	68041	5.51	97	146.25	2609	0.21	166	215.30	631	0.05
29	78.15	8611	0.70	98	147.20	2892	0.23	167	216.20	326	0.03
30	79.15	20331	1.65	99	148.20	695	0.06	168	217.25	1847	0.15
31	80.15	4425	0.36	100	149.20	524	0.04	169	218.20	431	0.03
32	81.10	18788	1.52	101	150.25	768	0.06	170	219.25	888	0.07
33	82.15	6220	0.50	102	151.15	3815	0.31	171	220.30	226	0.02
34	83.15	31274	2.53	103	152.20	1241	0.10	172	221.30	362	0.03
35	84.15	8991	0.73	104	153.25	2176	0.18	173	222.10	444	0.04
36	85.15	32933	2.66	105	154.25	1750	0.14	174	223.05	502	0.04
37	86.15	22687	1.84	106	155.20	4334	0.35	175	224.10	748	0.06
38	87.15	175790	14.22	107	156.15	1810	0.15	176	225.20	826	0.07
39	88.15	11668	0.94	108	157.25	3483	0.28	177	226.15	1951	0.16
40	89.15	19847	1.61	109	158.25	2669	0.22	178	227.25	2247	0.18
41	90.15	3460	0.28	110	159.20	18396	1.49	179	228.15	2010	0.16
42	91.10	8958	0.72	111	160.20	2502	0.20	180	229.15	2126	0.17
43	92.25	2326	0.19	112	161.20	4391	0.36	181	230.10	3233	0.26
44	93.15	4945	0.40	113	162.15	562	0.05	182	231.30	1500	0.12
45	94.20	6799	0.55	114	163.15	601	0.05	183	232.25	751	0.06
46	95.15	17605	1.42	115	164.05	1041	0.08	184	233.25	1206	0.10
47	96.25	6501	0.53	116	165.15	911	0.07	185	234.30	623	0.05
48	97.15	21616	1.75	117	166.15	916	0.07	186	235.30	537	0.04
49	98.25	10122	0.82	118	167.15	2014	0.16	187	237.30	343	0.03
50	99.20	43671	3.53	119	168.15	2058	0.17	188	238.20	457	0.04
51	100.25	31258	2.53	120	169.15	6783	0.55	189	239.20	786	0.06
52	101.15	153243	12.40	121	170.15	3140	0.25	190	240.15	1168	0.09
53	102.25	24631	1.99	122	171.20	4726	0.38	191	241.15	1605	0.13
54	103.20	126102	10.20	123	172.25	2644	0.21	192	242.15	1190	0.10
55	104.20	9614	0.78	124	173.25	5320	0.43	193	243.15	2822	0.23
56	105.15	3811	0.31	125	174.35	2414	0.20	194	244.15	1111	0.09
57	106.25	4406	0.36	126	175.25	11526	0.93	195	245.20	1655	0.13
58	107.15	31609	2.56	127	176.25	1872	0.15	196	246.35	792	0.06
59	108.20	4266	0.35	128	177.20	1862	0.15	197	247.30	7518	0.61
60	109.20	4473	0.36	129	178.15	463	0.04	198	248.25	2307	0.19
61	110.25	3933	0.32	130	179.20	351	0.03	199	249.20	607	0.05
62	111.20	20081	1.62	131	180.20	449	0.04	200	250.20	201	0.02
63	112.25	23111	1.87	132	181.20	618	0.05	201	251.30	1330	0.11
64	113.20	181367	14.68	133	182.10	582	0.05	202	252.30	372	0.03
65	114.25	32131	2.60	134	183.20	1009	0.08	203	253.30	346	0.03
66	115.20	56626	4.58	135	184.15	771	0.06	204	254.30	282	0.02
67	116.25	32977	2.67	136	185.20	1758	0.14	205	255.15	530	0.04
68	117.20	203367	16.46	137	186.25	2042	0.17	206	256.20	398	0.03
69	118.20	16386	1.33	138	187.20	2170	0.18	207	257.20	1065	0.09
70	119.20	7193	0.58	139	188.25	1449	0.12	208	258.35	802	0.06
71	120.20	1370	0.11	140	189.25	13930	1.13	209	259.30	5948	0.48
72	121.25	1279	0.10	141	190.15	2482	0.20	210	260.25	1128	0.09
73	122.20	641	0.05	142	191.25	1682	0.14	211	261.30	1122	0.09
74	123.20	1988	0.16	143	192.35	657	0.05	212	262.30	423	0.03
75	124.25	935	0.08	144	193.30	4294	0.35	213	263.30	452	0.04
76	125.25	3399	0.28	145	194.25	982	0.08	214	265.30	354	0.03
77	126.25	5894	0.48	146	195.05	1093	0.09	215	269.30	262	0.02
78	127.25	20610	1.67	147	196.05	1690	0.14	216	270.30	220	0.02

2 / 3

Mass chart of compound **10**

#	m/z	Abs. In	Rel. Int.	#	m/z	Abs. In	Rel. Int.	#	m/z	Abs. In	Rel. Int.
217	271.30	897	0.07	236	305.40	5145	0.42	255	361.35	496	0.04
218	272.30	358	0.03	237	306.35	1560	0.13	256	362.30	1012	0.08
219	273.40	642	0.05	238	307.40	399	0.03	257	363.35	1701	0.14
220	274.40	302	0.02	239	309.40	244	0.02	258	364.30	2038	0.16
221	275.40	406	0.03	240	316.40	233	0.02	259	365.25	907	0.07
222	276.40	212	0.02	241	317.40	1713	0.14	260	366.30	455	0.04
223	277.20	591	0.05	242	318.40	458	0.04	261	367.30	238	0.02
224	283.20	225	0.02	243	319.40	343	0.03	262	374.30	204	0.02
225	285.40	577	0.05	244	321.40	233	0.02	263	375.50	628	0.05
226	286.40	340	0.03	245	323.40	308	0.02	264	376.50	380	0.03
227	287.40	542	0.04	246	329.40	319	0.03	265	377.50	298	0.02
228	288.40	244	0.02	247	331.40	287	0.02	266	378.40	642	0.05
229	289.35	635	0.05	248	333.40	338	0.03	267	379.40	314	0.03
230	290.30	287	0.02	249	335.40	244	0.02	268	380.40	204	0.02
231	291.30	382	0.03	250	343.40	202	0.02	269	405.40	286	0.02
232	293.30	308	0.02	251	346.40	247	0.02	270	596.40	217	0.02
233	301.30	255	0.02	252	347.40	462	0.04	271	726.40	231	0.02
234	303.45	574	0.05	253	348.40	247	0.02	272	728.40	281	0.02
235	304.40	390	0.03	254	360.40	358	0.03	273	729.40	209	0.02

3 / 3

Mass chart of compound **10**

*4,4'-((diselanediylbis(2-(methoxycarbonyl)-4,1-phenylene)) bis(azanediyl)) bis (4-oxobutanoic acid (**11**).*

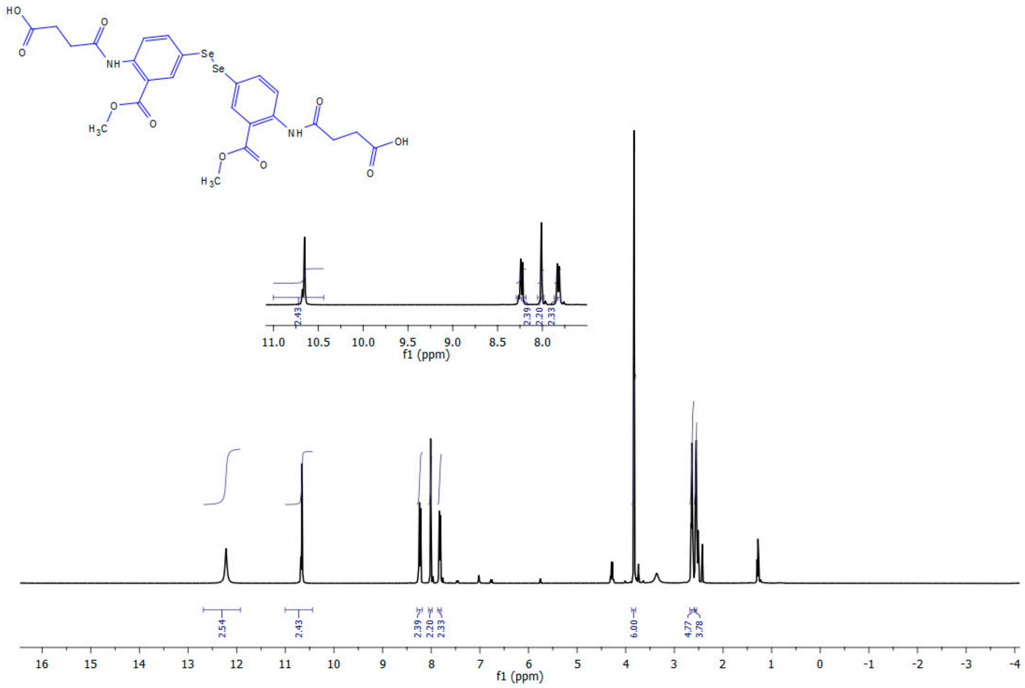

Figure S29. ^{1}HNMR chart of compound **11**

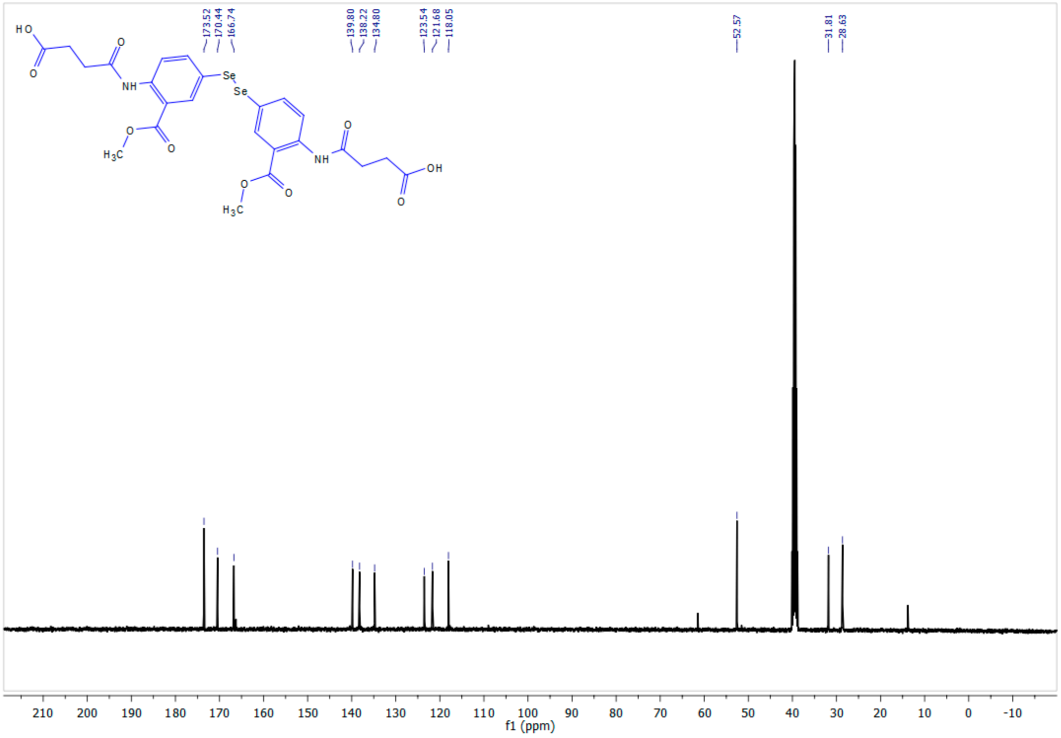

Figure S30. ^{13}CNMR chart of compound **11**

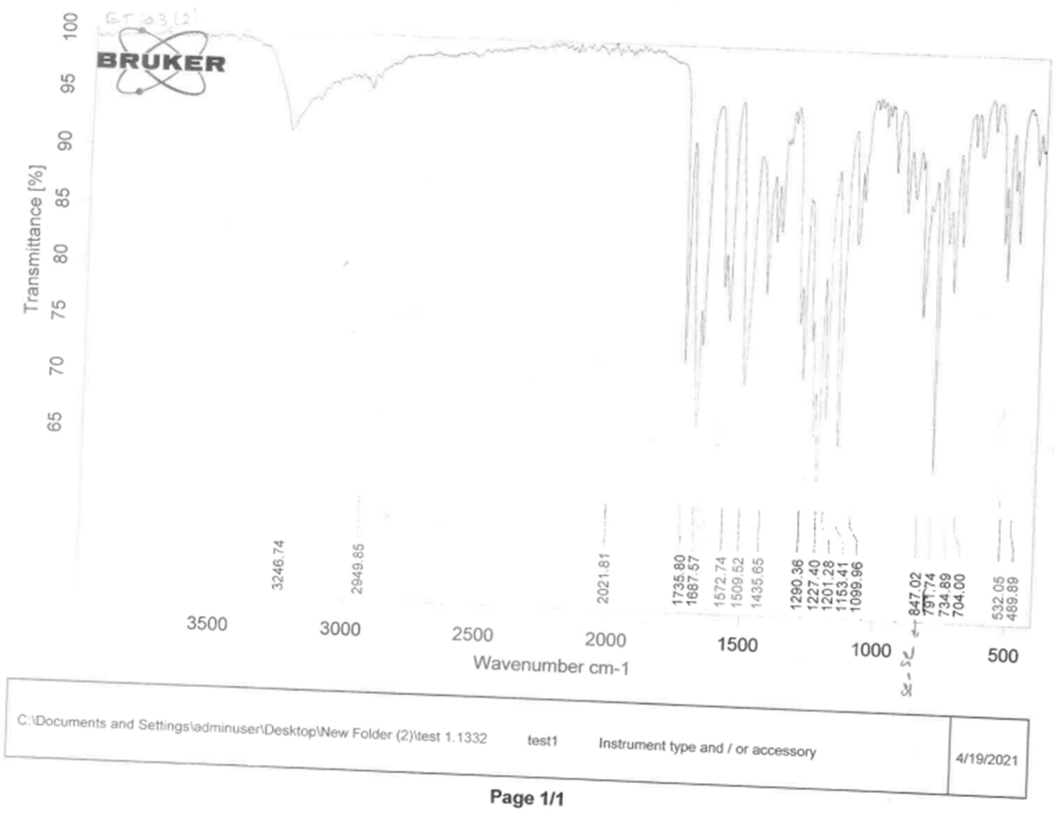

Figure S31. IR chart of compound **11**

Cairo University
Micro Analytical Center

DI Analysis
Shimadzu Qp-2010 Plus

Sample Information	
Analyzed by	: Dr. Mai Younis
Analyzed	: 03/01/2007 07:05:56 ,
Sample Name	: B4
Sample ID	:
Customer Name	: Dr.Mohamed Soliman - Science - Cairo
Data File	: C:\GCMSsolution\Data\Project1\B4.QGD
Org Data File	: C:\GCMSsolution\Data\Project1\B4.QGD
Method File	: C:\GCMSsolution\Data\Project1\High Temperature Op
Org Method File	: C:\GCMSsolution\Data\Project1\High Temperature Op
Report File	:
Tuning File	: C:\GCMSsolution\System\Tune1_default.qgt
$EndIf$Modified by	: Dr. Mai Younis
Modified	: 03/01/2007 07:10:40 ,

Method

——— Analytical Line 1 ———

IonSourceTemp	:250.00 °C
[MS Table]	
--Group 1 - Event 1--	
Start Time	:0.00min
End Time	:10.00min
ACQ Mode	:Scan
Event Time	:0.50sec
Scan Speed	:1000
Start m/z	:50.00
End m/z	:510.00
Electron Voltage	: 70 eV
Ionization Mode	: EI

C:\GCMSsolution\Data\Project1\B4.QGD

Chromatogram B4 C:\GCMSsolution\Data\Project1\B4.QGD

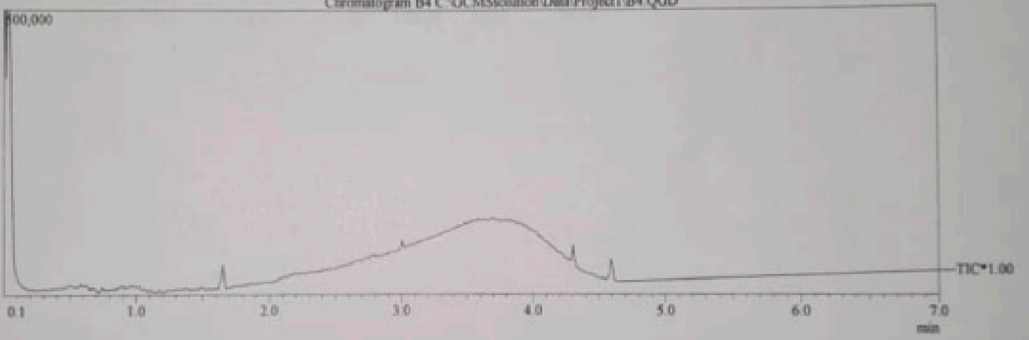

Spectrum

Line#:1 R.Time:2.4(Scan#:293)
MassPeaks:82
RawMode:Single 2.4(293) BasePeak:91(3702)
BG Mode:None Group 1 - Event 1

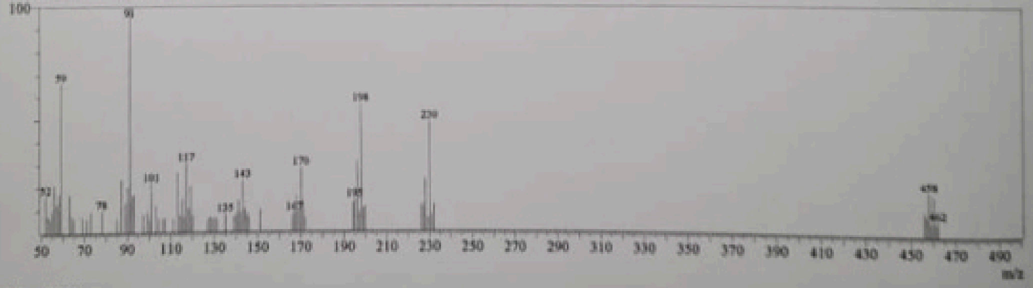

Mass Table
Line#:1 R.Time:2.4(Scan#:293)
MassPeaks:82
RawMode:Single 2.4(293) BasePeak:91(3702)
BG Mode:None Group 1 - Event 1

#	m/z	Abs. In	Rel. Int.	#	m/z	Abs. In	Rel. Int.	#	m/z	Abs. In	Rel. Int.
1	52.00	598	16.15	4	55.05	361	9.75	7	58.15	626	16.91
2	53.00	262	7.08	5	56.00	780	21.07	8	59.05	2447	66.10
3	54.00	220	5.94	6	57.10	463	12.51	9	63.00	620	16.75

1 / 2

Figure S32. Mass chart of compound **11**

#	m/z	Abs. In	Rel. Int.
10	64.00	270	7.29
11	65.00	234	6.32
12	69.00	254	6.86
13	71.00	220	5.94
14	73.00	335	9.05
15	78.00	354	9.56
16	85.00	222	6.00
17	87.15	873	23.58
18	89.10	486	13.13
19	90.15	748	20.21
20	91.15	3702	100.00
21	92.20	594	16.05
22	93.05	632	17.07
23	97.10	292	7.89
24	99.10	335	9.05
25	100.10	218	5.89
26	101.10	798	21.56
27	103.20	449	12.13
28	104.20	231	6.24
29	106.20	214	5.78
30	107.20	233	6.29
31	111.20	206	5.56
32	113.15	978	26.42
33	114.20	276	7.46
34	115.15	543	14.67

#	m/z	Abs. In	Rel. Int.
35	116.10	262	7.08
36	117.10	1126	30.42
37	118.10	388	10.48
38	119.10	758	20.48
39	120.10	289	7.81
40	127.10	217	5.86
41	128.10	244	6.59
42	129.10	214	5.78
43	130.10	247	6.67
44	131.10	212	5.73
45	135.10	287	7.75
46	139.10	258	6.97
47	140.10	303	8.18
48	141.00	505	13.64
49	142.00	314	8.48
50	143.05	840	22.69
51	144.00	372	10.05
52	145.00	313	8.45
53	146.00	249	6.73
54	151.00	358	9.67
55	166.00	284	7.67
56	167.00	297	8.02
57	168.00	606	16.37
58	169.05	320	8.64
59	170.05	1038	28.04

#	m/z	Abs. In	Rel. Int.
60	170.95	510	13.78
61	172.00	222	6.00
62	194.05	462	12.48
63	195.05	505	13.64
64	196.05	1148	31.01
65	197.05	314	8.48
66	198.00	2086	56.35
67	198.95	381	10.29
68	199.90	409	11.05
69	226.15	416	11.24
70	227.10	439	11.86
71	228.10	846	22.85
72	229.10	215	5.81
73	230.05	1796	48.51
74	231.10	278	7.51
75	232.10	430	11.62
76	456.20	398	10.75
77	457.20	319	8.62
78	458.25	706	19.07
79	459.20	209	5.65
80	460.20	654	17.67
81	461.20	204	5.51
82	462.20	249	6.73

2 / 2

Mass chart of compound **11**

*Dimethyl 5,5'-diselanediylbis(2-(2,5-dioxopyrrolidin-1-yl) benzoate (**12**).*

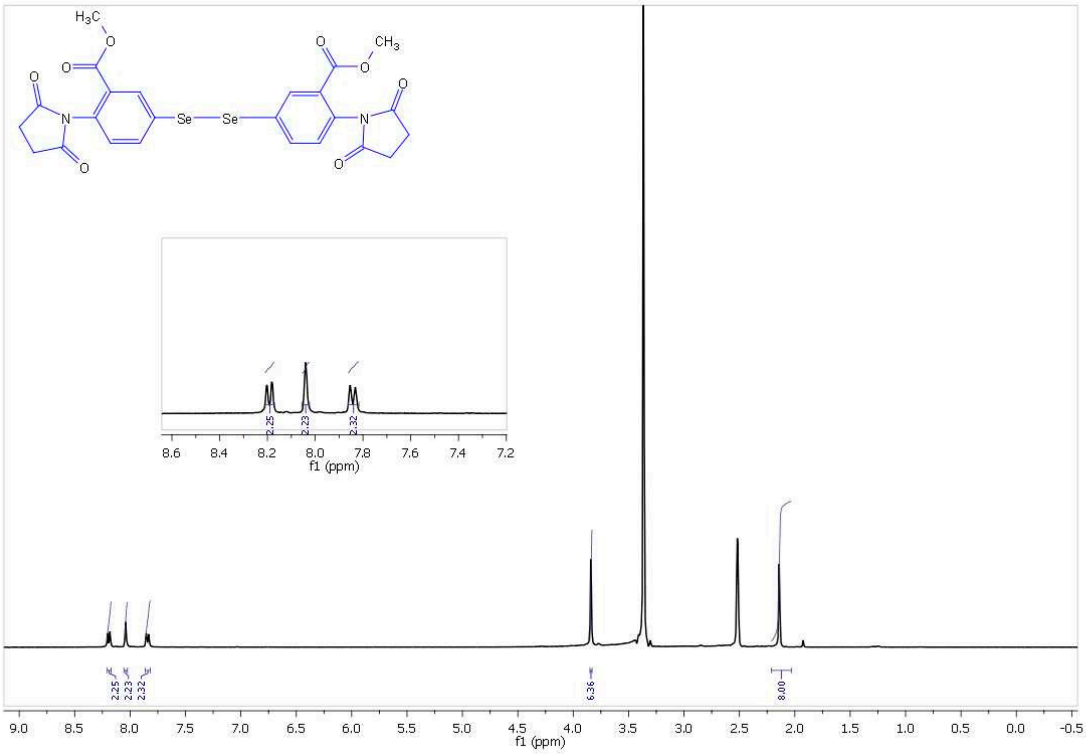

Figure S33. ^{1}HNMR chart of compound **12**

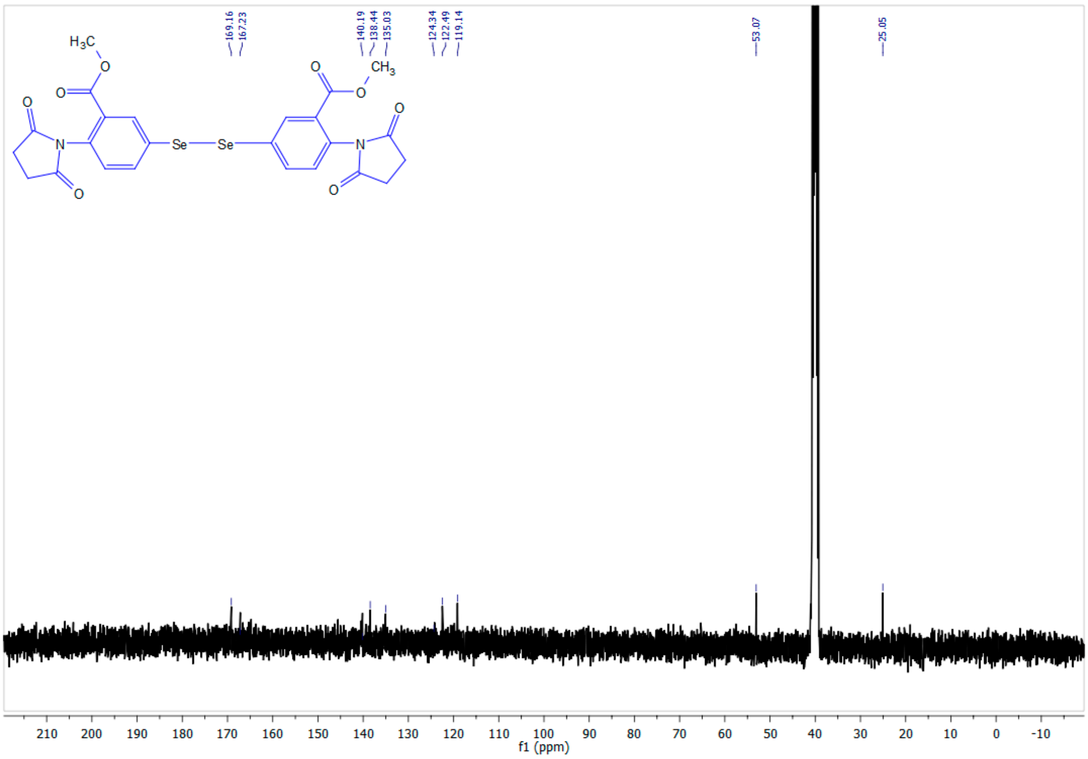

Figure S34. ^{13}CNMR chart of compound **12**

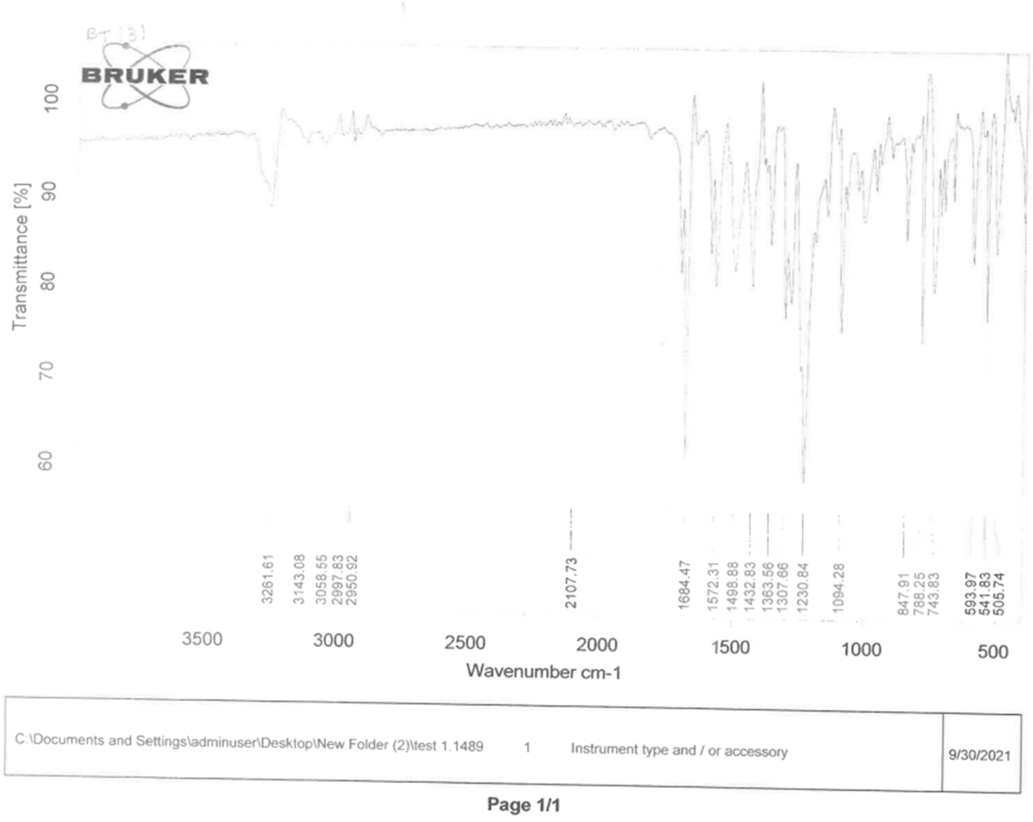

Figure S35. IR chart of compound **12**

Cairo University
Micro Analytical Center

DI Analysis
Shimadzu Qp-2010 Plus

Sample Information

Analyzed by	: Dr. Mai Younis
Analyzed	: 10/01/2007 02:06:32 ص
Sample Name	: B30
Sample ID	:
Customer Name	: Dr Mohamed Soliman - Science - Cairo
Data File	: C:\GCMSsolution\Data\Project1\B30.QGD
Org Data File	: C:\GCMSsolution\Data\Project1\B30.QGD
Method File	: C:\GCMSsolution\Data\Project1\High Temperature Op
Org Method File	: C:\GCMSsolution\Data\Project1\High Temperature Op
Report File	:
Tuning File	: C:\GCMSsolution\System\Tune1_default.qgt
$EndIf$Modified by	: Dr. Mai Younis
Modified	: 10/01/2007 02:12:57 ص

Method

—— Analytical Line 1 ——

IonSourceTemp	:250.00 °C
[MS Table]	
--Group 1 - Event 1--	
Start Time	:0.00min
End Time	:10.00min
ACQ Mode	:Scan
Event Time	:0.50sec
Scan Speed	:2000
Start m/z	:50.00
End m/z	:1000.00
Electron Voltage	: 70 eV
Ionization Mode	: EI

C:\GCMSsolution\Data\Project1\B30.QGD

Chromatogram B30 C:\GCMSsolution\Data\Project1\B30.QGD

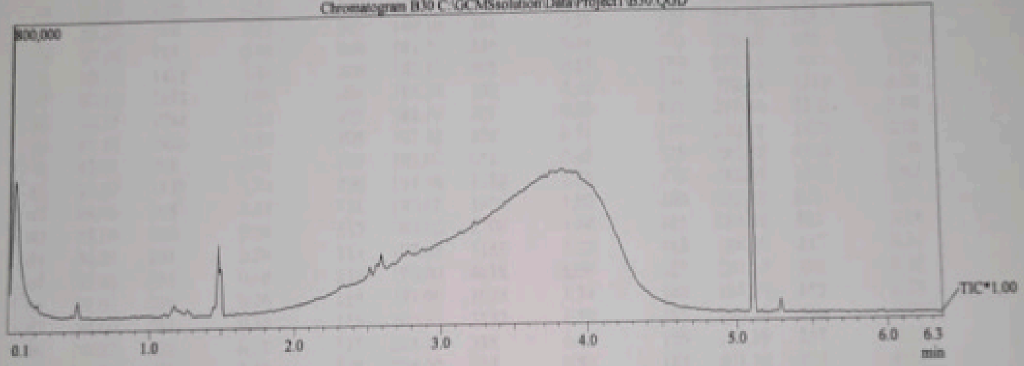

Spectrum

Line#:1 R.Time:3.8(Scan#:452)
MassPeaks:265
RawMode:Single 3.8(452) BasePeak:55(76957)
BG Mode:None Group 1 - Event 1

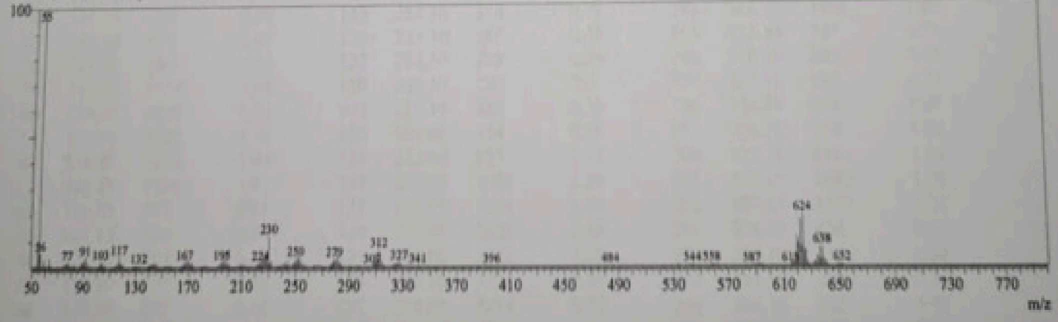

Mass Table
Line#:1 R.Time:3.8(Scan#:452)
MassPeaks:265
RawMode:Single 3.8(452) BasePeak:55(76957)
BG Mode:None Group 1 - Event 1

#	m/z	Abs. In	Rel. Int.	#	m/z	Abs. In	Rel. Int.	#	m/z	Abs. In	Rel. Int.
1	50.00	295	0.38	4	53.15	727	0.94	7	56.05	4335	5.63
2	51.00	596	0.77	5	54.15	6098	7.92	8	57.10	753	0.98
3	52.00	551	0.72	6	55.05	76957	100.00	9	58.10	346	0.45

Figure S36. Mass chart of compound **12**

#	m/z	Abs. In	Rel. Int.	#	m/z	Abs. In	Rel. Int.	#	m/z	Abs. In	Rel. Int.
10	59.05	2361	3.07	79	144.15	948	1.23	148	241.15	534	0.69
11	60.05	769	1.00	80	145.10	1273	1.65	149	242.10	1250	1.62
12	61.10	284	0.37	81	146.15	770	1.00	150	243.10	390	0.51
13	62.15	847	1.10	82	148.10	226	0.29	151	244.10	1682	2.19
14	63.10	2718	3.53	83	151.10	551	0.72	152	245.10	369	0.48
15	64.10	796	1.03	84	152.10	236	0.31	153	246.10	542	0.70
16	65.10	623	0.81	85	153.10	394	0.51	154	247.10	399	0.52
17	66.10	353	0.46	86	154.10	369	0.48	155	248.10	938	1.22
18	67.10	374	0.49	87	155.10	340	0.44	156	249.05	1283	1.67
19	69.10	495	0.64	88	156.10	537	0.70	157	250.10	2476	3.22
20	70.10	338	0.44	89	157.10	406	0.53	158	251.15	1626	2.11
21	71.10	439	0.57	90	160.10	297	0.39	159	252.10	3532	4.59
22	73.20	650	0.84	91	164.10	234	0.30	160	253.05	2638	3.43
23	74.20	556	0.72	92	165.10	548	0.71	161	254.00	1634	2.12
24	75.10	860	1.12	93	166.05	806	1.05	162	255.05	588	0.76
25	76.10	1014	1.32	94	167.00	1514	1.97	163	256.15	586	0.76
26	77.10	1638	2.13	95	168.05	1127	1.46	164	258.10	322	0.42
27	78.10	1244	1.62	96	169.00	2329	3.03	165	263.10	228	0.30
28	79.10	572	0.74	97	170.05	1484	1.93	166	264.10	262	0.34
29	80.10	590	0.77	98	171.00	1124	1.46	167	265.10	487	0.63
30	81.10	446	0.58	99	172.10	1340	1.74	168	266.10	266	0.35
31	82.10	874	1.14	100	173.10	929	1.21	169	267.10	633	0.82
32	83.05	682	0.89	101	174.10	346	0.45	170	268.10	322	0.42
33	84.10	410	0.53	102	178.10	265	0.34	171	269.10	202	0.26
34	85.10	359	0.47	103	180.10	284	0.37	172	275.10	226	0.29
35	87.10	737	0.96	104	181.10	335	0.44	173	276.10	348	0.45
36	88.10	1428	1.86	105	182.10	406	0.53	174	277.15	1017	1.32
37	89.10	1534	1.99	106	183.10	303	0.39	175	278.15	1615	2.10
38	90.15	1794	2.33	107	184.10	527	0.68	176	279.10	2262	2.94
39	91.10	2926	3.80	108	192.10	390	0.51	177	280.15	1661	2.16
40	92.05	704	0.91	109	193.00	351	0.46	178	281.10	4306	5.60
41	93.05	1337	1.74	110	194.00	1180	1.53	179	282.05	1565	2.03
42	94.00	268	0.35	111	195.05	1476	1.92	180	283.15	821	1.07
43	95.00	506	0.66	112	196.00	2799	3.64	181	284.10	881	1.14
44	96.00	201	0.26	113	197.05	1155	1.50	182	286.10	247	0.32
45	97.00	354	0.46	114	198.00	4618	6.00	183	290.10	201	0.26
46	98.00	201	0.26	115	199.00	1033	1.34	184	294.10	215	0.28
47	99.00	233	0.30	116	200.05	1152	1.50	185	296.10	226	0.29
48	100.00	238	0.31	117	201.00	385	0.50	186	298.10	257	0.33
49	101.00	330	0.43	118	204.00	234	0.30	187	307.10	274	0.36
50	102.25	730	0.95	119	206.00	239	0.31	188	308.15	1182	1.54
51	103.15	1742	2.26	120	207.00	300	0.39	189	309.15	1355	1.76
52	104.20	1383	1.80	121	208.00	337	0.44	190	310.15	3102	4.03
53	105.10	663	0.86	122	209.10	825	1.07	191	311.15	1824	2.37
54	106.10	506	0.66	123	210.10	361	0.47	192	312.15	4948	6.43
55	107.10	458	0.60	124	211.10	886	1.15	193	313.10	2505	3.26
56	111.10	226	0.29	125	212.10	314	0.41	194	314.10	1455	1.89
57	113.20	519	0.67	126	213.10	265	0.34	195	315.10	545	0.71
58	114.20	394	0.51	127	214.10	278	0.36	196	316.10	249	0.32
59	115.15	1136	1.48	128	216.10	236	0.31	197	322.10	252	0.33
60	116.15	1241	1.61	129	220.10	252	0.33	198	323.20	628	0.82
61	117.15	3180	4.13	130	221.00	454	0.59	199	324.20	718	0.93
62	118.10	1263	1.64	131	222.00	855	1.11	200	325.15	816	1.06
63	119.15	1086	1.41	132	223.05	1068	1.39	201	326.10	1060	1.38
64	120.10	703	0.91	133	223.95	1204	1.56	202	327.15	1320	1.72
65	121.10	226	0.29	134	225.05	2025	2.63	203	328.10	436	0.57
66	127.10	294	0.38	135	226.00	2782	3.62	204	329.10	412	0.54
67	128.10	294	0.38	136	227.10	2271	2.95	205	341.10	270	0.35
68	129.10	497	0.65	137	228.05	4414	5.74	206	396.10	212	0.28
69	130.10	482	0.63	138	229.15	955	1.24	207	484.10	223	0.29
70	131.10	473	0.61	139	230.05	8970	11.66	208	533.10	246	0.32
71	132.20	641	0.83	140	231.05	1386	1.80	209	534.10	217	0.28
72	133.20	566	0.74	141	232.05	1683	2.19	210	540.10	254	0.33
73	136.20	225	0.29	142	233.10	270	0.35	211	542.10	374	0.49
74	139.20	554	0.72	143	236.10	206	0.27	212	544.10	433	0.56
75	140.10	521	0.68	144	237.10	367	0.48	213	545.10	276	0.36
76	141.10	903	1.17	145	238.10	250	0.32	214	556.10	252	0.33
77	142.05	880	1.14	146	239.00	276	0.36	215	557.10	330	0.43
78	143.10	1281	1.66	147	240.05	904	1.17	216	558.10	521	0.68

2 / 3

Mass chart of compound **12**

#	m/z	Abs. In	Rel. Int.	#	m/z	Abs. In	Rel. Int.	#	m/z	Abs. In	Rel. Int.
217	559.10	444	0.58	234	617.15	1317	1.71	251	634.20	3026	3.93
218	560.10	386	0.50	235	618.20	3140	4.08	252	635.25	2389	3.10
219	561.10	442	0.57	236	619.25	2905	3.77	253	636.25	5588	7.26
220	562.10	348	0.45	237	620.20	8630	11.21	254	637.20	2172	2.82
221	563.10	326	0.42	238	621.20	6727	8.74	255	638.20	5702	7.41
222	566.10	202	0.26	239	622.20	13474	17.51	256	639.20	1810	2.35
223	587.10	239	0.31	240	623.20	5658	7.35	257	640.20	1945	2.53
224	588.10	202	0.26	241	624.20	14886	19.34	258	641.20	566	0.74
225	589.10	287	0.37	242	625.20	4374	5.68	259	642.20	327	0.42
226	590.10	318	0.41	243	626.20	4846	6.30	260	648.20	294	0.38
227	591.10	550	0.71	244	627.20	1631	2.12	261	649.20	425	0.55
228	592.10	244	0.32	245	628.20	578	0.75	262	650.20	466	0.61
229	593.20	690	0.90	246	629.20	281	0.37	263	651.20	258	0.34
230	594.20	249	0.32	247	630.20	300	0.39	264	652.20	598	0.78
231	595.20	326	0.42	248	631.20	550	0.71	265	653.20	214	0.28
232	615.20	217	0.28	249	632.25	1257	1.63				
233	616.25	961	1.25	250	633.25	1235	1.60				

Mass chart of compound **12**

(2Z,2'Z)-4,4'-((diselanediylbis(2-(methoxycarbonyl)-4,1-phenylene)) bis(azanediyl)) bis (4-oxobut-2-enoic acid) (13).

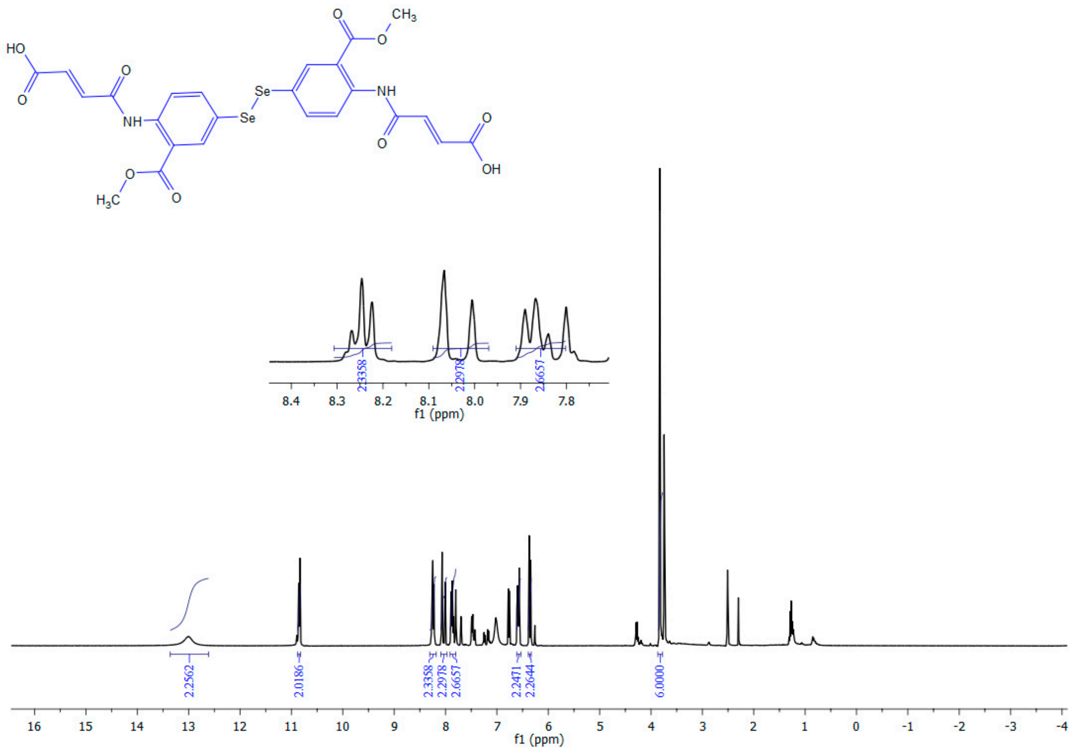

Figure S37. ^{1}HNMR chart of compound **13**

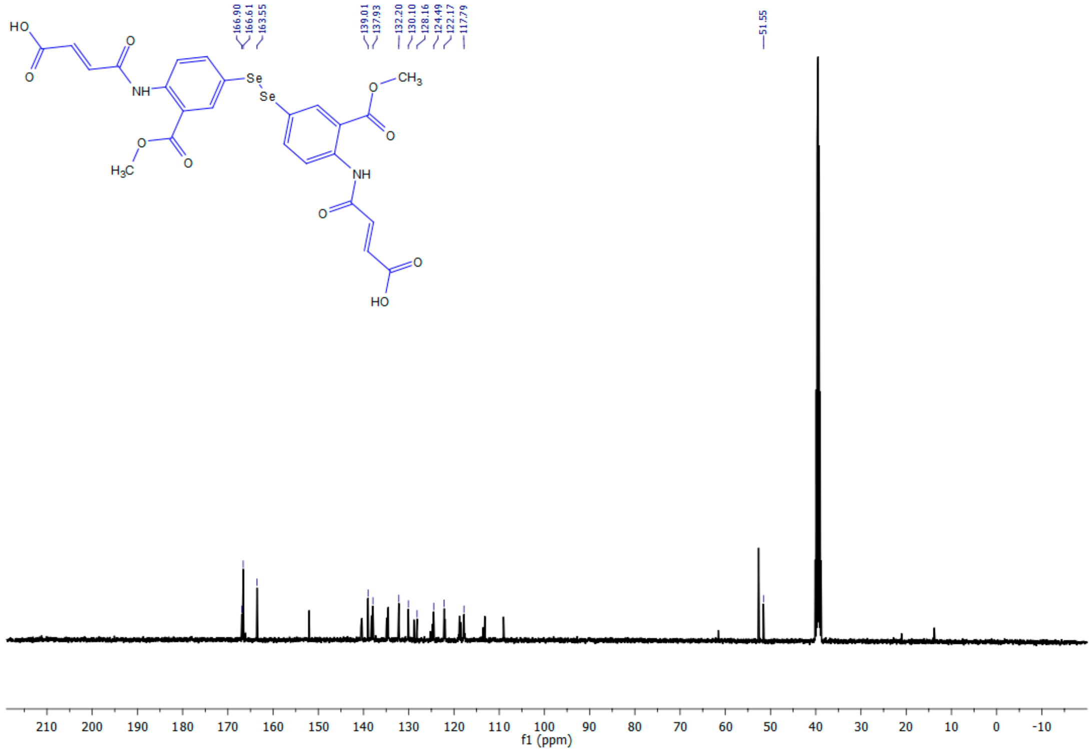

Figure S38. ^{13}CNMR chart of compound **13**

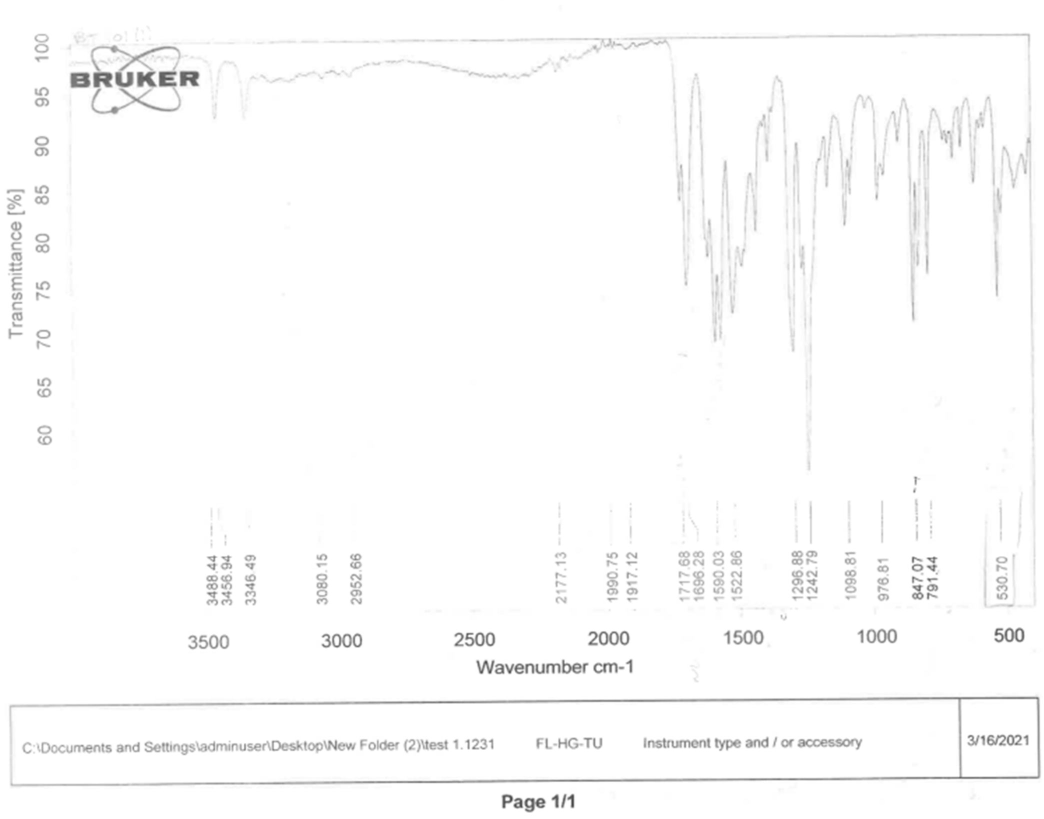

Figure S39. IR chart of compound **13**

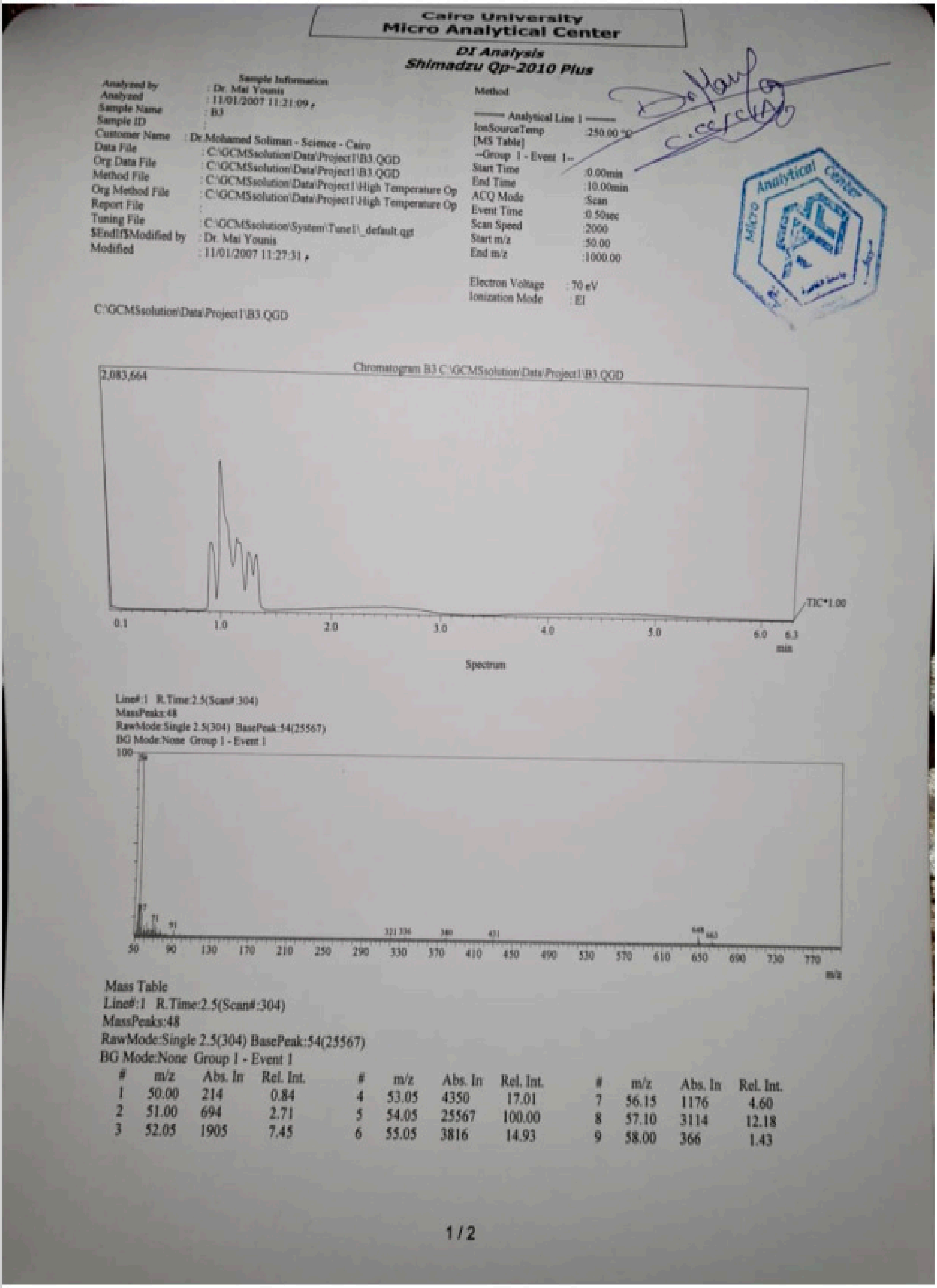

Cairo University
Micro Analytical Center

DI Analysis
Shimadzu Qp-2010 Plus

Sample Information

Analyzed by : Dr. Mai Younis
Analyzed : 11/01/2007 11:21:09 ,
Sample Name : B3
Sample ID :
Customer Name : Dr.Mohamed Soliman - Science - Cairo
Data File : C:\GCMSsolution\Data\Project1\B3.QGD
Org Data File : C:\GCMSsolution\Data\Project1\B3.QGD
Method File : C:\GCMSsolution\Data\Project1\High Temperature Op
Org Method File : C:\GCMSsolution\Data\Project1\High Temperature Op
Report File :
Tuning File : C:\GCMSsolution\System\Tune1_default.qgt
$EndIf$Modified by : Dr. Mai Younis
Modified : 11/01/2007 11:27:31 ,

Method

—— Analytical Line 1 ——
IonSourceTemp :250.00 °C
[MS Table]
--Group 1 - Event 1--
Start Time :0.00min
End Time :10.00min
ACQ Mode :Scan
Event Time :0.50sec
Scan Speed :2000
Start m/z :50.00
End m/z :1000.00

Electron Voltage : 70 eV
Ionization Mode : EI

C:\GCMSsolution\Data\Project1\B3.QGD

Chromatogram B3 C:\GCMSsolution\Data\Project1\B3.QGD

Spectrum

Line#:1 R.Time:2.5(Scan#:304)
MassPeaks:48
RawMode:Single 2.5(304) BasePeak:54(25567)
BG Mode:None Group 1 - Event 1

Mass Table
Line#:1 R.Time:2.5(Scan#:304)
MassPeaks:48
RawMode:Single 2.5(304) BasePeak:54(25567)
BG Mode:None Group 1 - Event 1

#	m/z	Abs. In	Rel. Int.	#	m/z	Abs. In	Rel. Int.	#	m/z	Abs. In	Rel. Int.
1	50.00	214	0.84	4	53.05	4350	17.01	7	56.15	1176	4.60
2	51.00	694	2.71	5	54.05	25567	100.00	8	57.10	3114	12.18
3	52.05	1905	7.45	6	55.05	3816	14.93	9	58.00	366	1.43

1/2

Figure S40. Mass chart of compound **13**

#	m/z	Abs. In	Rel. Int.
10	59.05	796	3.11
11	60.00	1414	5.53
12	60.95	613	2.40
13	62.05	489	1.91
14	63.05	1781	6.97
15	64.05	961	3.76
16	65.05	949	3.71
17	66.10	327	1.28
18	67.00	860	3.36
19	68.15	800	3.13
20	69.05	2439	9.54
21	70.05	817	3.20
22	71.05	1665	6.51

#	m/z	Abs. In	Rel. Int.
23	72.05	521	2.04
24	73.00	1263	4.94
25	74.00	391	1.53
26	75.00	334	1.31
27	76.00	244	0.95
28	76.95	919	3.59
29	78.00	487	1.90
30	79.00	273	1.07
31	81.00	207	0.81
32	82.00	210	0.82
33	83.00	279	1.09
34	85.00	239	0.93
35	91.10	807	3.16

#	m/z	Abs. In	Rel. Int.
36	92.10	225	0.88
37	98.10	209	0.82
38	321.10	258	1.01
39	336.10	350	1.37
40	378.10	212	0.83
41	380.10	263	1.03
42	429.10	201	0.79
43	431.10	204	0.80
44	647.70	1241	4.85
45	648.65	719	2.81
46	649.60	246	0.96
47	662.60	518	2.03
48	663.60	239	0.93

Mass chart of compound **13**

*Methyl 2-amino-5-(methylselanyl) benzoate (**14**).*

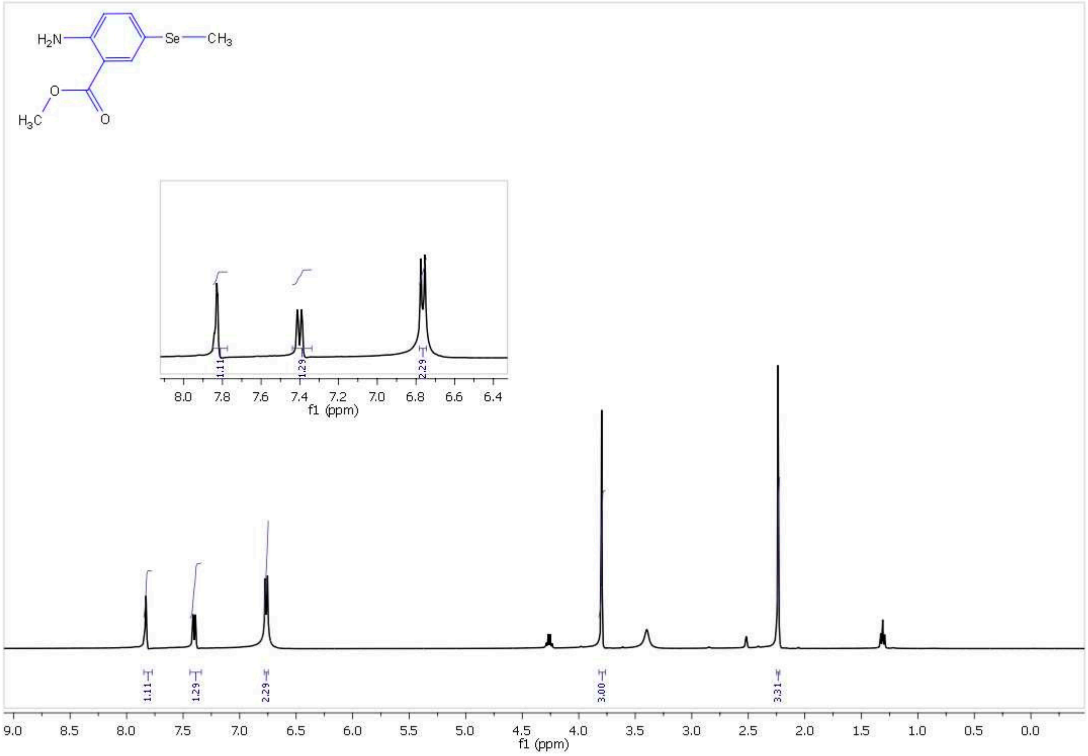

Figure S41. ^{1}HNMR chart of compound **14**

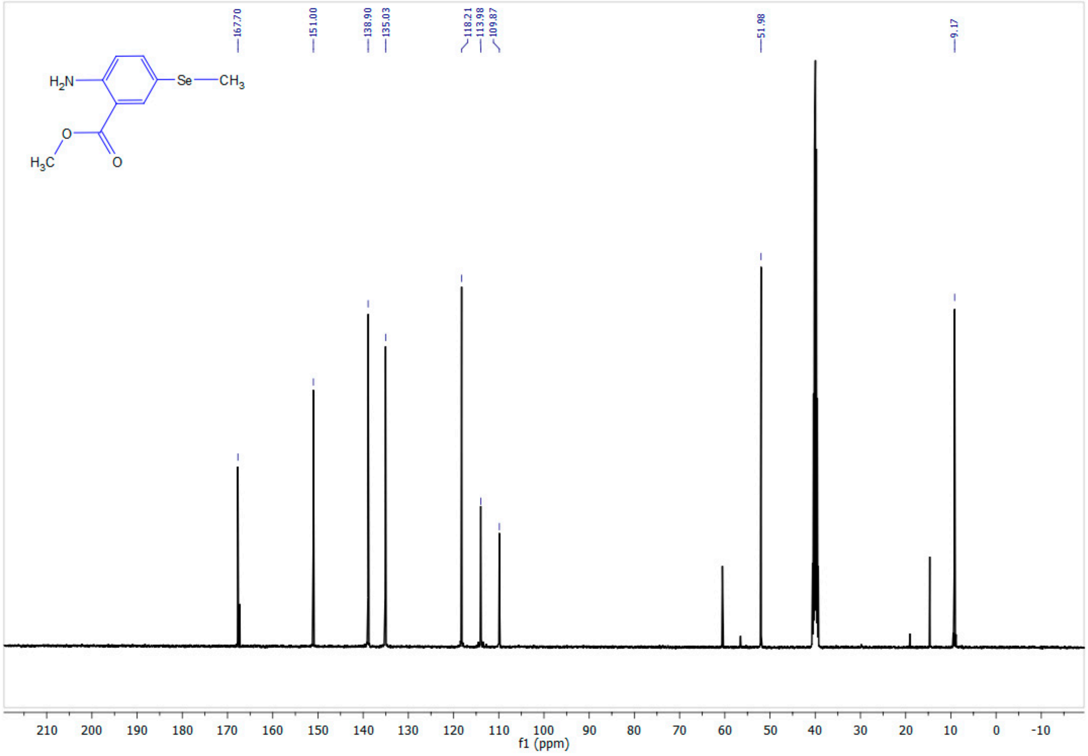

Figure S42. ^{13}CNMR chart of compound **14**

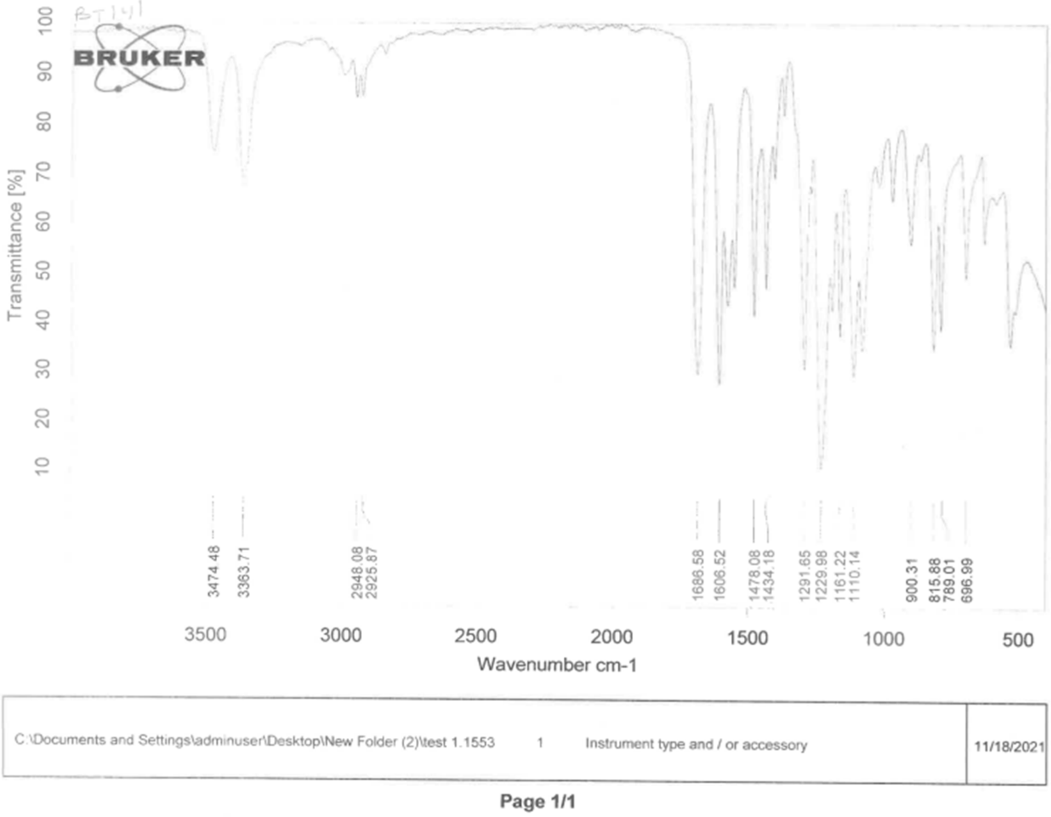

Figure S43. IR chart of compound **14**

Cairo University
Micro Analytical Center

DI Analysis
Shimadzu Qp-2010 Plus

Sample Information

Analyzed by	: Dr. Mai Younis
Analyzed	: 16/01/2007 03:02:11 ص
Sample Name	: B35
Sample ID	:
Customer Name	: Dr.Mohamed Soliman - Science - Cairo
Data File	: C:\GCMSsolution\Data\Project1\B35.QGD
Org Data File	: C:\GCMSsolution\Data\Project1\B35.QGD
Method File	: C:\GCMSsolution\Data\Project1\High Temperature Op
Org Method File	: C:\GCMSsolution\Data\Project1\High Temperature Op
Report File	:
Tuning File	: C:\GCMSsolution\System\Tune1_default.qgt
$End If$Modified by	: Dr. Mai Younis
Modified	: 16/01/2007 03:12:11 ص

Method

——— Analytical Line 1 ———

IonSourceTemp	:250.00 °C
[MS Table]	
--Group 1 - Event 1--	
Start Time	:0.00min
End Time	:10.00min
ACQ Mode	:Scan
Event Time	:0.50sec
Scan Speed	:1000
Start m/z	:50.00
End m/z	:510.00
Electron Voltage	: 70 eV
Ionization Mode	: EI

C:\GCMSsolution\Data\Project1\B35.QGD

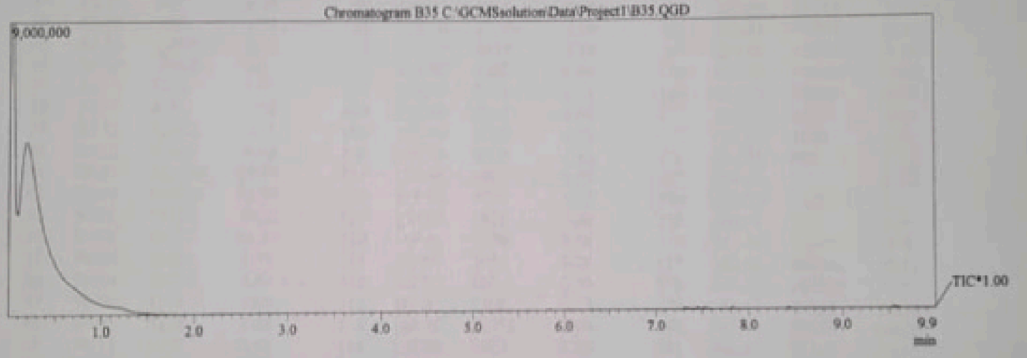

Spectrum

Line#:1 R.Time:0.2(Scan#:26)
MassPeaks:205
RawMode:Single 0.2(26) BasePeak:245(391154)
BG Mode:None Group 1 - Event 1

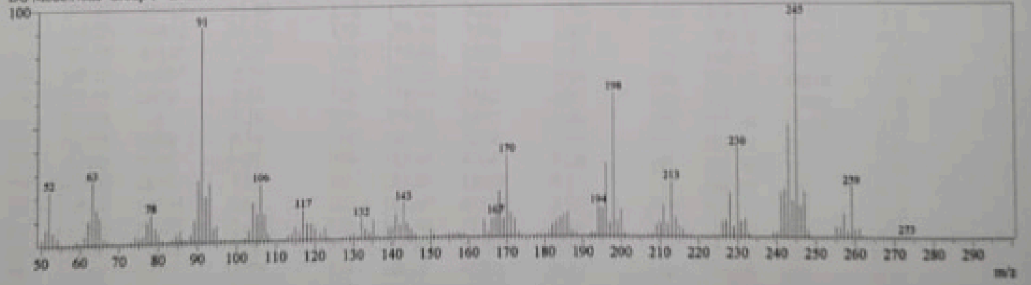

Mass Table
Line#:1 R.Time:0.2(Scan#:26)
MassPeaks:205
RawMode:Single 0.2(26) BasePeak:245(391154)
BG Mode:None Group 1 - Event 1

#	m/z	Abs. In	Rel. Int.	#	m/z	Abs. In	Rel. Int.	#	m/z	Abs. In	Rel. Int.
1	50.05	10411	2.66	4	53.00	21482	5.49	7	56.05	488	0.12
2	51.05	26260	6.71	5	54.05	11184	2.86	8	57.05	709	0.18
3	52.00	89298	22.83	6	55.00	2246	0.57	9	58.05	1164	0.30

1 / 2

Figure S44. Mass chart of compound **14**

16-Jan-07 03:18:00

#	m/z	Abs. In	Rel. Int.
10	59.00	6150	1.57
11	60.05	1575	0.40
12	61.05	14415	3.69
13	62.05	39733	10.16
14	63.05	103836	26.55
15	64.05	57318	14.65
16	65.05	43819	11.20
17	66.10	9838	2.52
18	67.05	4572	1.17
19	68.05	2933	0.75
20	69.10	1041	0.27
21	70.05	837	0.21
22	71.05	1646	0.42
23	72.05	1669	0.43
24	72.95	2952	0.75
25	74.05	12141	3.10
26	75.10	10198	2.61
27	76.10	12385	3.17
28	77.10	36498	9.33
29	78.05	47310	12.09
30	79.10	24441	6.25
31	80.00	15313	3.91
32	81.00	4794	1.23
33	82.55	1873	0.48
34	83.55	6981	1.78
35	84.55	13095	3.35
36	85.50	19868	5.08
37	86.10	5970	1.53
38	87.05	4792	1.23
39	88.10	12948	3.31
40	89.10	37161	9.50
41	90.15	101589	25.97
42	91.10	358905	91.76
43	92.05	75198	19.22
44	93.00	97377	24.89
45	94.00	22561	5.77
46	95.00	26670	6.82
47	96.00	4114	1.05
48	97.05	4225	1.08
49	98.35	2449	0.63
50	99.30	3386	0.87
51	100.25	1374	0.35
52	101.05	1757	0.45
53	102.10	5450	1.39
54	103.15	15671	4.01
55	104.15	62814	16.06
56	105.25	45718	11.69
57	106.20	94842	24.25
58	107.15	45188	11.55
59	108.15	10687	2.73
60	109.05	2089	0.53
61	110.05	710	0.18
62	111.05	1314	0.34
63	112.05	1199	0.31
64	113.00	7610	1.95
65	114.00	10354	2.65
66	115.00	21430	5.48
67	116.05	12818	3.28
68	117.05	50031	12.79
69	118.05	27671	7.07
70	119.10	26692	6.82
71	120.10	18391	4.70
72	121.55	12099	3.09
73	122.60	19750	5.05
74	123.55	4338	1.11
75	125.05	1061	0.27
76	126.05	2179	0.56
77	127.05	3204	0.82
78	128.00	4023	1.03
79	129.05	5617	1.44
80	130.00	7762	1.98
81	131.15	4478	1.14
82	132.10	33902	8.67
83	133.10	15628	4.00
84	134.15	8746	2.24
85	135.10	31116	7.95
86	136.15	5476	1.40
87	137.00	2783	0.71
88	138.05	3352	0.86
89	139.00	15473	3.96
90	140.00	17660	4.51
91	141.00	39022	9.98
92	142.05	21311	5.45
93	143.00	58711	15.01
94	144.00	25175	6.44
95	145.00	13801	3.53
96	146.00	5042	1.29
97	147.00	1039	0.27
98	148.10	804	0.21
99	149.15	1515	0.39
100	150.10	13154	3.36
101	151.10	4439	1.13
102	151.95	1797	0.46
103	153.00	3161	0.81
104	154.00	3514	0.90
105	155.00	5122	1.31
106	156.00	4514	1.15
107	157.05	6492	1.66
108	158.00	3975	1.02
109	159.00	5625	1.44
110	160.00	1470	0.38
111	161.00	1084	0.28
112	162.00	380	0.10
113	163.15	1307	0.33
114	164.10	28951	7.40
115	165.05	9689	2.48
116	166.00	30183	7.72
117	167.05	33926	8.67
118	168.00	75583	19.32
119	169.05	30276	7.74
120	170.00	137220	35.08
121	171.00	40180	10.27
122	172.00	30348	7.76
123	173.00	7290	1.86
124	174.00	1239	0.32
125	177.20	238	0.06
126	178.15	3162	0.81
127	179.05	1817	0.46
128	180.00	3514	0.90
129	181.05	9794	2.50
130	182.00	19970	5.11
131	183.00	25158	6.43
132	184.00	31201	7.98
133	185.00	38050	9.73
134	186.00	40924	10.46
135	187.00	10028	2.56
136	188.00	6714	1.72
137	189.00	868	0.22
138	191.05	296	0.08
139	192.00	4826	1.23
140	193.05	2875	0.74
141	194.00	49812	12.73
142	195.00	47017	12.02
143	196.00	122146	31.23
144	197.05	22307	5.70
145	198.00	241660	61.78
146	198.95	25629	6.55
147	200.00	43719	11.18
148	200.95	4204	1.07
149	201.95	533	0.14
150	207.00	2228	0.57
151	208.05	2526	0.65
152	209.05	20084	5.13
153	210.05	23473	6.00
154	211.00	50268	12.85
155	212.05	22070	5.64
156	213.00	91327	23.35
157	214.00	31220	7.98
158	215.00	18164	4.64
159	216.00	12508	3.20
160	216.95	1191	0.30
161	218.00	1618	0.41
162	224.00	2599	0.66
163	225.05	1510	0.39
164	226.05	26426	6.76
165	227.05	28172	7.20
166	228.05	72774	18.60
167	229.05	16399	4.19
168	230.05	148867	38.06
169	231.00	25292	6.47
170	232.05	29923	7.65
171	233.00	4730	1.21
172	233.95	605	0.15
173	238.15	734	0.19
174	239.10	6298	1.61
175	240.15	7887	2.02
176	241.10	72847	18.62
177	242.10	80234	20.51
178	243.10	187193	47.86
179	244.15	60066	15.36
180	245.10	391154	100.00
181	246.05	51306	13.12
182	247.05	74480	19.04
183	248.05	8926	2.28
184	249.05	953	0.24
185	253.10	1460	0.37
186	254.15	1011	0.26
187	255.10	15329	3.92
188	256.15	16105	4.12
189	257.10	40538	10.36
190	258.15	9549	2.44
191	259.10	84319	21.56
192	260.05	12047	3.08
193	261.10	15478	3.96
194	262.05	2350	0.60
195	263.05	335	0.09
196	264.10	220	0.06
197	269.15	730	0.19
198	270.15	629	0.16
199	271.15	1493	0.38
200	272.25	533	0.14
201	273.15	2823	0.72
202	274.20	558	0.14
203	275.20	654	0.17
204	276.20	290	0.07
205	277.10	369	0.09

Mass chart of compound **14**

*Methyl 2-amino-5-(benzylselanyl) benzoate (**15**).*

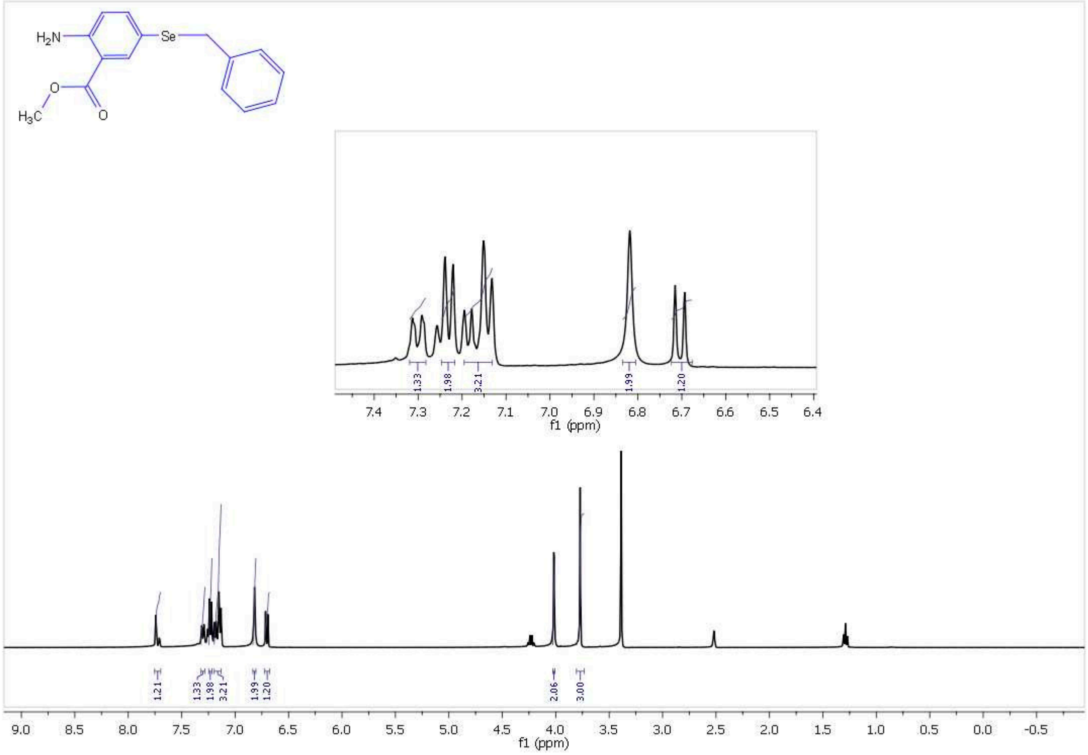

Figure S45. ^{1}HNMR chart of compound **15**

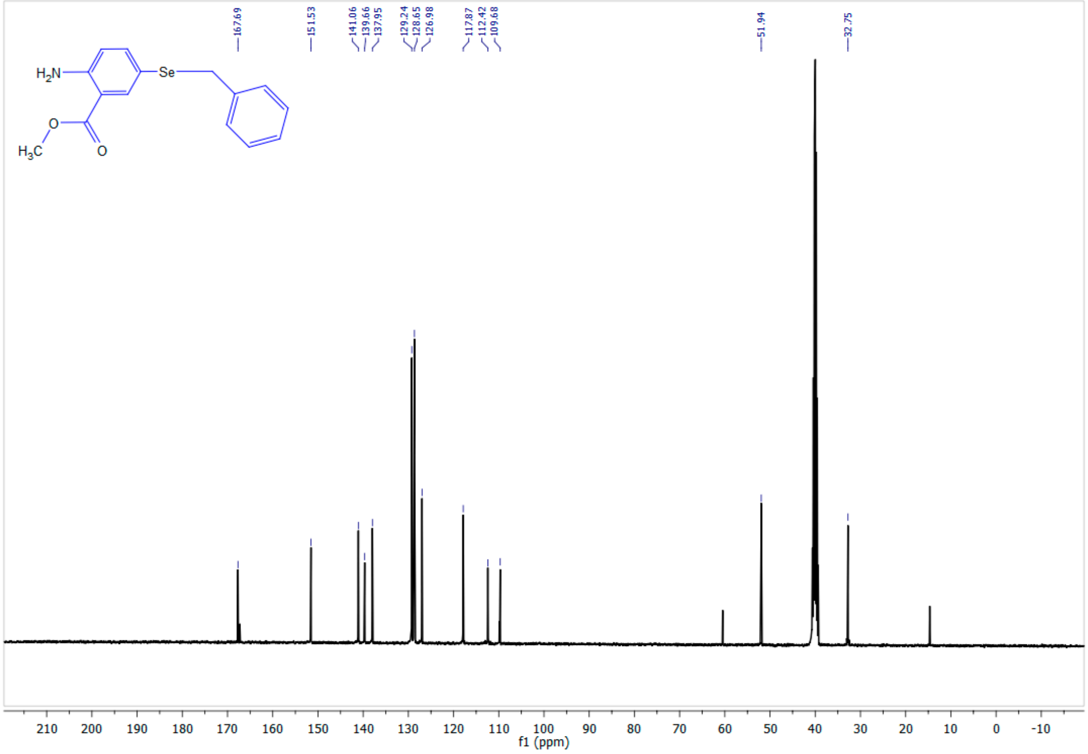

Figure S46. ^{13}CNMR chart of compound 15

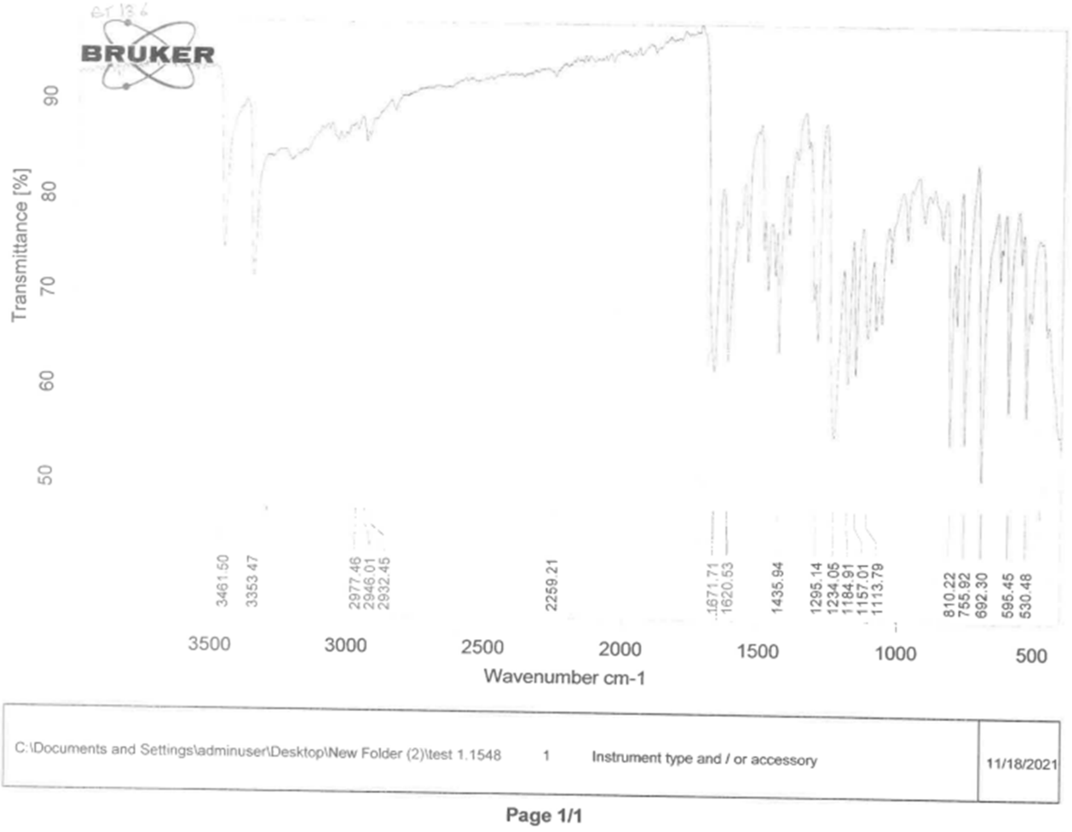

Figure S47. IR chart of compound **15**

06-Jan-07 19:54:17

Cairo University
Micro Analytical Center

DI Analysis
Shimadzu Qp-2010 Plus

Sample Information

Analyzed by : Dr. Mai Younis
Analyzed : 06/01/2007 07:49:46 م
Sample Name : B22
Sample ID :
Customer Name : Dr.Mohamed Soliman - Science - Cairo
Data File : C:\GCMSsolution\Data\Project1\B22.QGD
Org Data File : C:\GCMSsolution\Data\Project1\B22.QGD
Method File : C:\GCMSsolution\Data\Project1\High Temperature Op
Org Method File : C:\GCMSsolution\Data\Project1\High Temperature Op
Report File :
Tuning File : C:\GCMSsolution\System\Tune1_default.qgt
$EndIf$Modified by : Dr. Mai Younis
Modified : 06/01/2007 07:51:49 م

Method

------ Analytical Line 1 ------
IonSourceTemp :250.00 °C
[MS Table]
--Group 1 - Event 1--
Start Time :0.00min
End Time :10.00min
ACQ Mode :Scan
Event Time :0.50sec
Scan Speed :1000
Start m/z :50.00
End m/z :510.00

Electron Voltage : 70 eV
Ionization Mode : EI

C:\GCMSsolution\Data\Project1\B22.QGD

Chromatogram B22 C:\GCMSsolution\Data\Project1\B22.QGD

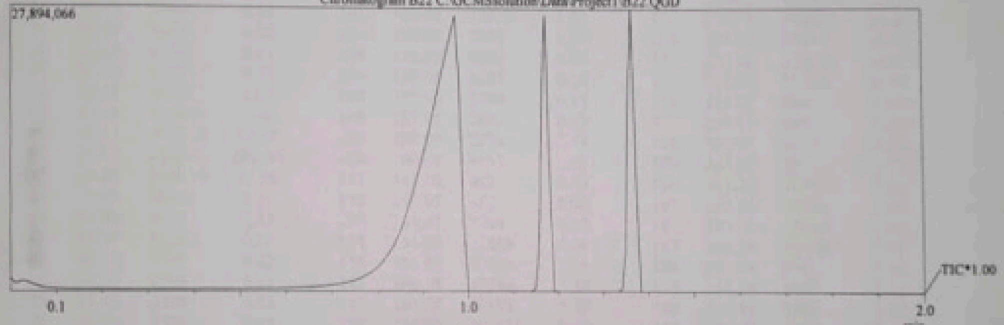

Spectrum

Line#:1 R.Time:1.2(Scan#:143)
MassPeaks:244(Peak Elimination m/z: 423.20, 425.30)
RawMode:Single 1.2(143) BasePeak:91(2025185)
BG Mode:None Group 1 - Event 1

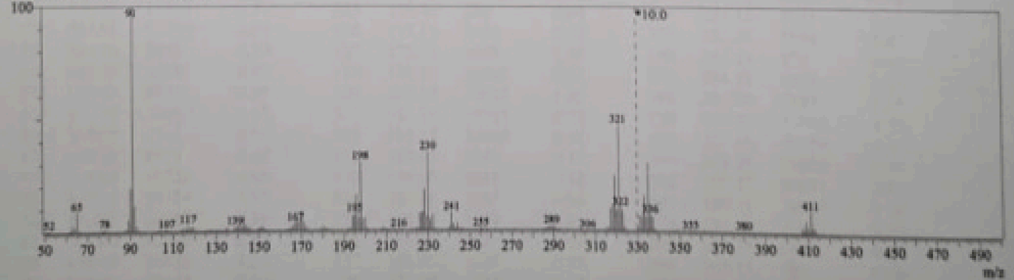

Mass Table
Line#:1 R.Time:1.2(Scan#:143)
MassPeaks:244(Peak Elimination m/z: 423.20, 425.30)
RawMode:Single 1.2(143) BasePeak:91(2025185)
BG Mode:None Group 1 - Event 1

#	m/z	Abs. In	Rel. Int.	#	m/z	Abs. In	Rel. Int.	#	m/z	Abs. In	Rel. Int.
1	50.05	5073	0.25	4	52.95	3146	0.16	7	57.00	1097	0.05
2	51.00	11465	0.57	5	54.00	1751	0.09	8	58.05	483	0.02
3	52.00	13521	0.67	6	55.00	583	0.03	9	59.00	2993	0.15

1 / 3

Figure S48. Mass chart of compound **15**

#	m/z	Abs. In	Rel. Int.
10	60.05	391	0.02
11	61.05	3772	0.19
12	62.05	17107	0.84
13	63.00	56926	2.81
14	64.05	36812	1.82
15	65.00	176463	8.71
16	65.95	12878	0.64
17	67.00	1580	0.08
18	68.05	483	0.02
19	69.10	985	0.05
20	70.10	1497	0.07
21	71.10	1663	0.08
22	72.05	379	0.02
23	73.05	2080	0.10
24	74.05	4921	0.24
25	75.05	5212	0.26
26	76.05	6114	0.30
27	77.05	10886	0.54
28	78.05	20811	1.03
29	79.05	8101	0.40
30	80.00	5090	0.25
31	81.05	1605	0.08
32	82.15	1045	0.05
33	83.10	2590	0.13
34	84.15	2599	0.13
35	85.15	4470	0.22
36	86.05	4174	0.21
37	87.15	6312	0.31
38	88.15	20787	1.03
39	89.15	110480	5.46
40	90.15	391518	19.33
41	91.05	202518	100.00
42	92.05	228419	11.28
43	93.05	46032	2.27
44	94.00	4180	0.21
45	95.00	8522	0.42
46	96.15	1388	0.07
47	97.10	2596	0.13
48	98.10	2490	0.12
49	99.10	2721	0.13
50	100.15	911	0.04
51	101.10	2750	0.14
52	102.10	4402	0.22
53	103.10	7242	0.36
54	104.05	11796	0.58
55	105.05	7012	0.35
56	106.10	11636	0.57
57	107.05	13258	0.65
58	108.00	3873	0.19
59	109.15	1378	0.07
60	110.05	979	0.05
61	111.05	2440	0.12
62	112.05	2793	0.14
63	113.05	9979	0.49
64	114.05	12822	0.63
65	115.00	27724	1.37
66	116.05	18052	0.89
67	117.00	53988	2.67
68	118.05	26835	1.33
69	119.05	43451	2.15
70	120.05	15785	0.78
71	121.10	4878	0.24
72	122.05	2063	0.10
73	123.10	1527	0.08
74	124.05	682	0.03
75	125.05	4002	0.20
76	126.05	5459	0.27
77	127.05	10480	0.52
78	128.05	10844	0.54

#	m/z	Abs. In	Rel. Int.
79	129.05	9313	0.46
80	130.05	11086	0.55
81	131.10	5054	0.25
82	132.05	10987	0.54
83	133.10	3684	0.18
84	134.15	9264	0.46
85	135.05	30121	1.49
86	136.05	4109	0.20
87	137.05	5343	0.26
88	138.05	7879	0.39
89	139.00	35607	1.76
90	140.05	41787	2.06
91	141.00	77244	3.81
92	142.05	47101	2.33
93	142.95	126529	6.25
94	143.95	48735	2.41
95	144.95	28945	1.43
96	146.05	23129	1.14
97	146.95	4067	0.20
98	148.15	1843	0.09
99	149.15	8597	0.42
100	150.15	20578	1.02
101	151.05	33271	1.64
102	152.05	32505	1.61
103	153.05	20872	1.03
104	154.05	7855	0.39
105	155.00	4855	0.24
106	155.95	3585	0.18
107	157.00	3398	0.17
108	157.95	2693	0.13
109	159.05	2274	0.11
110	160.05	4165	0.21
111	161.00	602	0.03
112	162.05	482	0.02
113	163.05	3714	0.18
114	164.05	12889	0.64
115	165.05	22436	1.11
116	166.05	47284	2.33
117	167.05	66556	3.29
118	168.00	102111	5.04
119	169.05	77098	3.81
120	169.95	174770	8.63
121	170.95	74461	3.68
122	172.00	39018	1.93
123	172.95	11808	0.58
124	173.95	1305	0.06
125	176.15	889	0.04
126	177.15	2401	0.12
127	178.15	5969	0.29
128	179.15	10187	0.50
129	180.15	36701	1.81
130	181.10	18865	0.93
131	182.15	14965	0.74
132	183.05	3441	0.17
133	184.00	2350	0.12
134	185.10	1281	0.06
135	186.05	1167	0.06
136	190.05	602	0.03
137	191.05	1736	0.09
138	192.05	12467	0.62
139	193.05	17901	0.88
140	194.05	131489	6.49
141	195.05	146464	7.23
142	196.00	323593	15.98
143	197.05	113333	5.60
144	198.00	620129	30.62
145	198.95	99120	4.89
146	200.00	113441	5.60
147	200.95	16159	0.80

#	m/z	Abs. In	Rel. Int.
148	201.95	1028	0.05
149	204.25	5464	0.27
150	205.15	701	0.03
151	206.20	1020	0.05
152	207.25	2877	0.14
153	208.20	14284	0.71
154	209.15	17645	0.87
155	210.05	5896	0.29
156	211.15	1898	0.09
157	212.05	5471	0.27
158	213.10	2154	0.11
159	214.05	4336	0.21
160	215.15	994	0.05
161	216.10	6855	0.34
162	217.05	395	0.02
163	218.10	921	0.05
164	221.20	846	0.04
165	223.15	1595	0.08
166	224.10	14468	0.71
167	225.15	19403	0.96
168	226.10	153471	7.58
169	227.15	166163	8.20
170	228.10	368934	18.22
171	229.15	120571	5.95
172	230.10	706377	34.88
173	231.05	94541	4.67
174	232.10	132870	6.56
175	233.05	15116	0.75
176	234.05	960	0.05
177	238.15	969	0.05
178	239.25	4131	0.20
179	240.25	35113	1.73
180	241.25	155831	7.69
181	242.10	54386	2.69
182	243.15	12588	0.62
183	244.10	56178	2.77
184	245.05	8044	0.40
185	246.10	9770	0.48
186	247.10	1503	0.07
187	254.35	1823	0.09
188	255.25	10721	0.53
189	256.15	3888	0.19
190	257.15	1891	0.09
191	258.15	4289	0.21
192	259.15	3074	0.15
193	260.15	2910	0.14
194	261.15	2231	0.11
195	262.20	1594	0.08
196	283.25	376	0.02
197	284.25	1803	0.09
198	285.20	7185	0.35
199	286.20	14742	0.73
200	287.15	23239	1.15
201	288.20	24534	1.21
202	289.15	36611	1.81
203	290.15	36028	1.78
204	291.10	11612	0.57
205	292.15	6462	0.32
206	293.15	552	0.03
207	306.20	302	0.01
208	308.10	636	0.03
209	310.05	707	0.03
210	314.25	1728	0.09
211	315.20	18481	0.91
212	316.25	23945	1.18
213	317.20	196281	9.69
214	318.25	227987	11.26
215	319.20	494574	24.42
216	320.25	188395	9.30

2 / 3

Mass chart of compound **15**

06-Jan-07 19:54:17

#	m/z	Abs. In	Rel. Int.
217	321.20	949119	46.87
218	322.15	206457	10.19
219	323.15	182324	9.00
220	324.05	37472	1.85
221	325.05	4024	0.20
222	329.25	824	0.04
223	330.25	1720	0.08
224	331.20	13207	0.65
225	332.25	14297	0.71
226	333.20	31734	1.57

#	m/z	Abs. In	Rel. Int.
227	334.25	12617	0.62
228	335.20	61590	3.04
229	336.15	14251	0.70
230	337.20	12284	0.61
231	338.15	2137	0.11
232	347.20	470	0.02
233	349.20	1524	0.08
234	355.20	474	0.02
235	378.20	226	0.01
236	380.20	604	0.03

#	m/z	Abs. In	Rel. Int.
237	407.30	3812	0.19
238	408.35	4545	0.22
239	409.25	10425	0.51
240	410.35	4385	0.22
241	411.25	19850	0.98
242	412.25	5834	0.29
243	413.25	4131	0.20
244	414.15	786	0.04

3 / 3

Mass chart of compound **15**

Methyl 2-amino-5-((2-oxo-2-(phenylamino) ethyl) selanyl) benzoate (16).

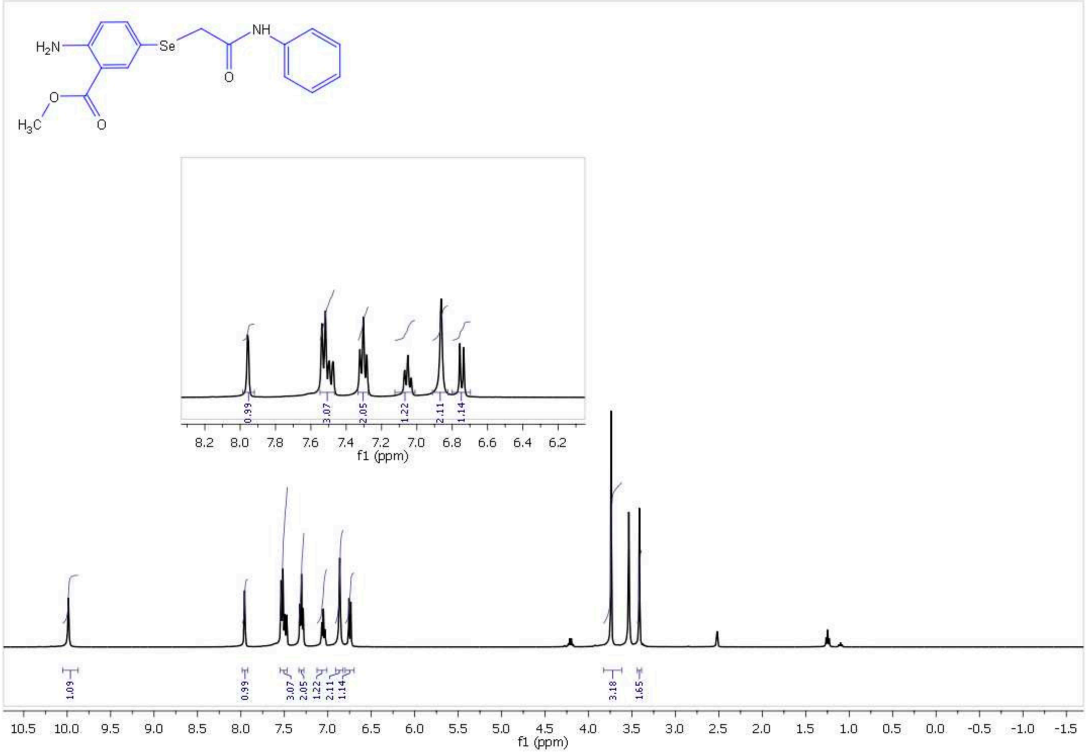

Figure S49. ^{1}HNMR chart of compound **16**

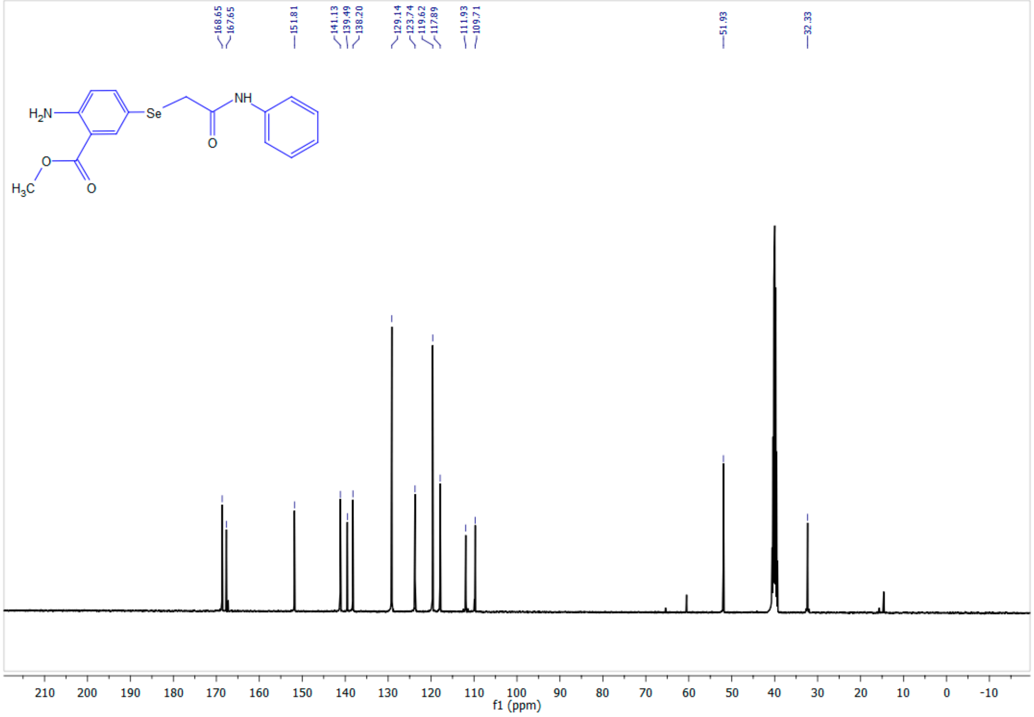

Figure S50. ^{13}CNMR chart of compound **16**

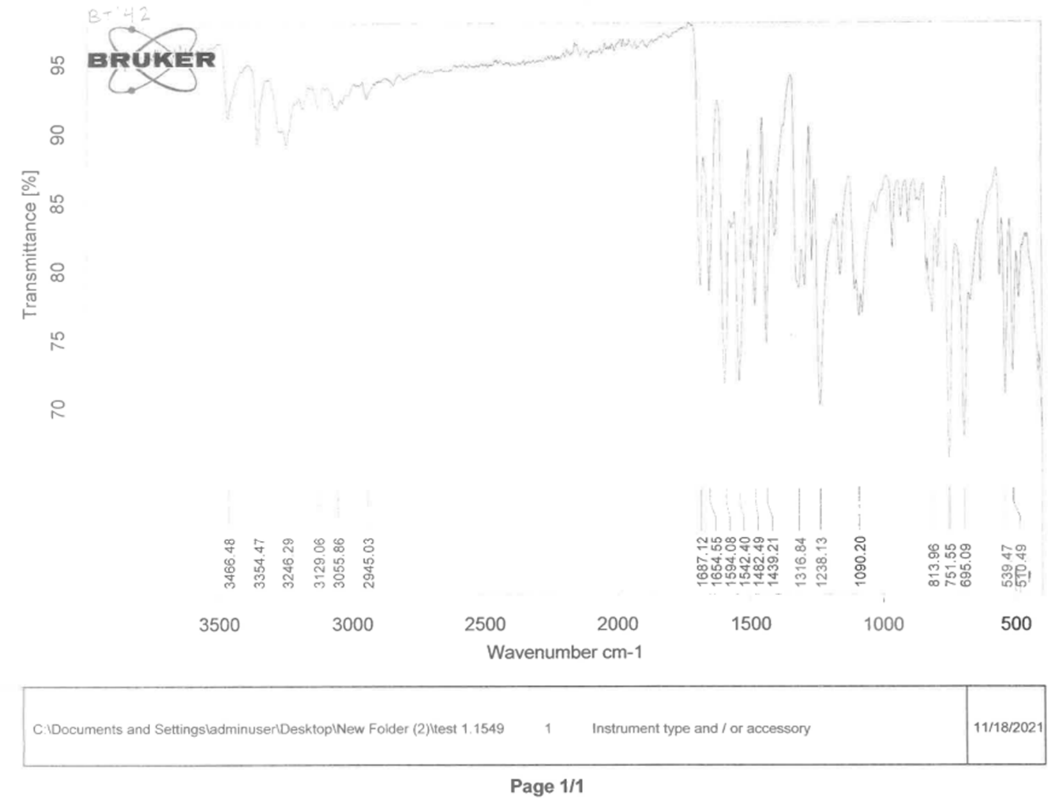

Figure S51. IR chart of compound **16**

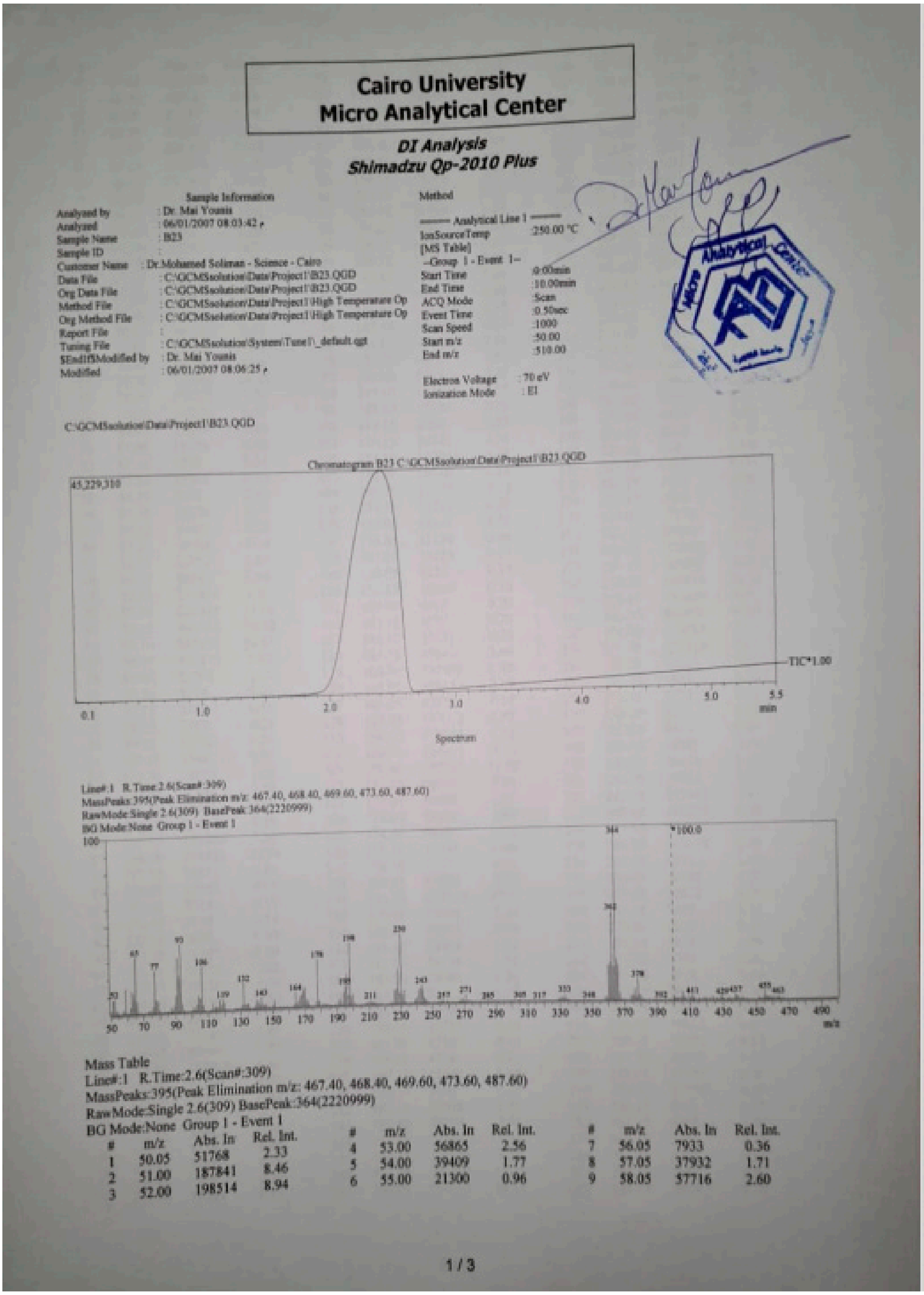

Cairo University
Micro Analytical Center

DI Analysis
Shimadzu Qp-2010 Plus

Sample Information

Analyzed by : Dr. Mai Younis
Analyzed : 06/01/2007 08:03:42 م
Sample Name : B23
Sample ID :
Customer Name : Dr.Mohamed Soliman - Science - Cairo
Data File : C:\GCMSsolution\Data\Project1\B23.QGD
Org Data File : C:\GCMSsolution\Data\Project1\B23.QGD
Method File : C:\GCMSsolution\Data\Project1\High Temperature Op
Org Method File : C:\GCMSsolution\Data\Project1\High Temperature Op
Report File :
Tuning File : C:\GCMSsolution\System\Tune1_default.qgt
$EndIf$Modified by : Dr. Mai Younis
Modified : 06/01/2007 08:06:25 م

Method

—— Analytical Line 1 ——
IonSourceTemp :250.00 °C
[MS Table]
--Group 1 - Event 1--
Start Time :0.00min
End Time :10.00min
ACQ Mode :Scan
Event Time :0.50sec
Scan Speed :1000
Start m/z :50.00
End m/z :510.00

Electron Voltage : 70 eV
Ionization Mode : EI

C:\GCMSsolution\Data\Project1\B23.QGD

Mass Table
Line#:1 R.Time:2.6(Scan#:309)
MassPeaks:395(Peak Elimination m/z: 467.40, 468.40, 469.60, 473.60, 487.60)
RawMode:Single 2.6(309) BasePeak:364(2220999)
BG Mode:None Group 1 - Event 1

#	m/z	Abs. In	Rel. Int.	#	m/z	Abs. In	Rel. Int.	#	m/z	Abs. In	Rel. Int.
1	50.05	51768	2.33	4	53.00	56865	2.56	7	56.05	7933	0.36
2	51.00	187841	8.46	5	54.00	39409	1.77	8	57.05	37932	1.71
3	52.00	198514	8.94	6	55.00	21300	0.96	9	58.05	57716	2.60

1 / 3

Figure S52. Mass chart of compound **16**

#	m/z	Abs. In	Rel. Int.
10	59.00	323380	14.56
11	60.00	19518	0.88
12	61.05	25294	1.14
13	62.05	85663	3.86
14	63.05	284974	12.83
15	64.05	250676	11.29
16	65.00	718538	32.35
17	66.00	152620	6.87
18	66.95	28433	1.28
19	68.05	8877	0.40
20	69.05	15359	0.69
21	70.05	8547	0.38
22	71.05	14796	0.67
23	72.05	7256	0.33
24	73.05	29968	1.35
25	74.05	26922	1.21
26	75.05	38018	1.71
27	76.05	89648	4.04
28	77.05	535908	24.13
29	78.05	171575	7.73
30	79.05	144276	6.50
31	80.05	37639	1.69
32	81.05	11176	0.50
33	82.15	5419	0.24
34	83.10	15562	0.70
35	84.15	7935	0.36
36	85.10	16374	0.74
37	86.15	16002	0.72
38	87.10	69636	3.14
39	88.15	32733	1.47
40	89.15	88448	3.98
41	90.15	210874	9.49
42	91.10	688561	31.00
43	92.15	463765	20.88
44	93.10	862055	38.81
45	94.05	112159	5.05
46	95.00	27708	1.25
47	96.10	5274	0.24
48	97.10	9146	0.41
49	98.15	5237	0.24
50	99.10	12962	0.58
51	100.15	13487	0.61
52	101.10	60012	2.70
53	102.15	30642	1.38
54	103.15	88585	3.99
55	104.10	204116	9.19
56	105.15	159966	7.20
57	106.10	568420	25.59
58	107.10	76871	3.46
59	108.05	12580	0.57
60	109.10	3719	0.17
61	110.15	3039	0.14
62	111.15	9856	0.44
63	112.15	15347	0.69
64	113.10	93678	4.22
65	114.15	34219	1.54
66	115.10	49390	2.22
67	116.15	38664	1.74
68	117.10	130407	5.87
69	118.15	63644	2.87
70	119.10	142945	6.44
71	120.10	98100	4.42
72	121.05	14291	0.64
73	122.10	5969	0.27
74	123.05	3081	0.14
75	124.15	1538	0.07
76	125.15	3955	0.18
77	126.15	7068	0.32
78	127.15	19469	0.88

#	m/z	Abs. In	Rel. Int.
79	128.15	17920	0.81
80	129.15	16582	0.75
81	130.15	23991	1.08
82	131.15	62358	2.81
83	132.10	333977	15.04
84	133.15	66067	2.97
85	134.15	78340	3.53
86	135.15	69517	3.13
87	136.15	11769	0.53
88	137.10	7681	0.35
89	138.15	13090	0.59
90	139.15	52322	2.36
91	140.10	117575	5.29
92	141.05	90195	4.06
93	142.05	52676	2.37
94	143.00	141599	6.38
95	144.00	54009	2.43
96	145.05	43425	1.96
97	146.05	54032	2.43
98	147.10	12569	0.57
99	148.10	17756	0.80
100	149.15	8208	0.37
101	150.15	38743	1.74
102	151.10	102964	4.64
103	152.05	16710	0.75
104	153.10	11177	0.50
105	154.10	14711	0.66
106	155.05	15641	0.70
107	156.05	11100	0.50
108	157.05	15214	0.69
109	158.05	8233	0.37
110	159.10	16986	0.76
111	160.05	4503	0.20
112	161.15	4537	0.20
113	162.15	5173	0.23
114	163.15	35647	1.60
115	164.10	195450	8.80
116	165.05	50811	2.29
117	166.05	92270	4.15
118	167.05	149172	6.72
119	168.05	172761	7.78
120	169.05	226041	10.18
121	170.00	257615	11.60
122	171.00	96798	4.36
123	172.00	69998	3.15
124	173.00	21033	0.95
125	174.10	7897	0.36
126	175.15	17735	0.80
127	176.15	7737	0.35
128	177.15	62536	2.82
129	178.10	605762	27.27
130	179.05	72024	3.24
131	180.05	20815	0.94
132	181.10	26969	1.21
133	182.05	36544	1.65
134	183.10	28086	1.26
135	184.05	26423	1.19
136	185.00	19938	0.90
137	186.00	10751	0.48
138	187.05	6516	0.29
139	188.25	4750	0.21
140	189.15	25047	1.13
141	190.15	9994	0.45
142	191.15	57570	2.59
143	192.10	82754	3.73
144	193.05	33585	1.51
145	194.05	172249	7.76
146	195.05	249002	11.21
147	196.00	413670	18.63

#	m/z	Abs. In	Rel. Int.
148	197.05	127657	5.75
149	198.00	802486	36.13
150	198.95	111117	5.00
151	200.00	147049	6.62
152	201.00	27327	1.23
153	202.05	6591	0.30
154	203.15	3651	0.16
155	204.15	2634	0.12
156	205.10	8610	0.39
157	206.05	3512	0.16
158	207.05	11680	0.53
159	208.05	15928	0.72
160	209.05	33769	1.52
161	210.05	30291	1.36
162	211.05	55042	2.48
163	212.05	30408	1.37
164	213.05	31320	1.41
165	214.05	18869	0.85
166	215.05	9367	0.42
167	216.05	19840	0.89
168	217.05	4333	0.20
169	218.05	4479	0.20
170	219.10	2612	0.12
171	220.10	778	0.04
172	221.15	1397	0.06
173	222.15	1142	0.05
174	223.15	4881	0.22
175	224.05	17976	0.81
176	225.15	20916	0.94
177	226.10	174902	7.87
178	227.15	189200	8.52
179	228.05	447454	20.15
180	229.15	133404	6.01
181	230.05	899020	40.48
182	231.05	114924	5.17
183	232.05	173318	7.80
184	233.05	21637	0.97
185	234.05	3484	0.16
186	235.15	3179	0.14
187	236.15	3807	0.17
188	237.05	8985	0.40
189	238.15	10887	0.49
190	239.10	48174	2.17
191	240.10	75681	3.41
192	241.10	157647	7.10
193	242.15	186228	8.38
194	243.10	230793	10.39
195	244.05	179124	8.07
196	245.05	65413	2.95
197	246.05	36727	1.65
198	247.20	22822	1.03
199	248.15	7827	0.35
200	249.15	2124	0.10
201	250.25	1855	0.08
202	251.20	7351	0.33
203	252.15	3123	0.14
204	253.15	6626	0.30
205	254.15	7024	0.32
206	255.10	19212	0.87
207	256.15	19273	0.87
208	257.10	29007	1.31
209	258.15	10175	0.46
210	259.20	21718	0.98
211	260.15	6061	0.27
212	261.15	4356	0.20
213	262.15	3391	0.15
214	263.15	3353	0.15
215	264.10	1687	0.08
216	265.15	3515	0.16

Mass chart of compound **16**

#	m/z	Abs. In	Rel. Int.
217	266.15	2637	0.12
218	267.10	18146	0.82
219	268.15	20158	0.91
220	269.10	46786	2.11
221	270.15	15694	0.71
222	271.10	96137	4.33
223	272.05	16070	0.72
224	273.10	21305	0.96
225	274.05	3890	0.18
226	275.15	1899	0.09
227	276.25	1345	0.06
228	277.20	2796	0.13
229	278.15	1435	0.06
230	279.15	898	0.04
231	280.15	727	0.03
232	281.15	2478	0.11
233	282.15	2409	0.11
234	283.10	5790	0.26
235	284.15	3531	0.16
236	285.10	12303	0.55
237	286.15	5148	0.23
238	287.10	5833	0.26
239	288.25	2633	0.12
240	289.15	7201	0.32
241	290.15	2596	0.12
242	291.20	2186	0.10
243	292.15	1131	0.05
244	293.25	1594	0.07
245	294.25	1118	0.05
246	295.20	5345	0.24
247	296.15	1761	0.08
248	297.25	811	0.04
249	298.35	617	0.03
250	299.30	823	0.04
251	300.25	1273	0.06
252	301.25	1429	0.06
253	302.25	2061	0.09
254	303.30	3346	0.15
255	304.35	3895	0.18
256	305.30	14001	0.63
257	306.20	5601	0.25
258	307.15	2298	0.10
259	308.15	902	0.04
260	309.30	892	0.04
261	310.25	583	0.03
262	311.35	721	0.03
263	312.35	712	0.03
264	313.35	1160	0.05
265	314.35	803	0.04
266	315.35	918	0.04
267	316.35	904	0.04
268	317.30	4386	0.20
269	318.25	2391	0.11
270	319.25	2801	0.13
271	320.25	1770	0.08
272	321.20	4260	0.19
273	322.25	2058	0.09
274	323.25	2501	0.11
275	324.25	848	0.04
276	325.15	508	0.02
277	326.15	836	0.04
278	327.15	2834	0.13
279	328.15	6050	0.27
280	329.15	23275	1.05
281	330.15	29151	1.31
282	331.15	50733	2.28
283	332.15	33893	1.53
284	333.15	88344	3.98
285	334.05	24078	1.08
286	335.15	19856	0.89
287	336.15	4538	0.20
288	337.15	1006	0.05
289	338.25	662	0.03
290	339.30	1508	0.07
291	340.25	578	0.03
292	341.25	684	0.03
293	342.25	584	0.03
294	343.25	805	0.04
295	344.20	1958	0.09
296	345.25	2706	0.12
297	346.20	3890	0.18
298	347.25	4640	0.21
299	348.20	7434	0.33
300	349.15	2578	0.12
301	350.15	2611	0.12
302	351.20	977	0.04
303	352.15	764	0.03
304	353.25	847	0.04
305	354.15	553	0.02
306	355.25	657	0.03
307	356.25	704	0.03
308	357.25	3443	0.16
309	358.20	42783	1.93
310	359.25	41697	1.88
311	360.20	448631	20.20
312	361.25	490508	22.09
313	362.20	113024	50.89
314	363.25	433043	19.50
315	364.20	222099	100.00
316	365.15	516139	23.24
317	366.15	453240	20.41
318	367.15	94607	4.26
319	368.15	13453	0.61
320	369.15	2332	0.10
321	370.30	4468	0.20
322	371.25	1831	0.08
323	372.20	5146	0.23
324	373.25	5093	0.23
325	374.25	54806	2.47
326	375.25	61887	2.79
327	376.20	144442	6.50
328	377.25	48025	2.16
329	378.20	271025	12.20
330	379.15	67760	3.05
331	380.20	56982	2.57
332	381.15	13270	0.60
333	382.15	2182	0.10
334	383.20	484	0.02
335	384.20	236	0.01
336	386.20	215	0.01
337	387.30	279	0.01
338	388.25	396	0.02
339	389.35	651	0.03
340	390.25	1290	0.06
341	391.35	1022	0.05
342	392.30	1966	0.09
343	393.35	1297	0.06
344	394.30	940	0.04
345	395.30	473	0.02
346	396.45	384	0.02
347	397.50	1070	0.05
348	398.45	585	0.03
349	399.45	317	0.01
350	400.20	370	0.02
351	401.20	289	0.01
352	402.20	250	0.01
353	403.20	257	0.01
354	404.45	336	0.02
355	405.40	1146	0.05
356	406.35	447	0.02
357	407.40	271	0.01
358	409.45	410	0.02
359	410.50	282	0.01
360	411.50	583	0.03
361	412.50	284	0.01
362	415.50	254	0.01
363	417.50	207	0.01
364	418.50	257	0.01
365	419.50	354	0.02
366	421.50	326	0.01
367	423.50	396	0.02
368	425.50	250	0.01
369	426.50	212	0.01
370	428.35	430	0.02
371	429.30	458	0.02
372	430.30	231	0.01
373	432.30	222	0.01
374	433.30	212	0.01
375	434.30	201	0.01
376	436.40	250	0.01
377	437.45	688	0.03
378	438.35	497	0.02
379	439.50	431	0.02
380	441.50	303	0.01
381	442.50	233	0.01
382	444.50	210	0.01
383	451.50	289	0.01
384	452.50	233	0.01
385	454.45	304	0.01
386	455.40	1105	0.05
387	456.35	499	0.02
388	457.10	265	0.01
389	458.15	443	0.02
390	459.10	218	0.01
391	460.20	433	0.02
392	462.20	287	0.01
393	463.40	473	0.02
394	464.40	252	0.01
395	466.40	212	0.01

3 / 3

Mass chart of compound **16**
